# The Effect of the Environmental Temperature on the Adaptation to Host in the Zoonotic Pathogen *Vibrio vulnificus*

**DOI:** 10.3389/fmicb.2020.00489

**Published:** 2020-03-27

**Authors:** Carla Hernández-Cabanyero, Eva Sanjuán, Belén Fouz, David Pajuelo, Eva Vallejos-Vidal, Felipe E. Reyes-López, Carmen Amaro

**Affiliations:** ^1^Departamento de Microbiología y Ecología and Estructura de Recerca Interdisciplinar en Biotecnologia i Biomedicina, Universitat de València, Valencia, Spain; ^2^Department of Cell Biology, Physiology and Immunology, Universitat Autònoma de Barcelona, Barcelona, Spain; ^3^Centro de Biotecnología Acuícola, Facultad de Química y Biología, Universidad de Santiago de Chile, Santiago, Chile

**Keywords:** *V. vulnificus*, temperature, host adaptation, transcriptome, microarray

## Abstract

*Vibrio vulnificus* is a zoonotic pathogen that lives in temperate, tropical and subtropical aquatic ecosystems whose geographical distribution is expanding due to global warming. The species is genetically variable and only the strains that belong to the zoonotic clonal-complex can cause vibriosis in both humans and fish (being its main host the eel). Interestingly, the severity of the vibriosis in the eel and the human depends largely on the water temperature (highly virulent at 28°C, avirulent at 20°C or below) and on the iron content in the blood, respectively. The objective of this work was to unravel the role of temperature in the adaptation to the host through a transcriptomic and phenotypic approach. To this end, we obtained the transcriptome of a zoonotic strain grown in a minimum medium (CM9) at 20, 25, 28, and 37°C, and confirmed the transcriptomic results by RT-qPCR and phenotypic tests. In addition, we compared the temperature stimulon with those previously obtained for iron and serum (from eel and human, respectively). Our results suggest that warm temperatures activate adaptive traits that would prepare the bacteria for host colonization (metabolism, motility, chemotaxis, and the protease activity) and fish septicemia (iron-uptake from transferrin and production of *O*-antigen of high molecular weight) in a generalized manner, while environmental iron controls the expression of a host-adapted virulent phenotype (toxins and the production of a protective envelope). Finally, our results confirm that beyond the effect of temperature on the *V. vulnificus* distribution in the environment, it also has an effect on the infectious capability of this pathogen that must be taken into account to predict the real risk of *V. vulnificus* infection caused by global warming.

## Introduction

*Vibrio vulnificus* is a worldwide-distributed aquatic pathogen that inhabits waters of salinity between 0.3 and 2.5% located in tropical, subtropical and temperate areas. The species can be isolated from coastal ecosystems when water temperature is between 10 and 30°C (Baker-Austin and Oliver, 2018). In fact, environmental temperature determines the life strategy of *V. vulnificus* in the aquatic ecosystem as this species enters into the viable but non-culturable (VBNC) state below 4°C and multiplies in the water column (if nutrients are available) above 18°C with an optimum above 25°C (Oliver, 2015). There is a strong scientific evidence suggesting that global warming is increasing the sea surface temperature, which is about 1°C higher than 100 years ago^1^. It is also predicted that the melting of the ice at the poles will produce a decrease in the seawater salinity around the world. Given that *V. vulnificus* grows preferentially in warm brackish water (1–2% NaCl) at temperatures above 18°C, the warming of marine environments together with the reduction in salinity will result in larger populations of these bacteria in coastal waters and, therefore will increase the risk of *Vibrio* infections (Le Roux et al., 2015).

*Vibrio vulnificus* is a highly heterogeneous species that comprises avirulent and virulent isolates for multiple hosts. The species has been divided in five phylogenetic lineages and one pathovar (pv. *piscis*) from a phylogenomic study based on the SNPs present in the core genome (Roig et al., 2018). Within the lineage II, there is a worldwide distributed clonal-complex with the unique ability to infect homeothermic (humans) and poikilothermic animals (fish and shrimps; main host the eel) (Amaro et al., 2015; Oliver, 2015). This is the sole group within the *Vibrio* genus that has been related to true zoonotic cases (Amaro et al., 2015; Oliver, 2015). This clonal-complex can infect humans by contact with seawater or fish (mainly diseased fish) causing severe wound infections that can lead to septicemia and death at risk patients (mainly those that present high levels of free iron in blood due to different predisposing underlying diseases such as hemochromatosis) (Horseman and Surani, 2011; Oliver, 2015; Amaro et al., 2015). The disease caused in fish is known as warm-water vibriosis since the most serious outbreaks always occur at temperatures above 25°C (Amaro et al., 2015). Warm-water vibriosis is an acute hemorrhagic septicemia that takes place in farms of eels and tilapia as epizootic or outbreaks of high and medium mortalities depending on water temperature (Fouz and Amaro, 2002; Fouz et al., 2006; Amaro et al., 2015). Epizootiological studies performed at laboratory scale confirmed that the pathogen virulence for eels is dependent on water temperature: maximal at 28°C (the maximum tested temperature) and minimal (no virulence at all) at 20°C and below (Amaro et al., 1995).

In previous studies, we performed a transcriptomic analysis of this pathogen grown in a minimal medium, with and without iron, and in the serum of its most susceptible hosts (Pajuelo et al., 2016; Hernández-Cabanyero et al., 2019). Those studies confirmed that iron in serum is one of the external signals triggering a host-adapted virulent phenotype in the pathogen. This response includes the production of the two main toxins of the species, RtxA1 and VvhA, together with a host-specific protective envelope to resist the host innate immunity (including nutritional immunity, complement system and phagocytosis). In case of human infection, the envelope is enriched in capsule; while in case of eel infection it is enriched in high molecular weight (HMW) and medium molecular weight (MMW) *O*-antigen together with two iron regulated outer membrane proteins (IROMPs), Ftbp and Fpcrp (Pajuelo et al., 2016; Hernández-Cabanyero et al., 2019). These two proteins constitute a kit for survival in fish blood that is encoded in a transmissible virulence plasmid (pVvbt2) (Pajuelo et al., 2015; Hernández-Cabanyero et al., 2019).

In this work, we hypothesized that environmental temperature (either water and/or host) could also contribute to the generation of the virulent phenotype in *V. vulnificus*. In fact, *V. vulnificus* is able to cause severe septicemia at both 28°C (in warm fish species) and 37°C (in humans). Although temperature-dependent gene expression has been widely studied in other bacterial species (Gao et al., 2006; Wang et al., 2017; Matanza and Osorio, 2018; Lages et al., 2019), to our knowledge this is the first time that the global transcriptomic response to an increase in the environmental temperature is analyzed in *V. vulnificus*. Nevertheless, it is well known that temperature impacts *V. vulnificus* growth ability (Kim Y. W. et al., 2012) and the expression of some virulence factors such as the major protease (Vvp), iron uptake systems (HupA and VuuA) and quorum sensing (McDougald et al., 2001; Oh et al., 2009; Kim et al., 2016; Elgaml and Miyoshi, 2017).

Taken all together, the aim of this work was to find out the role of temperature in the generation of the virulent phenotype in *V. vulnificus*, using a transcriptomic and phenotypic approach. For this purpose, we grew a representative strain of the zoonotic clonal-complex (CECT4999, hereafter R99) in a minimal medium (CM9) at three infective temperatures: 25°C (outbreaks of medium mortality in fish farms), 28°C (outbreaks of high mortality), and 37°C (human host body temperature). A non-infective temperature, 20°C (control), was also assessed (Amaro et al., 1995, 2015; Oliver, 2015). Then, the bacterial transcriptome was obtained by microarray hybridization followed by the bioinformatics analysis (Pajuelo et al., 2016). The transcriptomes were compared between them and also to those obtained in previous studies: iron stimulon and fur regulon (Pajuelo et al., 2016); and eel and human serum stimulons (Hernández-Cabanyero et al., 2019). Finally, the transcriptomic results were confirmed by RT-qPCR and phenotypic tests. Our results show that environmental temperature, either in the water or in the host body in the moment of infection, contributes to the pathogen’s pre-adaptation to a within-host survival by activating cellular mechanisms that prepare the bacterium for the subsequent invasion and survival in host blood. Therefore, under infective temperatures, bacterial fitness is enhanced by an increase in metabolic abilities, motility, chemotaxis, and protease production, accompanied by the production of a partially protective envelope. Nevertheless, temperature is not enough to trigger a host-adapted virulent phenotype, for which host and iron markers are essential.

## Materials and Methods

### Bacterial Strains and Growth Conditions

R99 strain, a representative strain of the zoonotic clonal-complex (Roig et al., 2018), was routinely grown on Tryptone Soy agar supplemented with 1% NaCl (TSA-1) and M9 supplemented with 0.2% casamino acids (CM9) (Miller, 1972). The inoculum for assays was prepared by inoculating 5 ml of media with bacteria from overnight cultures in a ratio 1:100 (v/v) and incubated with agitation (100 rpm) at 20, 25, 28, and 37°C. Drop plate counts on TSA-1 (Hoben and Somasegaran, 1982) and Abs_625_ measurements were used to follow bacterial growth.

### Transcriptomic Experiments

#### Microarray Analysis

Bacterial transcriptome was obtained as previously described (Pajuelo et al., 2016). Briefly, total RNA from mid-log phase cultures (Abs_625_ 0.3: 19 h cultures grown at 20°C; 8 h cultures grown at 25°C; and 6 h cultures grown at 28 and 37°C) was extracted using NucleoZOL RNA isolation product (Macherey-Nagel). The RNA was then treated with TURBO^TM^ DNase (Ambion) and cleaned with the GeneJET RNA Cleanup and Concentration Micro kit (Thermo Scientific). RNA concentration and integrity were measured using the Nanodrop 2000 spectrophotometer (Thermo Fisher) and the 2100 Bioanalizer (Agilent), respectively. Only those samples with a RIN ≥ 7.5 were selected to obtain labeled cDNA using the Low Input Quick Amp Labeling kit. The resultant cDNA was hybridized with the R99-specific microarray (Pajuelo et al., 2016) which contains probes for the 4,553 predicted coding sequences in the R99 genome (Pajuelo et al., 2016). Then, the slides were scanned with the Agilent DNA Microarray Scanner (G2505B). Raw data were obtained with Agilent’s Feature Extraction software version 10.4.0.0 (Agilent Technologies). The extracted data were analyzed with Genespring 14.5 GX software (Agilent technologies) as previously specified (Pajuelo et al., 2016). Student’s *t*-test (*P* < 0.05) available in Genespring software was applied to reveal the bacterial differentially expressed genes (DEGs) in CM9 at 25°C (compared to that at 20°C [25 vs. 20]), 28°C (compared to that at 20°C [28 vs. 20]) and at 37°C (compared to that at 20°C [37 vs. 20]). Results are presented as fold change mean from three independent biological samples. Venn diagrams were created using the free software available at Bioinformatics and Evolutionary Genomics group at Ghent University^2^.

#### RT-qPCR

RNA samples, obtained in the same conditions as for the microarray assays, were used to produce cDNA with Maxima H Minus Reverse Transcriptase (Thermo Scientific). The qPCR was performed on the cDNA to calculate the relative expression of selected genes (Table 1) as the fold induction using the 2^–ΔΔCt^ method and *recA* as the reference gene (Livak and Schmittgen, 2001). Primers used in this study are listed in Supplementary Table S1.

**TABLE 1:** Microarray validation by RT-qPCR.

Comparison^a^	Gene	Fold change^b^	
		
		Array	RT-qPCR
25°C vs. 20°C	*ftbp*	2 (+)	1.3 (=)
	*cpsA*	2 (+)	4.9 (+)
	*vvp*	–	1.5 (=)
	*vvhA*	–	1.6 (=)
	*rtxA1*_3_	–	1.9 (=)
28°C vs. 20°C	*flp*	14 (++)	3.6 (+)
	*malG*	−3.1 (−)	−1.2 (=)
	*vvp*	–	1.7 (=)
	*vvhA*	–	1.3 (=)
	*rtxA1*_3_	–	1.9 (=)
37°C vs. 20°C	*vpsT*	5.1 (+)	3.6 (+)
	*ktrA*	3.4 (+)	1.6 (=)
	*vvp*	–	1.9 (=)
	*vvhA*	–	1.7 (=)
	*rtxA1*_3_	–	1.4 (=)

*Comparison of fold change values obtained by array and RT-qPCR using the *V. vulnificus* R99 strain. In case of RT-qPCR, results were obtained using *recA* as reference gene. The fold induction (2^–ΔΔCt^) for each gene was calculated. ^a^RNA samples were obtained from mid-log phase cultures in CM9 at 20, 25, 28, and 37°C by using NucleoZOL (Macherey-Nagel), TURBO^TM^ DNAse (Ambion) and GeneJET RNA Cleanup and Concentration Micro kit (Thermo Scientific). ^b^Qualitative classification of the fold change values: =, −2 ≤ X ≤ 2; +, 2 ≤ X ≤ 10; ++, 10 ≤ X ≤ 25; −, −10 ≤ X ≤ −2. –, not detected as differentially expressed by the microarray analysis.*

### *In vitro* Assays

#### Biofilm Production

R99 was grown in glass tubes with 2 ml of CM9 at 20, 25, 28, and 37°C for 24 h. Biofilm production was quantified by staining with crystal violet as previously described by Jones et al. (2008).

#### Motility

Motility was assayed on motility agar (MA [CM9 0.3% agar (wt/vol)] plates as previously described by Pajuelo et al. (2016). Briefly, plates were inoculated with 5 μl from an exponential phase culture and incubated at 20, 25, 28, and 37°C for 24 h. Then, the motility rate was calculated as the rate between the surface of the bacterial colony on the plate (mm^2^) and the number of bacteria forming the colony (log CFU).

#### Proteolytic and Hemolytic Activity

The extracellular products (ECPs) from R99 strain were obtained from 24 h cultures on CM9-agar plates (1.5% agar [wt/vol]) at 20, 25, 28, and 37°C according to Biosca and Amaro (1996). Proteolytic and hemolytic activities in ECPs was evaluated as previously described by Valiente et al. (2008b), either using azocasein (Sigma) as substratum (protease) or seeding two-times dilutions on agarose-erythrocytes (5%) (bovine erythrocytes from Sigma) plates. Proteolytic activity was calculated as proteolytic units (PU) produced in each condition as described by Miyoshi et al. (1987) and hemolytic activity as the inverse of the highest dilution giving a positive result on the plate. In parallel, the hemolytic activity of live bacteria was determined as described by Hernández-Cabanyero et al. (2019).

#### Chemotaxis

Eel skin mucus was collected by placing non-anesthetized eels in empty sterile flasks, and the secreted material was recovered and filtered through 0.8 and 0.45 μm pore-size membranes (Millipore) (Esteve-Gassent et al., 2003). The chemotaxis toward eel skin mucus was determined at 20, 25, 28, and 37°C by using the capillary assay described by Larsen et al. (2001). Briefly, capillary tubes (100 μl pre-calibrated capillaries; BRAND) were filled with eel skin mucus or chemotaxis buffer (ChB) (PBS + 0.01 mM EDTA) (control condition). Then, the capillaries were place in eppendorfs containing 0.5 ml of 1 × 10^8^CFU/ml bacterial suspension in ChB from mid-log phase R99 cultures in CM9 at 20, 25, 28, or 37°C. Eppendorfs together with capillaries were incubated for 35 min at the corresponding temperature and the number of bacteria inside the capillaries was determined on TSA-1 by the drop plate method. The chemotaxis toward eel skin mucus was expressed as the chemotactic response defined as the ratio between bacterial numbers in the corresponding capillaries vs. control capillaries.

#### Surface Cell-Associated Polysaccharides

Crude fractions of cell-associated polysaccharides (LPS plus capsule) were obtained from mid-log phase cultures of R99 strain in CM9 at 20, 25, 28, and 37°C as described by Hitchcock and Brown (1983). Total polysaccharide concentration was determined with the Total Carbohydrate Assay Kit (BioVision) and 10 μg of cell-associated polysaccharides of each condition were separated by SDS-PAGE, transferred onto a PVDF membrane and subjected to immunoblot analyses according to Pajuelo et al. (2016).

#### Statistical Analysis

All the results are represented as mean ± standard deviation from three independent biological experiments. Statistical differences were tested with GraphPad software (version 7) by using one-way ANOVA. A *P* < 0.05 was considered significant.

## Results

### Bacterial Growth

**FIGURE 1:**
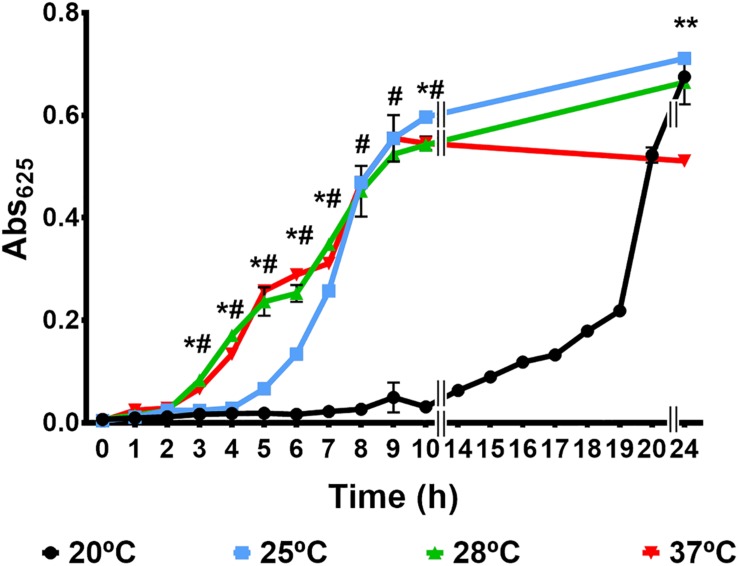
Growth of *V. vulnificus* in a minimal medium at different incubation temperature. R99 strain was inoculated in CM9 and incubated with agitation at 20, 25, 28, and 37°C. Growth was followed by measuring the Abs_625_ at regular time intervals. #: significant differences (*P* < 0.05) in Abs_625_ between 20 and 28°C. *: significant differences (*P* < 0.05) in Abs_625_ between 25 and 28°C. *^∗^: significant differences (*P* < 0.05) in Abs_625_ between 37 and 28°C.

Figure 1 shows the growth curves obtained at the different tested temperatures. The pathogen presented growth patterns statistically different at 20 vs. 25°C, 20 vs. 28°C, and 25 vs. 28°C but not at 28 vs. 37°C. Temperature mainly affected the lag phase of growth, whose duration was longer at 20°C (14 h) followed by 25°C (5 h), and shorter and similar at 28 and 37°C (around 2 h). The bacterial numbers achieved at 24 h post-incubation were around 1 × 10^9^ CFU/ml at 20, 25, and 28°C, and around 1 × 10^8^ CFU/ml at 37°C, suggesting that the death phase is accelerated at this temperature. Interestingly the maximum population size was similar regardless the incubation temperature (around 10^9^ CFU/ml).

### Transcriptomic Results

**FIGURE 2:**
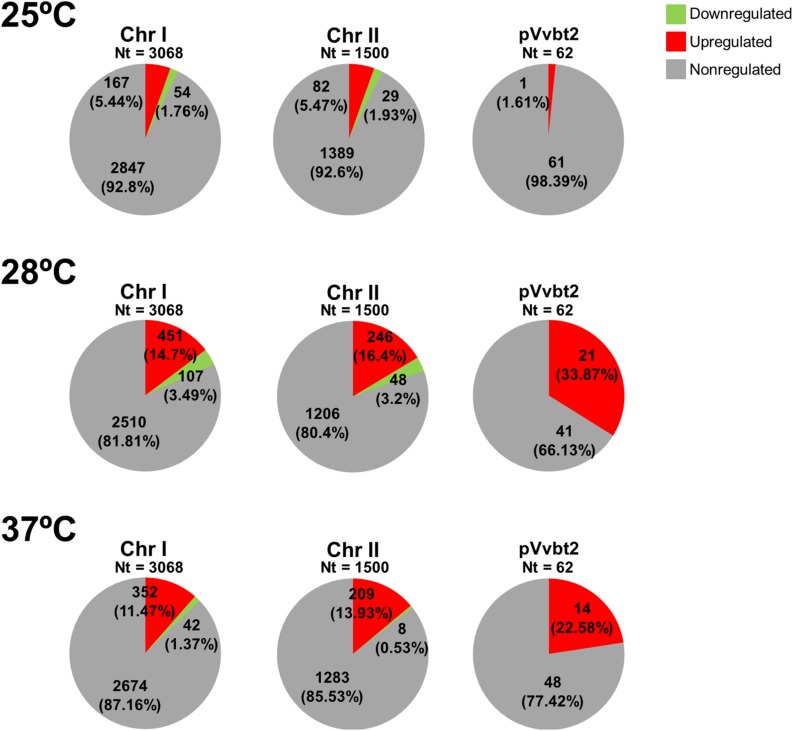
Graphical representation of DEGs distribution among *V. vulnificus* genome in response to temperature. Figure shows the DEGs according to their distribution per regulation category (upregualted, downregulated, or non-regulated) and replicon (two chromosomes and one plasmid). The different categories are represented by color.

**FIGURE 3:**
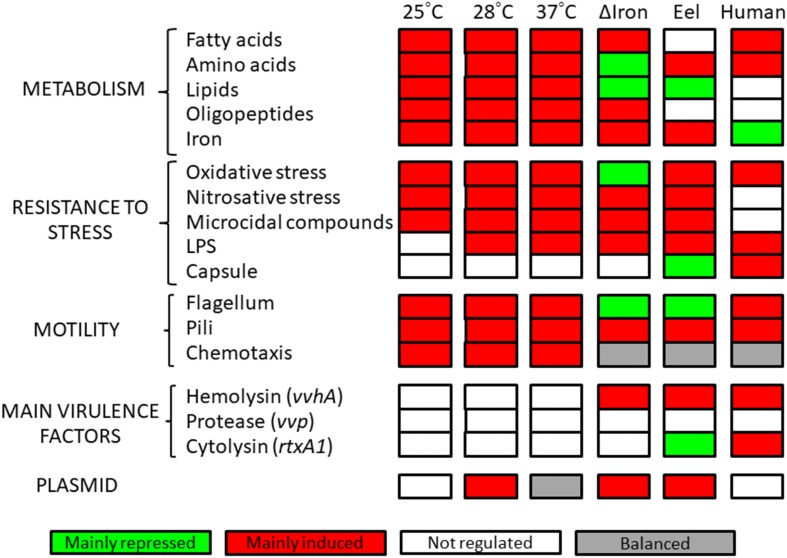
Cellular processes modulated by temperature. Categories of cellular processes affected at each temperature. Cellular processes differentially expressed at iron stimulon, fur regulon and serum regulons are also included.

We compared the DEGs at infective temperatures (25, 28, and 37) with those expressed at the non-infective temperature (20°C) (Supplementary Tables S2–S4). Figure 2 shows the number and distribution of the DEGs in the genome per temperature, Figure 3 shows the main cellular processes modulated at each temperature, Table 1 provides a comparison between fold change values obtained by hybridization with the R99-specific microarray and by RT-qPCR, and Table 2 shows the selected DEGs as relevant per temperature.

**TABLE 2:** Selected DEGs for *V. vulnificus* grown at infective temperatures (25, 28, and 37°C) vs. non-infective temperature (20°C).

Gene(s)^a^	25°C^b^	28°C^c^	37°C^d^	Putative function/process^e^
**Metabolic and nutrient transport and utilization genes (including iron)**				
*fadH*	11.3	–	2.3	Unsatturated fatty acids metabolism
Peptide ABC transporters#	10.4-2.4	–	3.9	Peptide transport
*ilvE, ilvG, ilvI*	7.8-2.5	–	2.1	Amino acid biosynthesis
*trpCD*	6.1-4	–	2.8	Amino acid biosynthesis (tryptophan)
C4-dicarboxylate transporters#	6-2.4	–	20.7-2.9	Dicarboxilate transport
*argG, argI*	5-2.1	2.1	–	Amino acid metabolism
Ferric iron ABC transporter*#	3.7	3.4-2.3	2.6	Ferric iron transport
Long-chain fatty acid transport protein	3.3	–	–	Fatty acids transport
Vulnibactin biosynthetic genes*#	3-2.3	2.9-2.5	2.3	Vulnibactin biosynthesis and transport
*hisBD*	2.7	−2.1	–	Amino acid biosynthesis
*fabGH*	2.4	–	2.4	Fatty acids metabolism
Amino acid ABC transporter*	2.3	5.5-2.8	13.8-6.2	Amino acid transport
*metB, metK, metL*	2.3-2.1	8.5	2.8	Amino acid biosynthesis
L-serine dehydratase	2.3	–	–	Amino acid metabolism
potB, potD*#	2.2	2	–	Polyamine transport
PTS system, *N*-acetylglucosamine-specific IIB, IID components*#	2.1	–	3.5	Aminosugar transport
Nitrate ABC transporter	2.1	–	–	Nitrogen metabolism
ftbp*#	2	2	2.8	Fish transferrin binding protein
*rbsABCD*#	−2.6	−(3.4-3.7)	6.6-3.4	Ribose ABC transport system
Short chain fatty acids transporter	−2.7	−2.4	–	Fatty acids transport
*malG*	−2.9	−3.1	–	Maltose/maltodextrin ABC transporter
*prsA*	−3.1	−2.6	–	Ribose metabolism
Chitinase proteins	–	22.3-2.5	8.8	Chitinase activity
*serB*	–	10.6	9.8	Amino acid biosynthesis
*pflA*	–	8.4	–	Glucose metabolism
Succinate-semialdehyde dehydrogenase [NADP+]	–	8.4	–	Amino acid degradation
nupC*#	–	6.7	8.1	Permease for nucleoside uptake
dns*#	–	5.7	–	Degradation of DNA for nutrient uptake (competence related)
Zinc ABC transporter	–	4.5-3.5	–	Zinc transport
Lipase-related proteins	–	4.2-2.7	8-5.3	Extracellular lipid utilization
Phosphate ABC transporter#	–	3.8-2.6	6.6	Phosphate transport
*trkA, trkH*	–	3.1	3.9-2.4	Potassium uptake
*oppBCD, oppF*	–	3-2.7	7.9-2.1	Oligopeptide transport system permease
*ktrA*#	–	2.8	3.4	Potassium uptake
*thrC*	–	2.7	–	Amino acid iosynthesis
*dctQ*#	–	2.3	6.5	TRAP dicarboxylate transporters
*proA*	–	2.3	–	Amino acid biosynthesis
Ferrous iron transporter B	–	2	–	Ferrous iron transport
*acp12*	–	−(2.6-3.7)	–	Fatty acids biosynthesis
*glgX*#	–	–	12.8	Glycogen debranching enzyme
*hmgA*	–	–	10-4	Amino acid degradation
*citAB*	–	–	8.3-2.8	Citrate metabolism
**Anaerobic respiration**				
Nitrate reductase cytochrome c550-type subunit*	2.7	–	3	Nitrite reductase complex subunit
napC, napE, napGH*#	–	22.5-6.7	3.9-2.2	Subunit of the periplasmic nitrite reductase complex
Nitrite reductase subunits	–	3.5-2.8	–	Nitrite reductase complex
*nrfF*	–	–	23.3	Formate-dependent nitrite reductase complex
**Stress response and defense mechanisms**				
Anaerobic glycerol-3-phosphate dehydrogenase subunits (B, C)#	4.6-2.2	–	4.4-3.7	Phospholipid biosynthesis/membrane regeneration
*S*-(hydroxymethyl)glutathione dehydrogenase#	3	2.3	–	Resistance to oxidative stress
*aphF*	2.8	–	–	Alkyl hydroperoxide reductase protein F. Resistance to oxidative stress
Glutathione S-transferase#	2.7	4.5	–	Resistance to oxidative stress
*uspA*	2.7	–	–	Universal stress protein A, involved in DA-damage resistance
mutS*	2.5	–	–	DNA mismatch repair protein
Superoxide dismutase [Cu–Zn] precursor	2.3	–	–	Resistance to oxidative stress
*plsXY*#	2.2	4.9	3.3	Phospholipid biosynthesis/membrane regeneration
Phosphoglycerol transferase I*#	2.2	4.2	–	Phospholipid biosynthesis/membrane regeneration
YfgC precursor*	2.1	–	–	Outer membrane integrity
*cydD*	2.1	–	–	Glutathione transport
Permease of the drug/metabolite transporters (DMT)#	2	4.8-2.1	11.5-2.3	Resistance to microcidal compounds
Glycerophosphoryl diester phosphodiesterase#	2	–	3.1	Phospholipid biosynthesis/membrane regeneration
*sspAB*	−(2-2.3)	−2.1	–	Stringent starvation proteins
rseABC*#	–	9.5-6.2	–	Negative regulatory proteins for RpoE, a sigma factor for envelope stress response
nsrR*#	–	8.2	–	Repressor for resistance to nitrosative stress
*cmeB*	–	7.2	–	Drug efflux system
clpB*#	–	5.7	–	Stress-induced chaperone
Formate efflux transporter*#	–	4.8	–	Resistance to microcidal peptides
cspD*	–	3.7	–	Cold shock proteins, involved in stress caused by membrane damage
Manganese superoxide dismutase	–	3.5	11	Resistance to oxidative stress
norR*	–	3.4	–	Anaerobic nitric oxide reductase transcription regulator
uvrC*#	–	3.2	4.5	Excinuclease ABC subunit C for DNA repair
*marR*	–	3	6.6	Multiple antibiotic resistance protein
*htpG*	–	3	–	Chaperone protein
*msrAB*#	–	2.7	2.3	Peptide methionine sulfoxide reductase involved in reparation of oxidized proteins
ohr*	–	−2.6	-3.65	Repressor for organic hydroperoxidase resistance
*degQ*#	–	–	2.1	Outer membrane integrity
**Flagellum, pili and chemotaxis**				
Methyl-accepting chemotaxis protein*#	8.5-2.4	12.8-2.5	15.5-2	Chemotaxis
mshH, mshJK, mshOP*#	2.7	3.2-2.1	4.2-2.1	Pili MSHA biosynthesis
*fleQ, fleS*	2.4-2.2	2	4.1-3.1	Flagellar regulatory protein
flgA, flgHI, flgL, flgT*#	2.2-2.1	4.1	11.9-3.2	Flagellar basal-body rod proteins
tadBCD, tadZ*	2.2-2	14.1-3.2	10	Flp pili assembly
fliF, fliM*#	2	2.2-2.1	3.6-2.6	Flagellar motor activity
Probable type IV pilus assembly FimV-related	−2.4	−2.4	–	Pili MSHA
rpoN*#	–	27.1	3.8	RNA polymerase sigma factor
*flgN*	–	8.7	2.1	Flagellar biosynthesis protein
flgK, flgM*#	–	8.5	16	Hook associated protein
motAB*#	–	7.5-2.8	3.6	Flagellar motor rotation protein
fleN*#	–	5.8	2.1	Flagellar synthesis regulator
RNA polymerase sigma factor for flagellar operon	–	5.4	–	Flagellar biosynthesis
*acfD*	–	4	–	Accessory colonization factor, putatively involved in motility
*fliL*	–	3.7	–	Controls rotational direction of flagella during chemotaxis
flaD. flaFG*#	–	3.6-2.3	6.5-4	Flagellin protein
*cheD*	–	3.3	–	Chemotaxis protein
Chemotaxis regulator#	–	3.1	5.8	Transmits chemoreceptor signals to flagellar motor
**LPS and capsule**				
*cpsABC*#	2	5.5-3.3	–	Capsule biosynthesis
*sypAB, sypR*	–	5.1-2.3	15.8-5.2	Capsule biosynthesis
*wza*#	–	−2.8	–	LPS biosynthesis
*galE*	–	–	4.7	*O*-antigen biosynthesis
*lptA*	–	–	2.3	LPS biosynthesis
**Transcriptional regulators**				
Nitrogen regulation protein NR(I)*	3.1	−2.7	–	Nitrogen starvation
tetR*#	2.6	7.9	–	Involved in transcriptional control of multidrug efflux pumps, pathways for the biosynthesis of antibiotics, response to osmotic stress and toxic chemicals, control of catabolic pathways, differentiation processes, and pathogenicity
*rpoS*	2.5	–	–	Stress and metabolism management
*arcA*#	−2.4	−2.6	–	Repressor for aerobic metabolism
uxuR*#	–	9	–	Repressor for oligogalacturonide metabolism
mall*#	–	4.3	–	Maltose regulon repressor protein
*deoR*	–	4	–	Repressor of deoxyribose operon
vpsT*#	–	3.7	5.1	Repressor of biofilm and biofilm related polysaccharide formation
luxO*#	–	3.6	–	Involved in quorum sensing pathways
phoR*#	–	2.9	–	Histidine kinase for PhoB, involved in phosphate metabolism
luxU*#	–	2.9	–	Phosphorelay protein involved in quorum sensing
CRP*	–	2.9	5	cAMP receptor protein, regulatory protein
*argR*	–	2.5	–	Repressor of arginine metabolism
luxQ*#	–	−2.6	–	Autoinducer 2 sensor kinase/phosphatase, involved in quorum sensing
*smcR*#	–	–	2.2	Involved in quorum sensing pathways
fabR*#	–	–	−2.7	Repressor for unsaturated fatty acid biosynthesis

The value of fold change per gene at each infective temperature (25, 28, and 37°C vs. non-infective temperature [20°C]) is shown. Only genes with values of fold change −2 ≤ X ≤ 2 with a *p*-value cut-off of 0.05 were considered. ^a^Identified DEGs are indicated. ^b–d^Fold change value or range of values for each identified gene or group of related genes at 25°C^b^, 28°C^c^, and 37°C^d^. See Supplementary Tables S2–S4 for specific gene and fold change value. ^e^Putative function for selected genes and related process. *Differentially expressed in iron stimulon (Pajuelo et al., 2016). #Differentially expressed in eel or iron-overloaded human serum (Hernández-Cabanyero et al., 2019). –, not detected as differentially expressed.

**FIGURE 4:**
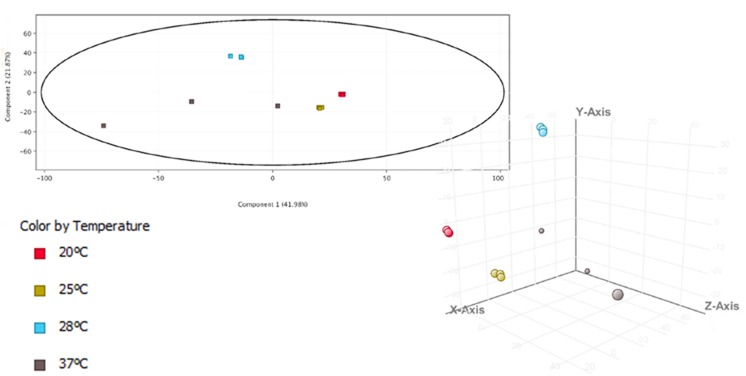
Principal component analysis (PCA) 3-D plot of temperature samples. The transcriptomic profile is represented by different colors.

The temperature stimulon, defined as the set of DEGs at the analyzed temperatures, totalized 1,375 genes (30.2% of genes in the genome) (Supplementary Table S5). This value was similar to that found in the iron stimulon (25.78%), Fur regulon (35.91%) and host-serum stimulons (26.35% for eel serum; 20.32% for risk patient serum) (Pajuelo et al., 2016; Hernández-Cabanyero et al., 2019) (Figure 2). In addition, around 40% of the chromosomal genes that belonged to temperature stimulon were also found in the rest of stimulons and/or regulons (Supplementary Figure S1), while less of 1% of the plasmid DEGs in the temperature stimulon belonged to another stimulon/regulon (results not shown). Globally, this result suggests that plasmid genes involved in response to temperature neither are controlled by Fur nor belong to the bacterial networks involved in response to iron or serum. The principal component analysis (PCA) showed the transcriptomic profiles clearly grouped in accordance to different temperatures (Figure 4), being those obtained at 20 and 25°C more similar to each other than those obtained at 28 and 37°C. In addition, the PCA showed that (i) the biological replicates obtained at each temperature were very similar to each other, with the exception of those obtained at 37°C, which showed greater variability; and (ii) the samples obtained at 28°C were the furthest from the rest. Precisely, the highest number of DEGs was obtained at 28°C (Supplementary Figure S1A and Figure 3), temperature at which the highest number of plasmid DEGs was detected with 21 genes upregulated (including *ftbp*, a gene encoding a fish transferrin binding protein essential for fish virulence, Pajuelo et al., 2015) and none downregulated (Figure 2). pVvBt2 is a plasmid essential for virulence given that it confers resistance to the innate immunity (nutritional immunity and complement system) of teleost fish (Lee et al., 2008; Hernández-Cabanyero et al., 2019). Therefore, this difference in plasmid gene expression could help to explain why the pathogen is more virulent for fish at 28 than at 25 or 20°C (Amaro et al., 1995, 2015).

Nutrient uptake and metabolism were among the cellular processes most strongly activated by an increase in temperature (Table 2 and Supplementary Tables S2–S4). For example, *dns*, encoding an extracellular nuclease (Blokesch and Schoolnik, 2008), together with *nupC*, which encodes a nucleoside uptake protein involved in *V. cholerae* fitness in nutrient limited environments (Gumpenberger et al., 2016), and multiple genes related to amino acid biosynthesis and transport (including genes for oligopeptide transport [*opp* genes]) as well as lipid and fatty acid metabolism and transport were upregulated at infective temperatures (Table 2). Accordingly, *fabR*, a repressor of unsaturated fatty acid metabolism was downregulated at 37°C. Finally, we found some genes involved in vulnibactin siderophore biosynthesis and iron transport (either ferrous or ferric iron), including the plasmid gene *ftbp*, upregulated at all the infective temperatures (Table 2). In accordance, *crp* (cyclic AMP [c-AMP] receptor protein) was upregulated at 28 and 37°C, a regulator reported to be responsible for positive control of iron uptake systems (Choi et al., 2006; Oh et al., 2009; Kim et al., 2016). Altogether, our results show that an increase in temperature leads to a metabolic shift as it is suggested by the upregulation of multiple genes involved in biosynthesis, degradation and transport of amino acids, fatty acids, lipids and iron, which would ensure bacterial growth and constitute an advantage for *V. vulnificus* at host physiological temperature during the infection. Curiously, and as an exception, we found transcriptomic evidence of maltose/maltodextrin transport downregulation at 25 and 28°C [a process previously found upregulated by iron and when the bacterium was grown in iron-overloaded human serum at 37°C (Pajuelo et al., 2016; Hernández-Cabanyero et al., 2019)], together with the upregulation of a gene for the maltose regulon repressor (*mall*) (Table 2).

Transcriptomic signs of stress response activated by *V. vulnificus* were obtained at all the assayed temperatures (Table 2). Although the DEGs involved in the stress response were not exactly the same at each of the assayed temperatures, the global response was very similar among them, and mostly enhanced at 28°C (Table 2). This stress response included the upregulation of defense mechanisms such as membrane protection and regeneration (phospholipid biosynthesis), resistance to oxidative and nitrosative environments (*aphF* [Alkyl hydroperoxide reductase protein F], a superoxide dismutase precursor, glutathione-related genes and nitrate reductase-related genes), together with the repression of *ohr* (encoding a repressor for oxidative stress resistance) as well as genes involved in resistance to microcidal compounds (Table 2). All these processes together comprise the bacterial ability to resist inside the host during the infection (Kaufmann and Dorhoi, 2016; Lim et al., 2017; Uribe-Querol and Rosales, 2017).

Motility and chemotaxis are bacterial virulence factors required for host colonization (Lee et al., 2004; Kim S. M. et al., 2012). Our transcriptomic results showed several flagella-related genes (including genes encoding flagellar hook proteins, flagellar basal-body proteins and flagellar motor activity proteins) and chemotaxis-related genes upregulated in all the assayed conditions (Table 2). Curiously, all these motility-related genes found upregulated by an increase in temperature were previously found downregulated in iron stimulon and eel serum, but upregulated in iron-overloaded human serum (Pajuelo et al., 2016; Hernández-Cabanyero et al., 2019).

Concerning attachment and biofilm formation, our transcriptomic results showed that bacterial MSHA and Flp pilus (*msh* and *tad* genes) (Watnick et al., 1999; Pu and Rowe-Magnus, 2018) were upregulated when temperature rises, as they were in iron deficiency and in eel serum (Table 2). Contrary, *vpsT*, encoding a repressor for biofilm production (Krasteva et al., 2010) was upregulated by temperature. This modulation suggests the activation of regulatory mechanisms in response to the upregulation of genes associated to attachment and biofilm formation.

Finally, we also found that multiple external envelope biosynthesis genes (LPS and capsule production), involved in resistance to host complement system (Amaro et al., 1994, 1997), were upregulated by an increase in temperature (Table 2), although not as many as those previously observed to be regulated by iron and host serum (Pajuelo et al., 2016; Hernández-Cabanyero et al., 2019). Interestingly, we did not find any of the major toxins or virulence master regulators differentially expressed in any of the comparisons performed, with the exception of the upregulation of quorum sensing regulators (*luxU* and *luxO* at 28°C and *smcR* at 37°C) and *tetR*, encoding a master regulator involved in transcriptional regulation of multidrug efflux pumps, antibiotics biosynthesis, response to osmotic stress, control of catabolic pathways and pathogenicity (Ramos et al., 2005), at 25 and 28°C (Table 2).

### Phenotypic Results

#### Biofilm Production

Our transcriptomic results suggested changes in biofilm formation with an increase in temperature (Table 2). Therefore, we phenotypically tested the bacterial ability to form biofilm by staining biofilms with crystal violet after 24 h of growth and measuring Abs_540_. No significant differences were found for biofilm production between conditions although a slight increase in biofilm quantity with rising temperature was observed (Table 3).

**TABLE 3:** Phenotypic characterization of *V. vulnificus* grown at different temperatures.

Condition	Biofilm (Abs_540_)^a^	Motility rate^b^	Cellular-associated polysaccharides (μg/10^8^ cells)^c^	Proteolytic activity (PU)^d^
20°C	0.027 ± 0.002	0.24 ± 0.02*	87.67 ± 0.37*	12.09 ± 6.65*
25°C	0.034 ± 0.005	1.56 ± 0.08#	95.37 ± 1.32*#	6216.17 ± 250.55#
28°C	0.035 ± 0.016	1.40 ± 0.31#	103.55 ± 2.77#	6498.76 ± 210.18#
37°C	0.066 ± 0.048	2.46 ± 0.60*#	108.68 ± 5.92#	4021.47 ± 181.49*#

^a^For biofilm quantification bacteria were grown in glass tubes for 24 h. Then, planktonic bacteria were eliminated and biofilm was quantified after staining with crystal violet by measuring absorbance at 540 nm (Abs_540_). ^b^Motility was measured as motility rate (colony surface in mm^2^/log of colony bacterial number in CFU). The assay was performed on plates of CM9-agar (0.3%) as described by Pajuelo et al. (2016). ^c^Total polysaccharide concentration was determined with Total Carbohydrate Assay Kit (BioVision) as described by the manufacturers. ^d^Proteolytic activity in ECPs, obtained according to Biosca and Amaro (1996), was evaluated using azocasein (Sigma) as substratum (Valiente et al., 2008b). Proteolytic activity is expressed as proteolytic units (PU) produced in each condition (Miyoshi et al., 1987). In all cases results are presented as the averages ± standard deviation of three independent experiments. *: significant differences (*P* < *0.05*) at each temperature vs. 28°C. #: significant differences (*P* < *0.05*) at each temperature vs. 20°C.

#### Motility

Flagella-related genes were the most abundant DEGs in our transcriptomic study, upregulated when incubation temperature increases (Table 2). We performed a motility assay and confirm that the motility rate of *V. vulnificus* significantly increases when the environmental temperature increases, being non-motile at 20°C (non-infective temperature), intermediate-motile at 25 and 28°C (without significant differences between these two temperatures) and high-motile at 37°C (Table 3).

#### Proteolytic and Hemolytic Activity

Vvp, the major exoprotease of *V. vulnificus*, is involved in adhesion and mucosal colonization and therefore, is considered an important virulence factor for both fish and human colonization (Amaro et al., 1995; Paranjpye and Strom, 2005; Valiente et al., 2008b; Jones and Oliver, 2009). Although we did not find *vvp* differentially expressed in any of the transcriptomic comparisons performed, we found upregulated at optimal temperatures for eel and human vibriosis (28 and 37°C, respectively) *crp*, encoding c-AMP receptor protein, and quorum sensing regulators, both described as inducers of Vvp activity (Elgaml and Miyoshi, 2017) (Table 2). Therefore, we checked the effect of temperature on protease production by *V. vulnificus* through the measuring of azocasein proteolysis at 20, 25, 28, and 37°C. Our results revealed that at 20°C there was no proteolytic activity and this activity increases with temperature, achieving the major point of activity at 28°C and again decreasing at 37°C (Table 3). Our results are in accordance with previous studies of temperature dependent regulation of Vvp as previous studies described that this protease activity is efficient at 26°C (Elgaml and Miyoshi, 2017). On the contrary, hemolytic activity was not affected by temperature, as we did not find differences in neither live bacteria nor ECPs hemolytic activity among the analyzed temperatures (results not shown).

#### Chemotaxis

**FIGURE 5:**
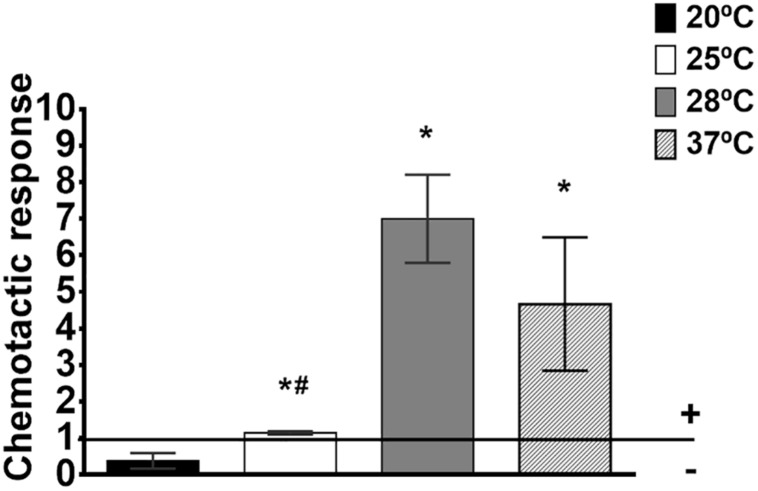
Chemotaxis toward eel skin mucus in *V. vulnificus*. Chemotaxis was measured as Chemotactic response (ratio of bacterial numbers in eel skin mucus-capillaries vs. control-capillaries [containing Chemotaxis buffer (ChB)]). Horizontal line marks the borderline between positive and negative chemotaxis. *: significant differences in chemotaxis response toward eel skin mucus at each temperature vs. 20°C (*P* < 0.05). #: significant differences in chemotaxis response toward eel skin mucus between each temperature and 28°C (*P* < 0.05).

Among the most upregulated genes in response to temperature were those related to chemotactic response (Table 2). Given that both motility and proteolytic activity are temperature-dependent (Tables 2, 3), and motile *V. vulnificus* cells which produce Vvp are positively chemo-attracted by eel mucus (Valiente et al., 2008b), we tested *V. vulnificus* chemo-attraction toward eel skin mucus at 20, 25, 28, and 37°C. Interestingly, the pathogen only exhibits positive attraction to eel mucus at 28 and 37°C but not at 20 or 25°C (Figure 5), which agrees with the efficient temperatures for protease activity (28°C) and motility (37°C) (Table 3).

#### Surface Cell-Associated Polysaccharides

**FIGURE 6:**
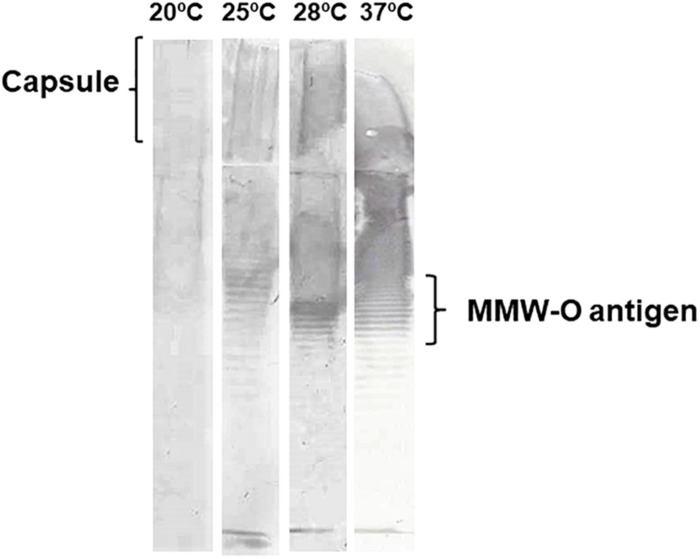
*Vibrio vulnificus* LPS and capsule profiles in response to temperature. R99 strain was grown until mid-log phase in CM9 at 20, 25, 28, and 37°C. Then, cell-associated polysaccharides (LPS + capsule) were extracted with the method of Hitchcock and Brown (1983) and quantified using the Total Carbohydrate Assay Kit (BioVision). Ten μg of cell associated-polysaccharides were separated by SDS-PAGE on discontinuous gels (4% stacking gel, 10% separating gel), transferred to a PVDF membrane, subjected to immunoblot analysis with antibodies against cell-envelopes of R99 according to Pajuelo et al. (2016). MMW, medium molecular weight.

We determined in previous studies that *V. vulnificus* external envelope production is regulated by iron concentration and seems to confer resistance to innate immunity in a host-dependent manner (Pajuelo et al., 2016; Hernández-Cabanyero et al., 2019). A few genes related to *O*-antigen (*galE*) and capsule biosynthesis (*cps* and *syp* genes) were upregulated in response to an increase in temperature, albeit they did not correspond to those previously obtained in response to iron and/or host sera (Table 2). In order to better understand how temperature could influence external envelope formation, cell-associated polysaccharides of *V. vulnificus* grown at 20, 25, 28, and 37°C were extracted, quantified and analyzed. The quantity of cellular-associated polysaccharides (μg per 10^8^ cells) significantly increased with temperature until 28°C and from that on (37°C) the increase in temperature decreased polysaccharide concentration (Table 3). Accordingly, the cell-associated polysaccharide pattern also showed changes with temperature. This pattern can be only visualized by immunoblotting because neither the LPS nor the capsule are stained with silver (Amaro et al., 1992). Given that the quantity of polysaccharide per well was the same, the obtained results suggest that the immunogenicity of external polysaccharides seems to change with temperature: no band was immunostained at 20°C while when the *O*-antigen pattern at 25, 28, and 37°C was compared, we found the MMW *O*-antigen portion clearly increased at 28°C (Figure 6). Interestingly, MMW is the portion of the *O*-antigen directly involved in resistance to eel innate immunity (Valiente et al., 2008a). Similar changes affecting *O*-antigen pattern were also observed when the LPS from cells grown in eel serum (28°C) was compared with that obtained in iron-overloaded human serum (37°C), while the change observed in capsule production in presence or absence of iron was not reproduced by changes in temperature (Pajuelo et al., 2016; Hernández-Cabanyero et al., 2019). Altogether, these results suggest that temperature influences the LPS biosynthesis while capsule production is controlled by iron, among other factors, in *V. vulnificus*.

## Discussion

*V. vulnificus* is an opportunistic pathogen from brackish water ecosystems located in tropical, subtropical and temperate areas that is spreading to traditionally cold areas, such as the Baltic Sea or Greenland, due to the global warming (Motes et al., 1998; Baker-Austin et al., 2012; Oliver, 2015). This expansion is responsible for an unusually high number of infections affecting humans and fish that has been registered in these new areas in the last years (Baker-Austin and Oliver, 2018). In fact, the water temperature is a risk factor that enhances the occurrence of disease outbreaks caused by *Vibrio* species (Le Roux et al., 2015). There are numerous field data that relate water temperature with outbreak severity of *V. vulnificus* vibriosis in fish farms (Rodolfo Barrera, personal communication), data that have been probed at laboratory scale by infecting eels at different water temperatures (Amaro et al., 1995). These experiments demonstrated that the pathogen was avirulent at 20°C (or below) and caused septicemia in fish maintained over 22°C being moderately virulent at 25°C and highly virulent at 28°C (Amaro et al., 1995). Moreover, *V. vulnificus*, as a multi-host pathogen, is also able to infect humans and cause septicemia at 37°C (Horseman and Surani, 2011). On the other hand, we have demonstrated in previous studies that *V. vulnificus* upregulates a high virulent host-adapted phenotype in order to face the drastic change in environmental conditions upon host encounter (i.e., iron availability [low in healthy eels and high in risk human patients] and innate immunity [serum complement proteins]) (Pajuelo et al., 2016; Hernández-Cabanyero et al., 2019).

In this work, we hypothesized that temperature is one of the external signals, which together with iron, triggers virulence in *V. vulnificus*. In fact, an increase in environmental temperature activates virulence factors expression in other bacteria that are also accidental pathogens (Konkel and Tilly, 2000). To test our hypothesis, we analyzed the global transcriptome of *V. vulnificus* grown at infective (25, 28, and 37°C) vs. non-infective (20°C) temperature and confirmed the results by phenotypic assays. Finally, we compared the obtained temperature stimulon with the previously described in iron and host serum stimulons (Pajuelo et al., 2016; Hernández-Cabanyero et al., 2019).

**FIGURE 7:**
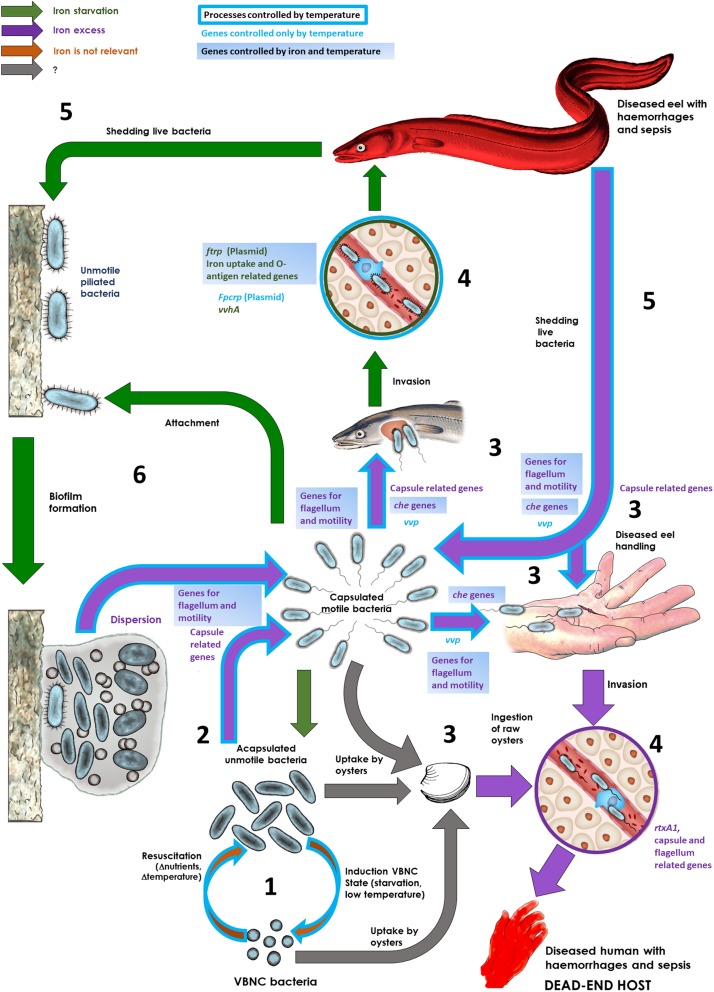
Life cycle of the zoonotic pathogen *V. vulnificus*: *role of iron and temperature*. This figure summarizes the role of iron levels and temperature in the surrounding environment in determining the life strategy of *V. vulnificus*. The main steps and processes as well as some genes involved are shown. **(1)** *Resuscitation and induction of the viable but non-culturable state (VBNC)*. As a free living form, the pathogen swifts between a VBNC and a vegetative state depending on nutrient availability as well as on water temperature and salinity. **(2)** *Capsule and flagellum production in the environment.* Vegetative bacteria produce a capsule and a polar flagellum when iron, and probably other nutrients, are available. The flagellum production is also controlled by temperature. **(3)** *Host colonization.* Motile/unmotile bacteria could be attracted by blood/mucus (chemotaxis) from their susceptible hosts (eels and humans with high iron-levels in the blood) and colonize a wound or fish mucus. Alternatively, bacteria can be uptaken by filtering organisms and infect humans by ingestion and colonize the intestine or can infect humans by diseased fish handling. **(4)** *Septicemia.* From the wound or mucosal tissue, the pathogen arrives to the bloodstream; in case of humans with high iron levels, the pathogen produces a capsule, multiplies and secretes the toxins VvhA and RtxA1 that cause the death by a toxic sepsis; in case of an eel, only the cells with the plasmid produce two iron-regulated outer membrane proteins, Fpcrp (fish phagocytosis complement resistance protein) and Ftbp (fish transferrin binding protein) that protect against innate immunity (in addition to an envelope enriched in *O*-antigen), multiply and secrete VvhA, which lyses erythrocytes, increases iron levels and, indirectly, actives the production of RtxA1, which causes the death of the fish by a toxic sepsis. **(5)** *Shedding bacteria to water.* Diseased fish liberate bacteria to water. If water is rich in iron, bacteria can infect humans (zoonosis). **(6)** *Biofilm formation and dispersion.* Bacteria could be attached to surfaces (including fish mucosae) and to form biofilms under iron restriction. Under iron excess, bacteria will be dispersed from the biofilms as capsulated motile bacteria. Figure modified from Pajuelo et al. (2016).

Figure 7 proposes a model on the role of environmental temperature in the life cycle and host-adaptation in *V. vulnificus* as well as highlights several genes that belong to the temperature-stimulon as putatively involved in some processes. This model integrates the results obtained in this work with those previously obtained about the role of iron and the host serum in the life strategy of this species inside and outside its main hosts (Pajuelo et al., 2016; Hernández-Cabanyero et al., 2019).

## Motility, Chemotaxis and Protease Activity

Motility and chemotaxis have been previously related to fish (mucus and blood) and human (blood) colonization by *V. vulnificus* (Valiente et al., 2008b). We found that both activities were controlled by the environmental temperature, as the bacteria were significantly more motile and chemo-attracted toward the eel mucus at the warmest temperatures. Attraction to mucus and blood by *V. vulnificus* is also dependent on *vvp*, a gene encoding the main exoprotease of this species, as the mutants deficient in this gene are unable to be attracted by mucine (the main component of mucus), fish mucus or blood (from humans and fish) (Valiente et al., 2008b). In fact, the protease is involved in mucine and hemoglobin degradation (Miyoshi et al., 1997; Valiente et al., 2008b) and therefore, could facilitate chemo-attraction toward mucosal surfaces and wounds. We phenotypically confirmed that protease activity was increased with temperature with a maximum at 28°C, followed by 37°C. This increase in activity should be related to a post-transcriptional activation rather than an increase in transcription level, as *vvp* was not differentially expressed with an increase in temperature. In any case, during host infection at warm temperatures (28°C) *V. vulnificus* would produce more active protease, which would favor colonization of mucosal epithelial tissues, and consequently, the infection. This high level of active protease, together with the chemo-attraction to mucus at 28°C but not at 20°C either at 25°C would provide evidence why the highly virulent phenotype for eels is expressed by *V. vulnificus* at 28°C. Several regulators related to motility, chemotaxis and protease production were also found to belong to the temperature stimulon and were regulated accordingly with the observed phenotype: i.e., upregulation of *vpsT*, encoding a repressor of biofilm formation, that promotes motility from biofilm dispersion (Krasteva et al., 2010); upregulation of *crp*, which encodes for a global regulator involved in the activation of Vvp (Elgaml and Miyoshi, 2017); and the overtranscription of several quorum sensing-related regulators that activate the expression of *vvp* (Kim et al., 2013; Elgaml and Miyoshi, 2017). Similar results were reported in *Photobacterium damselae* subsp. *piscicida* (*Pdp*) and *V. harveyi* (Matanza and Osorio, 2018; Montánchez et al., 2019), two multi-host pathogens like *V. vulnificus*, which suggests a general role of temperature on bacterial colonization in Vibrionaceae. Our model in Figure 7 also shows that an environment rich in free iron would also contribute to activate a motile and chemotactic phenotype. Thus, iron and temperature could act synergistically facilitating the first step in host colonization in the environment.

## Attachment, Multiplication and Biofilm Formation

Once attracted to the epithelium, *V. vulnificus* has to attach and multiply to colonize successfully the host tissues. In case of eels, *V. vulnificus* attaches to the gill epithelium, multiplies on it and forms biofilms (Valiente et al., 2008b). With the exception of the flagellum, temperature did not seem to control the expression of any of the attachment appendages described in the species, although all of them are controlled by iron levels (Pajuelo et al., 2016). In this sense, the flagellum could contribute to host tissue attachment, and this initial adhesion could be enhanced by an increase in temperature. Regarding bacterial multiplication, temperature clearly influenced the entrance of *V. vulnificus* in the log phase of growth, which was very slow at 20°C, medium and 25°C and fast at 28 and 37°C; while did not affect the maximum population size, which was similar at all the assayed temperatures. Therefore, once *V. vulnificus* attached to the host tissues it could be easily eliminated by the local immunity before achieving the population size needed to produce fish vibriosis when fish are kept bellow 20°C, while it could multiply and cause a local inflammation that would facilitate invasion of the blood stream (Callol et al., 2015) when fish are kept at 28°C. In case of humans, the bacterial survival in a wound could also be favored at temperatures above 25°C.

When we analyzed the global transcriptome from cells obtained in the mid-log phase of growth, we found that most of the DEGs in response to temperature were involved in metabolism and transport of nutrients. Thus, an increase in temperature upregulated genes related to amino acids, lipids, fatty acids, nucleoside and iron metabolism and ferrous/ferric iron- and vulnibactin dependent transport, most of these nutrient being present in the blood and mucosae that surrounds fish tissue (Reverter et al., 2018; Chernyavskikh et al., 2019). Similar results were previously reported in other gram negative species such as *Pdp* and *Pseudomonas putida* (Fonseca et al., 2011; Matanza and Osorio, 2018) including *V. vulnificus* (Kim et al., 2016). In agreement, *crp*, a positive regulator for iron uptake (Kim et al., 2016), was also upregulated at 28 and 37°C. Remarkably, and contrarily to previous data in other Vibrionaceae species (Liu et al., 2017; Matanza and Osorio, 2018), oligopeptide uptake and metabolism (*opp* operon) was also activated by temperature suggesting a putative role of this kind of metabolism in bacterial fitness in serum, a medium particularly rich in oligopeptides (Samant et al., 2008).

In regard to biofilm, temperature did not affect its formation, unless in the conditions assayed. In fact, some studies reported that this process is controlled by iron, among other external factors (Phippen and Oliver, 2015; Pajuelo et al., 2016; Chodur et al., 2018; Chodur and Rowe-Magnus, 2018) (Figure 7).

## Resistance to Innate Defenses

*V. vulnificus* needs to overcome host innate defenses in mucus and blood to cause septicemia. We found evidence that temperature enhances bacterial ability to cope with stress caused by the host innate immunity as an upregulation of genes involved in membrane regeneration, and resistance to the oxidative and nitrosative stress were found upregulated with an increase in temperature. We tested resistance to oxidative stress and microcidal peptides (colystin and lysozime) and we only confirmed this relationship for microcidal peptides resistance (data not shown). Therefore, temperature could indirectly enhance *V. vulnificus* virulence, preparing the bacterium for resisting the microcidal peptides, ROS and/or NO produced by host phagocytes and neutrophils (Kaufmann and Dorhoi, 2016; Uribe-Querol and Rosales, 2017). These processes are also controlled by iron although the genes involved are not the same (Pajuelo et al., 2016). In previous studies, we demonstrated that iron also controls the production of a protective envelope, either rich in capsule in iron-overloaded human blood or rich in HMW/MMW *O*-antigen portion of LPS together with two plasmid IROMPs in eel blood (Pajuelo et al., 2016; Hernández-Cabanyero et al., 2019). These two IROMPs constitute a previously described protein kit for survival in fish blood that confers the bacterium the specific ability to resist to fish complement and phagocytosis (Hernández-Cabanyero et al., 2019). We found transcriptomic and phenotypic evidence that temperature could also control the LPS production; in particular, the MMW *O*-antigen production, as well as the protein of the kit that acts as a specific receptor for fish transferrin (Ftbp), and, therefore it could act synergistically with iron contributing to the highly resistant phenotype to fish innate immunity in the blood.

## Sepsis

Notably, in our study none of the *V. vulnificus* major toxins or their regulators were controlled by temperature. These toxins act synergistically *in vivo* being RtxA1 the toxin responsible for death by sepsis in both mice and eels (Lee et al., 2013; Callol et al., 2015). This result is in accordance with previous studies that suggest that virulence-related genes are controlled, directly or indirectly, by other external signals such as iron and/or presence of eukaryotic cells in the environment (Pajuelo et al., 2016; Murciano et al., 2017; Hernández-Cabanyero et al., 2019). In fact, we found in a previous study performed in serum that both toxins are subjected to a complex regulation in which iron is a relevant signal but not the only one (Hernández-Cabanyero et al., 2019). Considering the importance of the toxins RtxA1 and VvhA in *V. vulnificus* virulence, we expected to find them upregulated at 28 and 37°C. However, we did not find any of them differentially expressed at the assayed conditions (either by microarray, nor by RT-qPCR). We did not observe any phenotypic difference in hemolyses capability among temperatures either. These results are supported by previous studies in *Pdp* (Matanza and Osorio, 2018).

Finally, our results highlight that the external temperature is a major factor controlling pVvBt2 gene transcription, as most of the genes were maximally upregulated at 28°C. The plasmid encodes the protein kit for survival in fish blood together with a series of uncharacterized proteins probably involved in growth in sepsis (Lee et al., 2008; Pajuelo et al., 2015; Hernández-Cabanyero et al., 2019). In a similar way, Matanza and Osorio have recently described that most of the genes encoded by *Pdp* virulence plasmid are upregulated with an increase in temperature, including *ftbp*- and *fpcrp*-like genes (Matanza and Osorio, 2018). Our results only show *ftbp* but not *fpcrp* as genes regulated by temperature. This could be explained since in *V. vulnificus* these two genes are located in different operons, while in *Pdp* they belong to the same operon (results not shown). Finally, most of these genes also belonged to iron stimulon as they were upregulated under iron starvation, which stressed that temperature and iron could control synergistically the entire life cycle of the pathogen (Figure 7).

## Conclusion

Our results show that the capability of *V. vulnificus* to cause disease and its severity is multifactorial, revealing that the environmental temperature acts as an external signal that, together with other signals (i.e., iron), controls virulence and adaptation to host mechanisms in the pathogen *V. vulnificus*. Thus, environmental temperatures over 25°C activate motility, chemotaxis and protease production, shorten the lag phase, activate metabolism, iron uptake, and the production of a partially protective external envelope (at least against fish innate immunity), all these processes being involved in host colonization and fish septicemia. However, the main virulence factors for this pathogen, the toxin RtxA1 and the hemolysin VvhA, both involved in death by sepsis, are not controlled by temperature. According to these results, in iron rich environments, like those found in fish farms, and at infective temperatures, *V. vulnificus* would be motile, which in turn would result in attraction to mucus and colonization of the fish host. In the same way, in seawater the bacterium would increase motility with temperature (in summer, or warm months) being able to be attracted by the human blood and colonize wounds. From gills and human wounds, the pathogen would enter occasionally to the bloodstream where it should produce some of the factors involved in resistance to complement system and phagocytosis. From that point, other signals such as iron and contact with eukaryotic cells would trigger the formation of a truly protective barrier together with the expression of a toxic phenotype involved in septicemia. Although most of the genes belonging to temperature stimulon do not belong to iron stimulon, many of them are involved in the same processes. In this sense, both temperature and iron would act synergistically to trigger virulence and survival in and between hosts. Finally, our results confirm that beyond the effect of temperature on the distribution of *V. vulnificus* in the environment, there is a real effect on the infectious capacity of this species that must be taken into account to predict the real risk of *V. vulnificus* infection caused by global warming.

## Data Availability Statement

All datasets generated for this study are included in the article/Supplementary  Material.

## Author Contributions

CH-C, ES, and CA conceived and designed the study. CH-C, BF, DP, and FR-L performed the lab experiments. CH-C and EV-V analyzed the data. CH-C wrote the first draft of the manuscript. ES, BF, and CA corrected the draft. CH-C and CA built the final version of the manuscript. All authors contributed to manuscript revision, and read and approved the submitted version.

## Conflict of Interest

The authors declare that the research was conducted in the absence of any commercial or financial relationships that could be construed as a potential conflict of interest.

## Footnotes

1 https://www.epa.gov/climate-indicators/climate-change-indicators-sea-surface-temperature

2 http://bioinformatics.psb.ugent.be/webtools/Venn/

## Supplementary Material

FIGURE S1 Venn diagram for the number of DEGs found at each temperature in common with iron stimulon (previously described by Pajuelo et al., 2016) or with DEGs in host serum (previously described by Hernández-Cabanyero et al., 2019). **(A)** Number of DEGs in common between the three infective temperatures assayed (25, 28, and 37°C). **(B)** Number of DEGs at 25°C in common with iron stimulon and eel serum. **(C)** Number of DEGs at 28°C in common with iron stimulon and eel serum. **(D)** Number of DEGs at 37°C in common with iron stimulon and iron-overloaded human serum.

TABLE S1 Primers used for RT-qPCR analysis.

TABLE S2 Differentially expressed genes by *V. vulnificus* at 25°C vs. 20°C in CM9. The fold change value for each gene is indicated with also the inclusion of those previously described to be differentially expressed in iron stimulon, fur regulon and eel serum (Pajuelo et al., 2016; Hernández-Cabanyero et al., 2019). ^∗^: present in iron stimulon, fur regulon or eel-serum but with upside downregulation. ^∗∗^: only genes with values of fold change −2 ≤ X ≤ 2 with a *p*-value cut-off of 0.05 at 25°C vs. 20°C were considered. +: gene upregulated at 25°C; −: gene downregulated at 25°C.

TABLE S3 Differentially expressed genes by *V. vulnificus* at 28°C vs. 20°C in CM9. The fold change value for each gene is indicated with also the inclusion of those previously described to be differentially expressed in iron stimulon, fur regulon and eel serum (Pajuelo et al., 2016; Hernández-Cabanyero et al., 2019). ^∗^: present in iron stimulon, fur regulon or eel serum but with upside downregulation. ^∗∗^: only genes with values of fold change −2 ≤ X ≤ 2 with a *p*-value cut-off of 0.05 at 28°C vs. 20°C were considered. +: gene upregulated at 28°C; −: gene downregulated in at 28°C.

TABLE S4 Differentially expressed genes by *V. vulnificus* at 37°C vs. 20°C in CM9. The fold change value for each gene is indicated with also the inclusion of those previously described to be differentially expressed in iron stimulon, fur regulon and eel serum (Pajuelo et al., 2016; Hernández-Cabanyero et al., 2019). ^∗^: present in iron stimulon, fur regulon or eel serum but with upside downregulation. ^∗∗^: only genes with values of fold change −2 ≤ X ≤ 2 with a *p*-value cut-off of 0.05 at 37°C vs. 20°C were considered. +: gene upregulated at 37°C; -: gene downregulated at 37°C.

TABLE S5 *V. vulnificus* R99 strain temperature stimulon. The temperature stimulon consists on the set of DEGs by *V. vulnificus* in response to an increase in temperature. The value of fold change per gene at each infective temperature (25, 28, and 37°C vs. non-infective temperature [20°C]) is shown. ^∗^: only genes with values of fold change −2 ≤ X ≤ 2 with a *p*-value cut-off of 0.05 considered. +: gene upregulated at infective temperature; −: gene downregulated in at infective temperature. NS, non-significant.
