# Supplementary figures and images for: The Effect of the Environmental Temperature on the Adaptation to Host in the Zoonotic Pathogen Vibrio vulnificus
Source: Front Microbiol. 2020 Mar 27;11:489. doi: 10.3389/fmicb.2020.00489 (PMC7137831; doi:10.3389/fmicb.2020.00489)

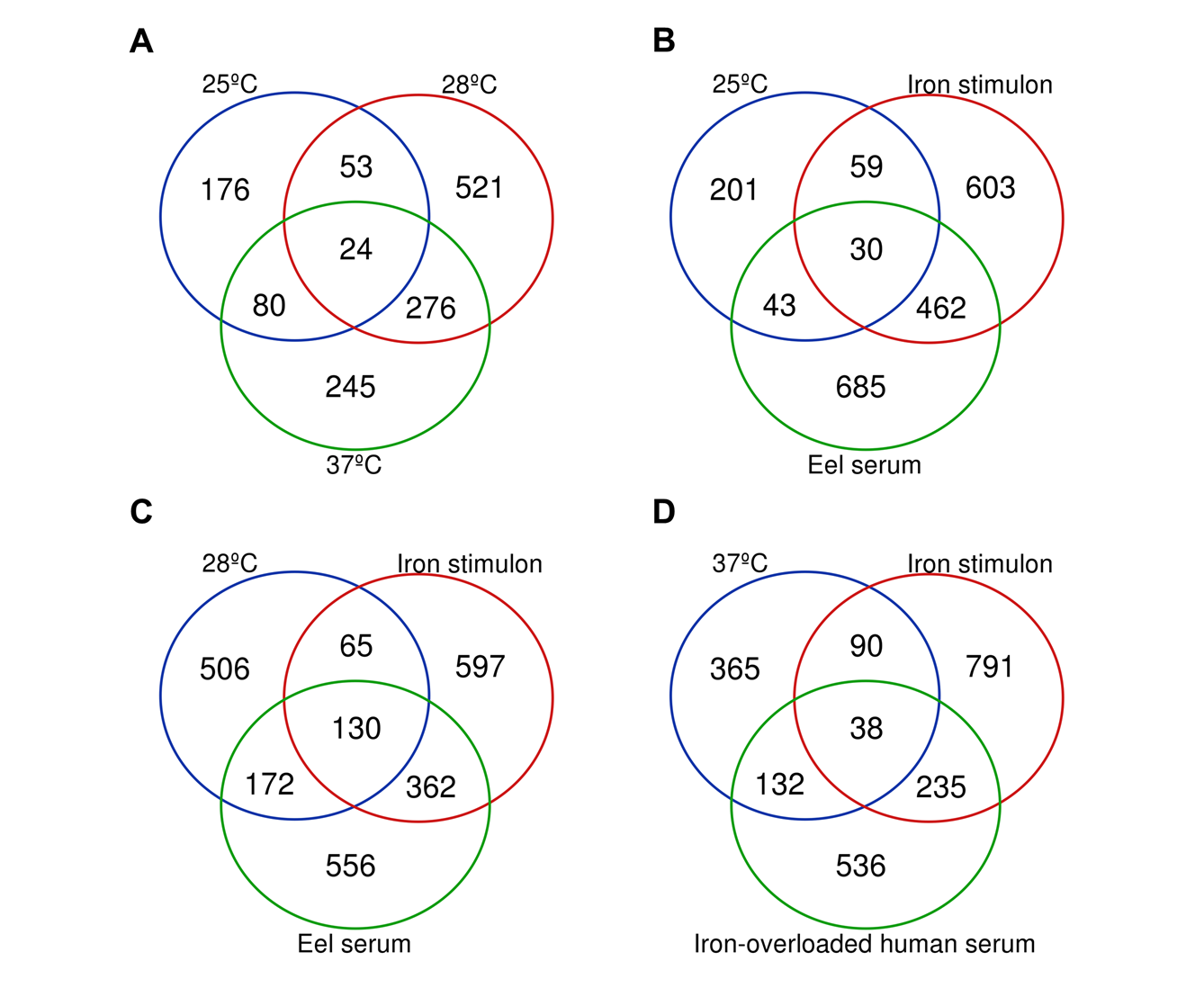

Supplement: FIGURE S1 — Venn diagram for the number of DEGs found at each temperature in common with iron stimulon (previously described by Pajuelo et al., 2016) or with DEGs in host serum (previously described by Hernández-Cabanyero et al., 2019). (A) Number of DEGs in common between the three infective temperatures assayed (25, 28, and 37°C). (B) Number of DEGs at 25°C in common with iron stimulon and eel serum. (C) Number of DEGs at 28°C in common with iron stimulon and eel serum. (D) Number of DEGs at 37°C in common with iron stimulon and iron-overloaded human serum. [file Image_1.TIF]
