# Supplementary material for: The Effect of the Environmental Temperature on the Adaptation to Host in the Zoonotic Pathogen Vibrio vulnificus
Source: Front Microbiol. 2020 Mar 27;11:489. doi: 10.3389/fmicb.2020.00489 (PMC7137831; doi:10.3389/fmicb.2020.00489)
Supplement: TABLE S1 — Primers used for RT-qPCR analysis. [file Data_Sheet_1.PDF]

Table S1. Primers used for RT-qPCR analysis.

Gene name	Gene acronym	Sequence
Recombinase A	*recA*	Fw: CGCCAAAGGCAGAAATCG<br>Rv: ACGAGCTTGAAGACCCATGTG
Fish transferrin binding protein	*ftbp*	Fw: CACTCGCCTCTTTGGTTTCG<br>Rv: GGGACTGATTCTCTCTTC
Capsular polysaccharide synthesis enzyme CpsA	*cpsA*	Fw: GCAGCTCATCGAGTGACGTA<br>Rv: GCAGCTCATCGAGTGACGTA
Flp pilus assembly protein	*flp*	Fw: TGGTGTTAGCCATAGGAACTCTCTT<br>Rv: CCACCTGCCTCTCCTTCCA
Maltose/maltodextrin ABC transporter, permease protein	*malG*	Fw: CCAGAATCCACGTCCAACGT<br>GAGTGCCGATGCGGATGT
Biofim transcriptional regulator	*vpsT*	Fw: GAAGGAAGAACCGCAGTTAGA<br>Rv: ATCGTCTCGGTGATAAA
Potassium uptake protein, integral membrane component	*ktrA*	Fw: ATCGGCGCAGACCACATC<br>Rv: TCGCCACGCGGATCA
MARTX	*rtxA1*$_3$	Fw: GAGTGATGATGGGCGCTTTAC<br>Rv: CAGCCGCGATGGATGCT
Haemolysin (vulnificolysin)	*vvhA*	Fw: TGTTTATGGTGAGAACGGTGACA<br>Rv: TTCTTTATCTAGGCCCCAAACTTG
Protease	*vvp*	Fw: TTGCCGCAGTCGTGTGTT<br>Rv: CGGAGACGGACACCATTTCT
