# Supplementary material for: The Effect of the Environmental Temperature on the Adaptation to Host in the Zoonotic Pathogen Vibrio vulnificus
Source: Front Microbiol. 2020 Mar 27;11:489. doi: 10.3389/fmicb.2020.00489 (PMC7137831; doi:10.3389/fmicb.2020.00489)
Supplement: TABLE S2 — Differentially expressed genes by V. vulnificus at 25°C vs. 20°C in CM9. The fold change value for each gene is indicated with also the inclusion of those previously described to be differentially expressed in iron stimulon, fur regulon and eel serum (Pajuelo et al., 2016; Hernández-Cabanyero et al., 2019). ∗: present in iron stimulon, fur regulon or eel-serum but with upside downregulation. ∗∗: only genes with values of fold change −2 ≤ X ≤ 2 with a p-value cut-off of 0.05 at 25°C vs. 20°C were considered. +: gene upregulated at 25°C; −: gene downregulated at 25°C. [file Data_Sheet_2.PDF]

Table S2. Differentially expressed genes by *V. vulnificus* at 25ºC vs 20ºC in CM9. The fold change value for each gene is indicated with also the inclusion of those previously described to be differentially expressed in iron stimulon, fur regulon and eel serum (Pajuelo et al., 2016; Hernández-Cabanyero et al., 2019).
*: present in iron stimulon, fur regulon or eel-serum but with upside downregulation.
**: only genes with values of fold change -2≤X≤2 with a p-value cut-off of 0.05 at 25ºC vs 20ºC were considered. +: gene upregulated at 25ºC; -: gene downregulated at 25ºC.

Gene	Fold change	Iron stimulon	Fur regulon	Eel serum
sensor histidine kinase	17.63	YES	NO	YES
Enoyl-CoA hydratase (EC 4.2.1.17) / Delta(3)-cis-delta(2)-trans-enoyl-CoA isome	15.04	YES	NO	YES
Predicted deacylase	13.65	NO	NO	YES
Predicted ATP-dependent endonuclease of the OLD family	12.29	NO	NO	YES
2,4-dienoyl-CoA reductase [NADPH] (EC 1.3.1.34)	11.30	NO	NO	NO
Peptide ABC transporter, ATP-binding protein	10.38	NO	NO	YES
Methyl-accepting chemotaxis protein	8.53	NO	NO	NO
Branched-chain amino acid aminotransferase (EC 2.6.1.42)	7.78	YES	NO	NO
Sodium-dependent transporter	7.69	NO	NO	NO
Phosphate starvation-inducible ATPase PhoH with RNA binding motif	7.14	NO	NO	NO
hypothetical protein	7.03	YES	NO	NO
Predicted polymerase	6.38	NO	NO	NO
Anthranilate phosphoribosyltransferase *trpD*	6.08	NO	NO	NO
C4-dicarboxylate transport transcriptional regulatory protein	5.99	NO	NO	NO
3-ketoacyl-CoA thiolase (EC 2.3.1.16) @ Acetyl-CoA acetyltransferase (EC 2.3.1.	5.86	NO	NO	YES
Methyl-accepting chemotaxis protein I (serine chemoreceptor protein)	5.65	NO	NO	NO
CDP-diacylglycerol--serine O-phosphatidyltransferase (EC 2.7.8.8)	5.47	YES	NO	YES
Transglycosylase, Slt family	5.47	NO	NO	NO
hypothetical protein	5.32	NO	NO	NO
Transporter, putative	5.26	NO	NO	NO
Exodeoxyribonuclease I (EC 3.1.11.1)	5.12	NO	NO	NO
Ornithine carbamoyltransferase (EC 2.1.3.3)	5.01	NO	NO	NO
D-3-phosphoglycerate dehydrogenase (EC 1.1.1.95)	4.98	NO	NO	YES
hypothetical protein	4.94	YES	NO	NO
TRAP transporter solute receptor, unknown substrate 6	4.80	NO	NO	NO

GGDEF and EAL domain proteins	4.78	YES	NO	NO
Alpha-amylase (EC 3.2.1.1)	4.65	NO	NO	NO
Anaerobic glycerol-3-phosphate dehydrogenase subunit B (EC 1.1.5.3)	4.61	NO	NO	YES
Formate dehydrogenase subunit or accessory protein	4.36	NO	NO	NO
FIGfam010717	4.30	YES	NO	NO
Protoporphyrinogen IX oxidase, oxygen-independent, HemG (EC 1.3.-.-)	4.28	NO	NO	NO
Di-and tricarboxylate transporter	4.19	NO	NO	YES
Deoxyribodipyrimidine photolyase (EC 4.1.99.3)	4.18	NO	NO	NO
Multidrug resistance transporter, Bcr/CflA family	4.13	NO	NO	YES
Allophanate hydrolase 2 subunit 1 (EC 3.5.1.54)	4.11	NO	NO	NO
Glyoxylase family protein	4.06	NO	NO	NO
Periplasmic alpha-amylase (EC 3.2.1.1)	4.02	NO	NO	NO
Predicted permease	4.01	NO	NO	NO
Outer membrane protein Imp, required for envelope biogenesis / Organic solvent	3.98	YES	NO	NO
Indole-3-glycerol phosphate synthase (EC 4.1.1.48) / Phosphoribosylanthranilate	3.97	NO	NO	NO
Dipeptide-binding ABC transporter, periplasmic substrate-binding component (TC	3.87	NO	NO	NO
Cystathionine beta-lyase (EC 4.4.1.8)	3.77	NO	NO	NO
Arginine deiminase (EC 3.5.3.6)	3.74	YES	NO	NO
putative Glutathione-regulated potassium-efflux system protein KefB	3.68	NO	NO	NO
Ferric iron ABC transporter, ATP-binding protein	3.66	NO	NO	YES
Phosphosugar mutase of unknown sugar (see annotation)	3.64	NO	NO	YES
hypothetical protein	3.60	YES	NO	NO
COG1720: Uncharacterized conserved protein	3.53	NO	NO	NO
Ribosomal-protein-S5p-alanine acetyltransferase	3.48	YES	NO	NO
Cysteine synthase B (EC 2.5.1.47)	3.42	NO	NO	YES
Acetolactate synthase large subunit (EC 2.2.1.6)	3.41	NO	NO	NO
Glutathione-regulated potassium-efflux system ancillary protein KefG	3.35	YES	NO	NO
hypothetical protein	3.34	NO	NO	NO
3-hydroxydecanoyl-[ACP] dehydratase (EC 4.2.1.60)	3.33	NO	NO	NO
Long-chain fatty acid transport protein	3.31	NO	NO	NO
Predicted metal-dependent hydrolase with the TIM-barrel fold	3.21	NO	NO	NO
DNA helicase IV	3.21	NO	NO	NO

Predicted signal transduction protein	3.20	NO	NO	YES
hypothetical protein	3.18	NO	NO	NO
Response regulator	3.12	YES	NO	YES
2-dehydropantoate 2-reductase (EC 1.1.1.169)	3.11	NO	NO	NO
hypothetical protein	3.11	NO	NO	NO
Membrane-bound lytic murein transglycosylase D precursor (EC 3.2.1.-)	3.11	NO	NO	NO
Nitrogen regulation protein NR(I)	3.06	YES	NO	NO
Predicted L-lactate dehydrogenase, Fe-S oxidoreductase subunit YkgE	3.05	YES	NO	YES
Cytochrome c-type biogenesis protein DsbD, protein-disulfide reductase (EC 1.8.	3.02	NO	NO	YES
S-(hydroxymethyl)glutathione dehydrogenase (EC 1.1.1.284)	3.02	NO	NO	NO
Predicted membrane-associated metal-dependent hydrolase	3.01	NO	NO	NO
Cell division protein FtsK	3.01	YES	NO	YES
diguanylate cyclase (GGDEF domain) with PAS/PAC sensor	3.00	NO	NO	YES
Vulnibactin utilization protein VuuB	2.99	YES	NO	NO
FIG139976: hypothetical protein	2.98	YES	NO	NO
Glutamate synthase [NADPH] small chain (EC 1.4.1.13)	2.98	YES	NO	NO
DinG family ATP-dependent helicase YoaA	2.96	YES	NO	NO
membrane protein	2.96	NO	NO	NO
ABC-type multidrug transport system, ATPase component	2.94	NO	NO	NO
ABC-type protease exporter, membrane fusion protein (MFP) family component PrtE	2.92	YES	NO	NO
hypothetical protein	2.89	NO	NO	NO
oxidoreductase, short-chain dehydrogenase/reductase family	2.85	NO	NO	NO
Pyruvate kinase (EC 2.7.1.40)	2.84	NO	NO	YES
Catalase (EC 1.11.1.6) / Peroxidase (EC 1.11.1.7)	2.83	YES*	NO	NO
Methyl-accepting chemotaxis protein	2.82	YES	NO	YES
Alkyl hydroperoxide reductase protein F (EC 1.6.4.-)	2.82	NO	NO	NO
Predicted P-loop ATPase fused to an acetyltransferase COG1444	2.82	YES	NO	NO
Transport ATP-binding protein CydD	2.81	NO	NO	YES
Dephospho-CoA kinase (EC 2.7.1.24)	2.81	YES	NO	NO
Pole remodelling regulatory diguanylate cyclase	2.79	NO	NO	NO
Predicted endonuclease distantly related to archaeal Holliday junction resolvas	2.75	NO	NO	NO
Aldehyde dehydrogenase (EC 1.2.1.3); Probable coniferyl aldehyde dehydrogenase	2.75	NO	NO	NO
Glutamate-ammonia-ligase	2.73	NO	NO	NO

adenylyltransferase (EC 2.7.7.42)				
ABC-type dipeptide transport system, periplasmic component	2.72	NO	NO	NO
Putative oxidoreductase YncB	2.72	NO	NO	NO
MSHA biogenesis protein MshH	2.72	NO	NO	NO
Deoxyguanosinetriphosphate triphosphohydrolase (EC 3.1.5.1)	2.72	YES	NO	NO
Histidinol dehydrogenase (EC 1.1.1.23)	2.71	NO	NO	NO
Glutathione S-transferase (EC 2.5.1.18)	2.71	NO	NO	NO
Hydrolase, alpha/beta fold family functionally coupled to Phosphoribulokinase	2.69	NO	NO	NO
Lipopolysaccharide heptosyltransferase I (EC 2.4.1.-)	2.68	NO	NO	NO
Molybdopterin-guanine dinucleotide biosynthesis protein MobB / Molybdopterin bi	2.68	NO	NO	NO
ABC-type antimicrobial peptide transport system, permease component	2.67	NO	NO	NO
Universal stress protein A	2.67	NO	NO	NO
Membrane fusion component of tripartite multidrug resistance system	2.66	YES	NO	NO
Autoinducer 2-binding periplasmic protein LuxP precursor	2.65	YES	NO	NO
hypothetical protein	2.64	YES	NO	NO
Conserved protein YcjX with nucleoside triphosphate hydrolase domain	2.64	YES	NO	NO
Periplasmic nitrate reductase precursor (EC 1.7.99.4)	2.64	YES	NO	YES
Na+/H+ antiporter, putative	2.60	YES	NO	NO
Endonuclease III (EC 4.2.99.18)	2.58	NO	NO	NO
Transcriptional regulator, TetR family	2.57	NO	NO	YES*
Menaquinone-specific isochorismate synthase (EC 5.4.4.2)	2.57	NO	NO	NO
Succinylglutamic semialdehyde dehydrogenase (EC 1.2.1.71)	2.55	NO	NO	NO
RNA polymerase sigma factor RpoS	2.55	NO	NO	NO
DNA mismatch repair protein MutS	2.52	YES	NO	NO
Uncharacterized protein YtfM precursor	2.52	NO	NO	NO
Acetolactate synthase small subunit (EC 2.2.1.6)	2.51	NO	NO	NO
Protein ydjA	2.50	YES	NO	NO
Aspartate aminotransferase (AspB-4) (EC 2.6.1.1)	2.49	YES*	NO	YES*
PQQ-dependent oxidoreductase, gdhB family	2.48	NO	NO	NO
Transcriptional regulator, LysR family	2.46	YES	NO	NO
Lipoprotein releasing system ATP-binding protein LolD	2.45	NO	NO	NO
3-oxoacyl-[ACP] reductase (EC 1.1.1.100)	2.44	YES	NO	NO

LppC putative lipoprotein	2.43	YES	NO	NO
Flagellar regulatory protein FleQ	2.43	NO	NO	NO
Arylesterase precursor (EC 3.1.1.2)	2.41	NO	NO	NO
ABC-type dipeptide transport system, periplasmic component	2.41	NO	NO	NO
hypothetical protein	2.40	NO	NO	NO
C4-dicarboxylate transporter	2.40	NO	NO	NO
Methyl-accepting chemotaxis protein II (mcp-II) (aspartate chemoreceptor protei	2.39	NO	NO	NO
hypothetical protein	2.39	YES	NO	NO
Predicted transcriptional regulator of pyridoxine metabolism	2.38	NO	NO	NO
FIG023406: hypothetical protein	2.37	NO	NO	NO
UDP-N-acetylmuramoylalanine--D-glutamate ligase (EC 6.3.2.9)	2.36	YES	NO	NO
NAD-dependent malic enzyme (EC 1.1.1.38)	2.36	NO	NO	NO
DNA-directed RNA polymerase specialized sigma subunit	2.36	NO	NO	NO
Chaperone protein HscA	2.36	NO	NO	NO
hypothetical protein	2.36	NO	NO	YES*
GNAT family acetyltransferase VC2332	2.36	NO	NO	NO
Signal transduction histidine kinase	2.36	NO	NO	NO
Nitrate reductase cytochrome c550-type subunit	2.36	YES	NO	YES
Poly(A) polymerase (EC 2.7.7.19)	2.36	NO	NO	NO
Putative protein-S-isoprenylcysteine methyltransferase	2.34	NO	NO	NO
5-methylaminomethyl-2-thiouridine-forming enzyme mnmC	2.34	NO	NO	NO
Formamidopyrimidine-DNA glycosylase (EC 3.2.2.23)	2.34	NO	NO	NO
C4-dicarboxylate transport transcriptional regulatory protein	2.34	NO	NO	YES
Isochorismatase (EC 3.3.2.1) of siderophore biosynthesis	2.33	YES	NO	YES
Diaminopimelate epimerase (EC 5.1.1.7)	2.33	NO	NO	NO
3'-to-5' exoribonuclease RNase R	2.33	NO	NO	NO
L-serine dehydratase (EC 4.3.1.17)	2.32	NO	NO	YES*
Putative regulator protein	2.32	YES	NO	NO
RND efflux system, outer membrane lipoprotein CmeC	2.32	NO	NO	NO
Protein ThiJ	2.32	NO	NO	NO
Fimbrial protein pilin	2.31	NO	NO	NO
5-nucleotidase SurE (EC 3.1.3.5)	2.31	YES	NO	NO
Cystathionine gamma-synthase (EC 2.5.1.48)	2.31	NO	NO	NO
Amino acid ABC transporter, periplasmic amino acid-binding protein	2.30	YES*	NO	NO
Superoxide dismutase [Cu-Zn] precursor	2.30	NO	NO	NO

(EC 1.15.1.1)				
tRNA pseudouridine 13 synthase (EC 4.2.1.-)	2.29	NO	NO	NO
FIG027190: Putative transmembrane protein	2.28	NO	NO	NO
tRNA uridine 5-carboxymethylaminomethyl modification enzyme GidA	2.26	NO	NO	NO
Chromate transport protein ChrA	2.26	NO	NO	NO
Galactose operon repressor, GalR-LacI family of transcriptional regulators	2.26	NO	NO	NO
Dihydrofolate synthase (EC 6.3.2.12) / Folylpolyglutamate synthase (EC 6.3.2.17	2.25	NO	NO	NO
Nucleoside-diphosphate-sugar epimerase	2.25	YES	NO	NO
Flagellar hook-associated protein FlgL	2.25	YES*	NO	YES
hypothetical protein	2.24	NO	NO	NO
GMP reductase (EC 1.7.1.7)	2.24	NO	NO	YES
Transcriptional regulator, LysR family	2.22	NO	NO	NO
hypothetical protein	2.22	NO	NO	NO
[Protein-PII] uridylyltransferase (EC 2.7.7.59)	2.22	NO	NO	NO
General secretion pathway protein D	2.21	YES	NO	NO
Anaerobic glycerol-3-phosphate dehydrogenase subunit C (EC 1.1.5.3)	2.21	NO	NO	YES
Protein-L-isoaspartate O-methyltransferase (EC 2.1.1.77)	2.21	YES	NO	NO
Regulator of sigma D	2.21	NO	NO	NO
hypothetical protein	2.20	NO	NO	YES*
Transcriptional regulator, LysR family	2.20	NO	NO	NO
Phosphoglycerol transferase I (EC 2.7.8.20)	2.19	YES	NO	NO
Oligopeptidase A (EC 3.4.24.70)	2.19	NO	NO	NO
Predicted D-lactate dehydrogenase, Fe-S protein, FAD/FMN-containing	2.19	NO	NO	NO
Spermidine Putrescine ABC transporter permease component PotB (TC 3.A.1.11.1)	2.19	NO	NO	NO
Phosphate:acyl-ACP acyltransferase PlsX	2.18	NO	NO	YES
Flagellar sensor histidine kinase FleS	2.18	NO	NO	NO
Flp pilus assembly protein TadD, contains TPR repeat	2.17	YES	NO	NO
hypothetical protein	2.17	NO	NO	NO
nonspecific acid phosphatase precursor	2.16	NO	NO	NO
Multicopper oxidase	2.16	YES	NO	YES
hypothetical protein	2.16	NO	NO	NO
PrpF protein involved in 2-methylcitrate cycle	2.16	NO	NO	NO
Transcriptional regulator	2.16	NO	NO	NO
Argininosuccinate synthase (EC 6.3.4.5)	2.15	NO	NO	NO
Uncharacterized iron-regulated protein	2.14	YES	NO	YES
DnaK-related protein	2.14	NO	NO	NO
Exported zinc metalloprotease YfgC	2.13	YES	NO	NO

precursor				
hypothetical protein	2.13	YES	NO	YES
Lipoprotein releasing system transmembrane protein LolC	2.12	NO	NO	NO
Hypothetical Transcriptional Regulator	2.12	NO	NO	NO
Beta-galactosidase (EC 3.2.1.23) / Beta-glucosidase/6-phospho-beta-glucosidase	2.12	NO	NO	NO
Sugar binding protein of sugar ABC transporter	2.11	NO	NO	NO
Flagellar hook-associated protein FlgK	2.11	YES*	NO	YES
Nitrate ABC transporter, ATP-binding protein	2.10	NO	NO	NO
Flagellar hook-associated protein FlgK	2.10	NO	NO	NO
SgrR, sugar-phosphate stress, transcriptional activator of SgrS small RNA	2.10	YES	NO	NO
HTH-type transcriptional regulator IlvY	2.10	NO	NO	YES
General secretion pathway protein D	2.09	YES*	NO	YES*
putative; ORF located using Glimmer/Genemark	2.09	NO	NO	NO
ABC-type multidrug transport system, permease component	2.09	NO	NO	NO
Adenosylcobinamide-phosphate guanylyltransferase (EC 2.7.7.62)	2.09	NO	NO	NO
Aspartokinase (EC 2.7.2.4) / Homoserine dehydrogenase (EC 1.1.1.3)	2.08	NO	NO	NO
DNA polymerase III alpha subunit (EC 2.7.7.7)	2.08	NO	NO	NO
Lipoprotein NlpD	2.08	YES*	NO	YES*
hypothetical protein	2.07	NO	NO	NO
Heat shock protein HtpX / FIG017973: domain of unknown function	2.06	YES	NO	NO
Putative aminotransferase in phosphonate-related cluster	2.06	NO	NO	NO
Polyribonucleotide nucleotidyltransferase (EC 2.7.7.8)	2.06	NO	NO	NO
hypothetical protein	2.06	NO	NO	NO
hypothetical protein	2.06	NO	NO	NO
hypothetical protein	2.06	NO	NO	NO
hypothetical protein	2.06	NO	NO	NO
hypothetical protein	2.06	NO	NO	NO
Inosose isomerase (EC 5.3.99.-)	2.06	NO	NO	NO
L-asparaginase (EC 3.5.1.1)	2.06	NO	NO	NO
PTS system, N-acetylgalactosamine-specific IID component (EC 2.7.1.69)	2.06	NO	NO	NO
PTS system, N-acetylgalactosamine-specific IIB component (EC 2.7.1.69)	2.06	NO	NO	NO
Pyrrolidone-carboxylate peptidase (EC 3.4.19.3)	2.06	NO	NO	NO
RNA polymerase sigma factor RpoD	2.06	NO	NO	NO
Heavy metal sensor histidine kinase	2.06	NO	NO	NO

BatA (Bacteroides aerotolerance operon)	2.06	NO	NO	NO
Glycogen synthase, ADP-glucose transglucosylase (EC 2.4.1.21)	2.06	NO	NO	NO
Arginyl-tRNA synthetase (EC 6.1.1.19)	2.06	NO	NO	NO
2-aminoethylphosphonate ABC transporter periplasmic binding component (TC 3.A.1	2.06	NO	NO	NO
Nitrate ABC transporter, permease protein	2.06	NO	NO	NO
Tricarboxylate transport protein TctB	2.06	NO	NO	NO
hypothetical protein	2.05	YES	NO	NO
Flagellar M-ring protein FliF	2.05	NO	NO	NO
L-aspartate oxidase (EC 1.4.3.16)	2.05	NO	NO	NO
Flp pilus assembly protein TadB	2.05	NO	NO	NO
Hydroxymethylglutaryl-CoA reductase (EC 1.1.1.34)	2.04	YES	NO	YES
Glycerol-3-phosphate acyltransferase (EC 2.3.1.15)	2.03	YES	NO	NO
Sugar diacid utilization regulator SdaR	2.03	NO	NO	NO
Flavohemoprotein (Hemoglobin-like protein) (Flavohemoglobin) (Nitric oxide diox	2.03	YES	NO	NO
Signal transduction histidine kinase	2.03	YES	NO	NO
TonB-dependent receptor	2.03	NO	NO	NO
Glycerophosphoryl diester phosphodiesterase (EC 3.1.4.46)	2.02	NO	NO	YES
DNA-binding response regulator, LuxR family	2.02	NO	NO	YES
Permease of the drug/metabolite transporter (DMT) superfamily	2.01	YES	NO	YES
Transcriptional regulator, AraC family	2.01	NO	NO	NO
DNA polymerase III subunits gamma and tau (EC 2.7.7.7)	2.01	NO	NO	NO
Ferric siderophore transport system, periplasmic binding protein TonB	2.01	NO	NO	YES
Anhydro-N-acetylmuramic acid kinase (EC 2.7.1.-)	2.00	NO	NO	NO
Chromosomal replication initiator protein DnaA	2.00	NO	NO	NO
ftbp	2.00	YES	YES	YES
Acetyltransferase	-2.01	NO	NO	NO
hypothetical protein	-2.01	YES*	NO	NO
Preprotein translocase subunit SecG (TC 3.A.5.1.1)	-2.02	NO	NO	NO
Tellurite resistance protein	-2.02	NO	NO	NO
hypothetical protein	-2.03	YES	NO	YES
hypothetical protein	-2.05	YES*	NO	NO
ABC-type multidrug transport system, ATPase and permease component	-2.05	NO	NO	YES*
3-dehydroquinate dehydratase II (EC 4.2.1.10)	-2.06	NO	NO	NO
Protein yciN	-2.06	NO	NO	NO

LSU ribosomal protein L27p	-2.07	NO	NO	NO
hypothetical protein	-2.07	NO	NO	YES*
Pyruvate/2-oxoglutarate dehydrogenase complex, dihydrolipoamide dehydrogenase c	-2.08	YES*	NO	YES*
Stringent starvation protein B	-2.09	NO	NO	NO
hypothetical protein	-2.09	NO	NO	NO
Permease of the drug/metabolite transporter (DMT) superfamily	-2.09	NO	NO	NO
Molybdenum cofactor biosynthesis protein MoaA	-2.10	YES*	NO	YES*
tRNA-guanine transglycosylase (EC 2.4.2.29)	-2.12	NO	NO	NO
ATPase involved in DNA repair	-2.12	NO	NO	YES*
Ribosomal protein L11 methyltransferase (EC 2.1.1.-)	-2.12	NO	NO	NO
Nucleoid-associated protein NdpA	-2.15	NO	NO	YES*
Uncharacterized conserved protein	-2.18	NO	NO	NO
Heat shock protein HslJ	-2.20	NO	NO	NO
Transcription antitermination protein NusG	-2.22	YES	NO	NO
SSU ribosomal protein S20p	-2.23	YES*	NO	NO
Predicted amidohydrolase	-2.23	NO	NO	NO
Stringent starvation protein A	-2.24	YES	NO	NO
Protein-export membrane protein SecD (TC 3.A.5.1.1)	-2.25	NO	NO	NO
Alkylphosphonate utilization operon protein PhnA	-2.25	NO	NO	NO
hypothetical protein	-2.28	NO	NO	NO
Thioredoxin	-2.29	YES*	NO	NO
SH3 domain protein	-2.30	NO	NO	YES*
Acetyltransferase	-2.30	NO	NO	NO
hypothetical protein	-2.33	NO	NO	NO
Probable type IV pilus assembly FimV-related transmembrane protein	-2.36	NO	NO	YES*
hypothetical protein	-2.36	NO	NO	NO
LSU m5C1962 methyltransferase RlmI	-2.36	NO	NO	NO
hypothetical protein	-2.37	NO	NO	NO
Transcription termination protein NusB	-2.39	YES*	NO	YES*
Aerobic respiration control protein arcA	-2.39	NO	NO	YES*
Adenosine deaminase (EC 3.5.4.4)	-2.41	NO	NO	NO
hypothetical protein	-2.41	NO	NO	NO
Lipoprotein nlpI precursor	-2.42	NO	NO	NO
Quinolinate phosphoribosyltransferase [decarboxylating] (EC 2.4.2.19)	-2.46	NO	NO	NO
Vitamin B12 ABC transporter, permease component BtuC	-2.47	YES*	NO	NO
ATP-dependent RNA helicase DbpA	-2.47	NO	NO	YES*
DNA-binding protein Fis	-2.48	NO	NO	NO

Ribose ABC transport system, high affinity permease RbsD (TC 3.A.1.2.1)	-2.48	NO	NO	YES*
Mutator mutT protein (7,8-dihydro-8-oxoguanine-triphosphatase) (EC 3.6.1.-)	-2.50	NO	NO	NO
ATP-dependent RNA helicase VC1407	-2.50	NO	NO	NO
COG0398: uncharacterized membrane protein	-2.54	NO	NO	YES*
Ribosomal RNA large subunit methyltransferase N (EC 2.1.1.-)	-2.56	NO	NO	YES*
Preprotein translocase subunit SecE (TC 3.A.5.1.1)	-2.60	YES	NO	NO
putative transport system permease protein	-2.61	NO	NO	NO
Fructose-1,6-bisphosphatase, GlpX type (EC 3.1.3.11)	-2.63	YES	NO	NO
Purine nucleotide synthesis repressor	-2.65	YES	NO	NO
Short chain fatty acids transporter	-2.65	NO	NO	NO
Probable GTPase related to EngC	-2.74	YES	NO	YES
Ribonuclease E inhibitor RraB	-2.76	NO	NO	NO
Manganese-dependent inorganic pyrophosphatase (EC 3.6.1.1)	-2.77	NO	NO	NO
Putative protease	-2.80	NO	NO	NO
Histone acetyltransferase HPA2	-2.80	NO	NO	NO
Arylsulfatase (EC 3.1.6.1)	-2.82	NO	NO	NO
Preprotein translocase subunit YajC (TC 3.A.5.1.1)	-2.85	NO	NO	NO
hypothetical protein	-2.85	NO	NO	NO
Ribonucleotide reductase of class III (anaerobic), large subunit (EC 1.17.4.2)	-2.90	NO	NO	NO
Maltose/maltodextrin ABC transporter, permease protein MalG	-2.91	NO	NO	NO
hypothetical protein	-2.97	YES*	NO	NO
YaeQ protein	-2.98	NO	NO	YES*
Regulator of nucleoside diphosphate kinase	-3.08	NO	NO	NO
YrdC/Sua5 family protein, required for threonylcarbamoyladenosine (t(6)A) forma	-3.09	YES*	NO	NO
Ribose-phosphate pyrophosphokinase (EC 2.7.6.1)	-3.11	NO	NO	NO
Uncharacterized protein conserved in bacteria	-3.12	NO	NO	NO
hypothetical protein	-3.14	NO	NO	NO
Aspartokinase (EC 2.7.2.4)	-3.22	YES	NO	YES
Putative membrane protein	-3.36	NO	NO	NO
Adenylate kinase (EC 2.7.4.3)	-4.00	YES	NO	YES
Permease of the major facilitator superfamily	-4.00	NO	NO	YES*
hypothetical protein	-4.06	NO	NO	NO
Queuosine biosynthesis QueD, PTPS-I	-4.09	NO	NO	YES*
Uncharacterized protein conserved in bacteria	-4.32	NO	NO	YES
SSU ribosomal protein S21p	-4.38	YES	NO	YES

Regulator of competence-specific genes	-4.53	YES	NO	YES*
Guanylate kinase (EC 2.7.4.8)	-5.70	NO	NO	NO
