# Supplementary material for: The Effect of the Environmental Temperature on the Adaptation to Host in the Zoonotic Pathogen Vibrio vulnificus
Source: Front Microbiol. 2020 Mar 27;11:489. doi: 10.3389/fmicb.2020.00489 (PMC7137831; doi:10.3389/fmicb.2020.00489)
Supplement: TABLE S3 — Differentially expressed genes by V. vulnificus at 28°C vs. 20°C in CM9. The fold change value for each gene is indicated with also the inclusion of those previously described to be differentially expressed in iron stimulon, fur regulon and eel serum (Pajuelo et al., 2016; Hernández-Cabanyero et al., 2019). ∗: present in iron stimulon, fur regulon or eel serum but with upside downregulation. ∗∗: only genes with values of fold change −2 ≤ X ≤ 2 with a p-value cut-off of 0.05 at 28°C vs. 20°C were considered. +: gene upregulated at 28°C; −: gene downregulated in at 28°C. [file Data_Sheet_3.PDF]

Table S3. Differentially expressed genes by *V. vulnificus* at 28ºC vs 20ºC in CM9. The fold change value for each gene is indicated with also the inclusion of those previously described to be differentially expressed in iron stimulon, fur regulon and eel serum (Pajuelo et al., 2016; Hernández-Cabanyero et al., 2019).
*: present in iron stimulon, fur regulon or eel serum but with upside downregulation.
**: only genes with values of fold change -2≤X≤2 with a p-value cut-off of 0.05 at 28ºC vs 20ºC were considered. +: gene upregulated at 28ºC; -: gene downregulated in at 28ºC.

Gene	Fold change**	Iron stimulon	Fur regulon	Eel serum
Unknown, probable transcriptional regulator	46.12	YES*	NO	YES*
RNA polymerase sigma-54 factor RpoN	27.13	YES*	NO	YES*
Periplasmic nitrate reductase component NapE	22.49	YES*	NO	YES*
Chitinase (EC 3.2.1.14)	22.30	NO	NO	NO
FIG026291: Hypothetical periplasmic protein	21.45	NO	NO	YES*
Transcriptional regulator, ArsR family	19.88	YES*	NO	YES*
Transcriptional regulator, AraC family	18.56	YES*	NO	YES*
hypothetical protein	16.36	NO	NO	YES*
Transposase and inactivated derivatives	15.95	NO	NO	NO
Transcriptional regulator	15.46	YES*	NO	YES*
hypothetical protein	15.45	YES*	NO	YES*
hypothetical protein	15.42	YES	NO	NO
FIG002577: Putative lipoprotein precursor	14.70	NO	NO	YES*
pR99_vep40	14.21	NO	YES*	NO
Flp pilus assembly protein TadB	14.06	NO	NO	NO
Flp pilus assembly protein	14.02	YES*	NO	NO
Uncharacterized protein, similar to the N-terminal domain of Lon protease	12.91	NO	NO	YES*
hypothetical protein	12.83	NO	NO	NO
Methyl-accepting chemotaxis protein	12.77	YES*	NO	YES*
NAD-dependent glyceraldehyde-3-phosphate dehydrogenase (EC 1.2.1.12)	12.46	YES*	NO	YES*
Glutaredoxin 3	12.42	NO	NO	NO
hypothetical protein	12.22	NO	NO	YES*
Putative inner membrane protein	11.66	NO	NO	YES*
Flp pilus assembly protein	11.48	NO	NO	NO
Methionyl-tRNA formyltransferase (EC 2.1.2.9)	11.44	NO	NO	YES*
YoeB toxin protein	11.42	YES*	NO	YES*
hypothetical protein	11.21	NO	NO	YES*
Multidrug resistance protein A	10.62	YES*	NO	NO
Phosphoserine phosphatase	10.55	NO	NO	NO
hypothetical protein	10.49	NO	NO	NO
Fic family protein	9.89	YES*	NO	NO

hypothetical protein	9.88	NO	NO	NO
Putative membrane protein	9.70	YES*	NO	YES*
Predicted transcriptional regulator	9.67	YES*	NO	YES*
DNA-binding response regulator	9.54	YES*	NO	YES*
Sigma factor RpoE negative regulatory protein RseA	9.45	YES*	NO	YES*
pR99_vep31	9.38	NO	NO	YES*
Uxu operon transcriptional regulator	9.06	YES*	NO	YES*
Transposase and inactivated derivatives	8.95	YES*	NO	NO
hypothetical protein	8.79	NO	NO	NO
Flagellar biosynthesis protein FlgN	8.75	NO	NO	YES*
probable exported protein YPO3233	8.68	NO	NO	NO
surface localized decaheme cytochrome c lipoprotein, MtrC	8.66	NO	NO	YES
ABC-type uncharacterized transport system, permease component	8.61	NO	NO	NO
Transcriptional regulator, IclR family	8.53	YES*	NO	YES*
Ascorbate utilization transcriptional regulator UlaR, HTH-type	8.52	YES*	NO	YES*
S-adenosylmethionine synthetase (EC 2.5.1.6)	8.51	YES*	NO	YES*
Negative regulator of flagellin synthesis FlgM	8.50	YES*	NO	YES*
hypothetical protein	8.42	YES*	NO	YES*
Protein F-related protein	8.41	YES*	NO	YES*
RNA polymerase sigma-70 factor, ECF subfamily	8.37	NO	NO	YES*
Pyruvate formate-lyase activating enzyme (EC 1.97.1.4)	8.37	YES*	NO	YES*
Succinate-semialdehyde dehydrogenase [NADP+] (EC 1.2.1.16)	8.36	NO	NO	YES*
Low molecular weight protein tyrosine phosphatase (EC 3.1.3.48)	8.19	YES*	NO	YES*
Nitrite-sensitive transcriptional repressor NsrR	8.18	YES*	NO	YES*
hypothetical protein	8.01	NO	NO	NO
hypothetical protein	7.99	NO	NO	NO
Transcriptional regulator, TetR family	7.90	YES*	NO	YES*
Putative inner membrane protein YjeT (clustered with HflC)	7.89	NO	NO	YES*
Large exoproteins involved in heme utilization or adhesion	7.88	NO	NO	NO
FIG106692: Outer membrane lipoprotein	7.66	YES*	NO	YES*
Thymidylate kinase	7.55	NO	NO	NO
Flagellar motor rotation protein MotA	7.54	YES*	NO	YES*
Topoisomerase IV subunit B (EC 5.99.1.-)	7.50	NO	NO	NO
hypothetical protein	7.44	NO	NO	NO
DNA polymerase III psi subunit (EC 2.7.7.7)	7.41	NO	NO	YES*

pR99_vep56	7.40	NO	YES*	NO
Predicted DNA-binding protein	7.23	YES*	NO	YES*
Permease of the major facilitator superfamily	7.21	NO	NO	NO
RND efflux system, inner membrane transporter CmeB	7.19	NO	NO	NO
DNA polymerase III epsilon subunit (EC 2.7.7.7)	7.15	NO	NO	NO
Chemotactic transducer-related protein	7.13	NO	NO	YES*
ISBma1, transposase	7.05	YES*	NO	NO
Metallo-beta-lactamase family protein, RNA-specific	6.97	NO	NO	NO
FIG111991: hypothetical protein	6.90	NO	NO	YES*
Acriflavin resistance protein	6.87	NO	NO	NO
O-acetylhomoserine sulfhydrylase (EC 2.5.1.49)	6.81	NO	NO	NO
Cytochrome c-type protein NapC	6.70	NO	NO	NO
SN-glycerol-3-phosphate transport system permease protein UgpA (TC 3.A.1.1.3)	6.68	NO	NO	NO
Transcriptional regulator	6.66	NO	NO	NO
Nucleoside permease NupC	6.64	NO	NO	NO
Arylsulfatase (EC 3.1.6.1)	6.62	NO	NO	NO
Predicted exporter of the RND superfamily	6.58	NO	NO	YES*
Zn-dependent hydrolase (EC 3.-.-.-)	6.56	YES*	NO	YES*
hypothetical protein	6.54	NO	NO	YES*
Predicted hydrolase of the metallo-beta-lactamase superfamily, clustered with K	6.53	NO	NO	NO
Response regulator	6.44	NO	NO	NO
Sigma factor RpoE negative regulatory protein RseB precursor	6.42	YES*	NO	YES*
Transcriptional regulator	6.41	NO	NO	YES*
7,8-didemethyl-8-hydroxy-5-deazariboflavin synthase subunit 2	6.41	NO	NO	NO
Toxin secretion ATP-binding protein	6.38	NO	NO	NO
Permease of the major facilitator superfamily	6.35	NO	NO	NO
Iron-sulfur cluster-binding protein	6.33	NO	NO	YES
Probable L-ascorbate-6-phosphate lactonase UlaG (EC 3.1.1.-) (L-ascorbate utili	6.32	YES*	NO	YES*
hypothetical protein	6.32	NO	NO	NO
hypothetical protein	6.29	NO	NO	YES*
hypothetical protein	6.27	NO	NO	NO
Putative HTH-type transcriptional regulator ybaO	6.26	NO	NO	NO
Outer membrane lipoprotein-sorting protein	6.23	NO	NO	NO
Sigma factor RpoE regulatory protein RseC	6.22	YES*	NO	YES*
ISBma1, transposase	6.14	NO	NO	NO
TRAP-type C4-dicarboxylate transport system, large permease component	6.12	NO	NO	NO

hypothetical protein	6.11	NO	NO	NO
hypothetical protein	6.10	NO	NO	NO
Phosphoglycerate transport system transcriptional regulatory protein PgtA	6.08	NO	NO	NO
Rossmann fold nucleotide-binding protein Smf possibly involved in DNA uptake	6.06	NO	NO	YES*
Extracellular solute-binding protein, family 3/GGDEF domain protein	6.05	NO	NO	NO
hypothetical protein	6.04	NO	NO	YES*
HD-domain protein	6.03	NO	NO	NO
Nitrite transporter from formate/nitrite family	5.99	YES*	NO	YES
Tyrosyl-tRNA synthetase (EC 6.1.1.1) ## cluster 1	5.87	NO	NO	NO
Glutathione S-transferase (EC 2.5.1.18)	5.86	NO	NO	NO
Fusaric acid resistance protein fusE	5.84	NO	NO	NO
Biotin synthase (EC 2.8.1.6)	5.83	YES*	NO	NO
pR99_vep13	5.81	YES*	YES*	YES*
O-succinylbenzoate-CoA synthase (EC 4.2.1.-)	5.79	NO	NO	YES*
Flagellar synthesis regulator FleN	5.79	YES*	NO	YES*
Uridine phosphorylase (EC 2.4.2.3)	5.78	YES*	NO	YES*
hypothetical protein	5.78	NO	NO	NO
ABC-type sugar transport system, ATPase component	5.77	NO	NO	NO
Permease of the major facilitator superfamily	5.75	NO	NO	YES*
outer membrane protein, MtrB	5.75	NO	NO	NO
Dihydroneopterin triphosphate pyrophosphohydolase type 2	5.74	YES*	NO	YES*
pR99_vep58	5.73	NO	YES*	NO
ClpB protein	5.73	YES*	NO	YES*
Membrane-fusion protein	5.69	NO	NO	NO
Extracellular deoxyribonuclease Dns (EC 3.1.21.-)	5.67	YES*	NO	YES*
hypothetical protein	5.64	NO	NO	NO
hypothetical protein	5.63	NO	NO	YES
Multidrug resistance protein 2	5.62	NO	NO	NO
membrane protein	5.62	NO	NO	NO
hypothetical protein	5.60	NO	NO	NO
Transcriptional regulator, AraC family	5.59	NO	NO	NO
Murein endopeptidase	5.54	NO	NO	NO
Aspartate/tyrosine/aromatic aminotransferase	5.54	NO	NO	NO
pR99_vep04	5.50	NO	NO	NO
Capsular polysaccharide synthesis enzyme CpsB	5.49	YES*	NO	NO
FIG005666: putative helicase	5.47	NO	NO	NO
ABC-type amino acid transport/signal	5.45	NO	NO	NO

transduction system				
hypothetical protein	5.44	NO	NO	NO
Ribose/xylose/arabinose/galactoside ABC-type transport systems, permease compon	5.41	NO	NO	NO
RNA polymerase sigma factor for flagellar operon	5.41	YES*	NO	YES*
hypothetical protein	5.37	NO	NO	YES*
GGDEF family protein	5.37	NO	NO	YES*
D-alanyl-D-alanine carboxypeptidase (EC 3.4.16.4)	5.37	YES	NO	NO
Apolipoprotein N-acyltransferase (EC 2.3.1.-) / Copper homeostasis protein CutE	5.35	NO	NO	NO
Lipoprotein releasing system transmembrane protein LolE	5.29	YES*	NO	YES*
Ribose ABC transport system, ATP-binding protein RbsA (TC 3.A.1.2.1)	5.28	YES*	NO	NO
pR99_vep59	5.27	NO	YES*	NO
hypothetical protein	5.27	NO	NO	NO
Putative ATP-dependent Lon protease	5.24	NO	NO	YES*
Transcriptional regulator, MarR family	5.24	NO	NO	NO
hypothetical protein	5.24	NO	NO	YES*
GGDEF domain protein	5.20	NO	NO	NO
Biotin-protein ligase (EC 6.3.4.15) / Biotin operon repressor	5.17	YES*	NO	YES*
Membrane-associated zinc metalloprotease	5.16	NO	NO	YES*
FIG001592: Phosphocarrier protein kinase/phosphorylase, nitrogen regulation ass	5.15	YES*	NO	YES*
Anti anti-sigma regulatory factor SypA	5.14	NO	NO	NO
Potential queD like 2	5.04	YES*	NO	YES*
hypothetical protein	5.00	YES*	NO	NO
Serine/threonine protein kinase PrkC, regulator of stationary phase	4.96	NO	NO	NO
Acyl-phosphate:glycerol-3-phosphate O-acyltransferase PlsY	4.94	YES*	NO	YES*
CcdA protein (antitoxin to CcdB)	4.91	NO	NO	NO
Hypothetical protein in cluster with HutR, VCA0066 homolog	4.91	NO	NO	NO
Arylsulfatase (EC 3.1.6.1)	4.88	NO	NO	NO
hypothetical protein	4.86	NO	NO	YES*
Flavohemoprotein (Hemoglobin-like protein) (Flavohemoglobin) (Nitric oxide diox	4.86	YES	NO	NO
2,3-dihydroxybenzoate-AMP ligase (EC 2.7.7.58)	4.85	NO	NO	NO
Formate efflux transporter (TC 2.A.44 family)	4.80	YES*	NO	NO
hypothetical protein	4.77	NO	NO	NO
Permease of the drug/metabolite transporter (DMT) superfamily	4.75	NO	NO	YES*

Succinate dehydrogenase iron-sulfur protein (EC 1.3.99.1)	4.74	NO	NO	NO
hypothetical protein	4.74	NO	NO	NO
Nitrite reductase [NAD(P)H] small subunit (EC 1.7.1.4)	4.73	NO	NO	NO
Metallo-beta-lactamase superfamily protein PA0057	4.73	NO	NO	NO
hypothetical protein	4.69	NO	NO	NO
Bacterial surface protein	4.68	NO	NO	YES*
Anti-anti-sigma regulatory factor	4.67	NO	NO	YES
Flp pilus assembly protein TadD, contains TPR repeat	4.62	YES	NO	NO
hypothetical protein	4.61	NO	NO	NO
pR99_vep60	4.60	NO	NO	NO
hypothetical protein	4.59	NO	NO	NO
pR99_mazF///pR99_mazE	4.58	NO	NO	NO
hypothetical protein	4.57	NO	NO	NO
Membrane protein	4.57	YES	NO	YES
putative	4.57	NO	NO	NO
TRAP-type transport system, small permease component, predicted N-acetylneurami	4.56	NO	NO	NO
Zinc ABC transporter, periplasmic-binding protein ZnuA	4.56	NO	NO	NO
Protein of unknown function DUF81	4.54	NO	NO	YES*
Glutathione S-transferase (EC 2.5.1.18)	4.52	NO	NO	NO
Membrane-associated phospholipid phosphatase	4.52	NO	NO	NO
Oligo-1,6-glucosidase (EC 3.2.1.10)	4.50	NO	NO	NO
Chromosome segregation ATPase	4.45	NO	NO	NO
4-hydroxy-3-methylbut-2-enyl diphosphate reductase (EC 1.17.1.2)	4.44	NO	NO	YES*
probable extracellular solute-binding protein	4.37	NO	NO	NO
hypothetical protein	4.35	NO	NO	YES*
Transcriptional regulator, GntR family	4.34	NO	NO	NO
Maltose regulon regulatory protein MalI (repressor for malXY)	4.34	YES*	NO	YES*
Capsular polysaccharide synthesis enzyme CpsC, polysaccharide export	4.34	NO	NO	YES*
H(+)/Cl(-) exchange transporter ClcA	4.33	NO	NO	NO
Cell division protein FtsJ / Ribosomal RNA large subunit methyltransferase E (E	4.32	YES*	NO	YES*
Phosphoserine phosphatase (EC 3.1.3.3)	4.32	YES*	NO	NO
hypothetical protein	4.30	NO	NO	YES
Lactoylglutathione lyase	4.30	NO	NO	NO
ATP-dependent DNA helicase RecG (EC 3.6.1.-)	4.30	NO	NO	NO
Non-ribosomal peptide synthetase modules, siderophore biosynthesis	4.28	YES	NO	NO

Ribosome-associated heat shock protein implicated in the recycling of the 50S s	4.26	YES*	NO	YES*
pR99_vep12	4.26	NO	YES*	NO
Transcriptional regulator, LysR family, in formaldehyde detoxification operon	4.25	NO	NO	NO
Lipase-related protein	4.25	NO	NO	NO
TPR repeat protein	4.25	NO	NO	NO
Cobalamin synthase	4.24	NO	NO	YES*
hypothetical protein	4.23	NO	NO	YES*
Phosphoglycerol transferase	4.21	NO	NO	NO
ISBma1, transposase	4.20	NO	NO	NO
Transposase and inactivated derivatives	4.20	NO	NO	NO
DNA polymerase III beta subunit (EC 2.7.7.7)	4.19	NO	NO	NO
GGDEF family protein	4.17	YES*	NO	YES*
hypothetical protein	4.17	NO	NO	NO
Glutamate synthase [NADPH] large chain (EC 1.4.1.13)	4.15	NO	NO	NO
hypothetical protein	4.14	NO	NO	NO
1-deoxy-D-xylulose 5-phosphate synthase (EC 2.2.1.7)	4.14	NO	NO	NO
Transcriptional regulator, VCA0231 ortholog	4.13	NO	NO	NO
DNA-binding heavy metal response regulator	4.13	NO	NO	NO
Nucleoside permease NupC	4.13	NO	NO	NO
Flagellar basal-body P-ring formation protein FlgA	4.09	YES*	NO	YES*
Acetyltransferase	4.08	NO	NO	YES*
Response regulator of citrate/malate metabolism	4.06	NO	NO	NO
Regulatory protein RecX	4.05	NO	NO	NO
D-lactate dehydrogenase (EC 1.1.1.28)	4.01	NO	NO	YES*
Accessory colonization factor AcfD precursor	4.00	NO	NO	NO
Lumazine protein, riboflavin synthase homolog	4.00	YES*	NO	YES*
Glycerol-3-phosphate regulon repressor, DeoR family	4.00	NO	NO	NO
hypothetical protein	3.99	NO	NO	NO
hypothetical protein	3.97	NO	NO	NO
DNA-3-methyladenine glycosylase (EC 3.2.2.20)	3.96	NO	NO	NO
Protein of avirulence locus ImpE	3.95	NO	NO	NO
HIPA PROTEIN	3.95	NO	NO	NO
Error-prone repair protein UmuD	3.95	NO	NO	NO
Potassium channel protein	3.94	NO	NO	NO
hypothetical protein	3.94	YES*	NO	YES*
hypothetical protein	3.93	NO	NO	NO

hypothetical protein	3.92	NO	NO	YES*
hypothetical protein	3.92	YES*	NO	NO
CONSERVED MEMBRANE PROTEIN	3.89	NO	NO	NO
membrane protein	3.88	YES*	NO	NO
putative lipoprotein L	3.86	NO	NO	YES*
Methyl-accepting chemotaxis protein	3.85	YES*	NO	YES*
FIG067310: hypothetical protein	3.82	NO	NO	NO
Phosphate ABC transporter, periplasmic phosphate-binding protein PstS (TC 3.A.1	3.81	NO	NO	NO
S-adenosyl-L-methionine dependent methyltransferase, similar to cyclopropane-fa	3.78	YES	NO	YES*
hypothetical protein	3.76	NO	NO	NO
Helix-turn-helix protein, CopG family	3.75	YES*	NO	YES*
Gluconokinase (EC 2.7.1.12)	3.75	NO	NO	NO
Transposase and inactivated derivatives	3.75	NO	NO	NO
D-alanyl-D-alanine carboxypeptidase (EC 3.4.16.4)	3.75	NO	NO	NO
Outer membrane protein N, non-specific porin	3.74	NO	NO	YES*
Adenylate cyclase (EC 4.6.1.1)	3.73	NO	NO	NO
Putative cytoplasmic protein	3.73	NO	NO	NO
Transcriptional regulator VpsT	3.72	NO	NO	NO
hypothetical protein	3.71	NO	NO	NO
RTX toxins and related Ca2+-binding proteins	3.71	NO	NO	NO
Flagellum-specific ATP synthase FliI	3.70	NO	NO	NO
FOG: GGDEF domain	3.69	NO	NO	NO
Transcriptional regulator, AraC family	3.68	NO	NO	NO
Hypothetical protein DUF454	3.67	NO	NO	NO
Evolved beta-D-galactosidase, alpha subunit	3.66	NO	NO	NO
Sugar transferase SypR involved in lipopolysaccharide synthesis	3.66	NO	NO	NO
Cold shock protein CspD	3.66	NO	NO	NO
Pantothenate kinase type III, CoaX-like (EC 2.7.1.33)	3.66	NO	NO	NO
hypothetical protein	3.66	YES	NO	NO
Molybdenum ABC transporter, periplasmic molybdenum-binding protein ModA (TC 3.A	3.65	YES*	NO	NO
Hypothetical nudix hydrolase YeaB	3.65	YES	NO	YES
Hydrogen peroxide-inducible genes activator	3.64	NO	NO	NO
Phosphate transport ATP-binding protein PstB (TC 3.A.1.7.1)	3.63	NO	NO	NO
GNAT family acetyltransferase YiiD potentially involved in tRNA processing	3.61	NO	NO	YES*
4-amino-6-deoxy-N-Acetyl-D-hexosaminyl-(Lipid carrier) acetyltrasferase	3.61	NO	NO	YES*

Kynurenine 3-monooxygenase (EC 1.14.13.9)	3.60	NO	NO	NO
transposase and inactivated derivative	3.60	NO	NO	NO
Succinyl-CoA synthetase, alpha subunit	3.60	NO	NO	YES*
hypothetical protein	3.58	YES	NO	YES
Uncharacterized protein YtfM precursor	3.58	NO	NO	NO
FIG065221: ATPase, AAA family	3.58	NO	NO	NO
Flagellin protein FlaF	3.58	NO	NO	YES*
L-proline glycine betaine ABC transport system permease protein ProW (TC 3.A.1.	3.57	NO	NO	NO
Regulatory protein LuxO	3.57	YES*	NO	YES*
hypothetical protein	3.57	NO	NO	NO
Functional role page for Chaperone protein TorD	3.56	YES*	NO	YES*
Zinc-regulated TonB-dependent outer membrane receptor	3.55	NO	NO	NO
transposase and inactivated derivative	3.55	NO	NO	NO
Rrf2-linked NADH-flavin reductase	3.55	NO	NO	NO
Sodium-Choline Symporter	3.54	NO	NO	YES*
hypothetical protein	3.52	NO	NO	NO
hypothetical protein	3.51	NO	NO	YES
MSHA pilin protein MshA BUT NOT	3.51	YES*	NO	YES*
Zinc ABC transporter, inner membrane permease protein ZnuB	3.50	NO	NO	NO
5-Enolpyruvylshikimate-3-phosphate synthase (EC 2.5.1.19)	3.50	NO	NO	YES*
Lysophospholipase L2 (EC 3.1.1.5)	3.50	YES*	NO	YES*
Manganese superoxide dismutase (EC 1.15.1.1)	3.49	YES	NO	NO
transposase and inactivated derivative	3.49	NO	NO	NO
Nitrite reductase [NAD(P)H] small subunit (EC 1.7.1.4)	3.48	YES	NO	NO
Glycine cleavage system transcriptional activator GcvA	3.47	NO	NO	YES*
FIG002208: Acetyltransferase (EC 2.3.1.-)	3.47	YES*	NO	YES*
hypothetical protein	3.47	NO	NO	NO
Outer membrane protein assembly factor YaeT precursor	3.47	YES*	NO	NO
Membrane fusion protein of RND family multidrug efflux pump	3.46	NO	NO	NO
hypothetical protein	3.46	NO	NO	YES*
Hypotehtical protein in Cytochrome oxidase biogenesis cluster	3.44	NO	NO	NO
SeqA protein, negative modulator of initiation of replication	3.44	NO	NO	YES*
Putative MCP-type signal transduction protein	3.42	NO	NO	NO
hypothetical protein	3.42	NO	NO	NO
hypothetical protein	3.41	NO	NO	NO

hypothetical protein	3.40	NO	NO	YES*
hypothetical protein	3.40	YES*	NO	YES*
hypothetical protein	3.40	NO	NO	YES*
Translation initiation factor SUI1-related protein	3.39	YES*	NO	YES*
Ferric siderophore transport system, periplasmic binding protein TonB	3.38	NO	NO	NO
Functional role page for Anaerobic nitric oxide reductase transcription regulat	3.38	NO	NO	YES
Cytoplasmic copper homeostasis protein cutC	3.37	NO	NO	NO
transposase and inactivated derivative	3.37	NO	NO	NO
transposase and inactivated derivative	3.36	NO	NO	NO
pR99_vep19	3.36	NO	NO	NO
pR99_vep61	3.36	NO	NO	NO
Selenoprotein W-related protein	3.36	NO	NO	YES*
hypothetical protein	3.36	NO	NO	NO
hypothetical protein	3.35	NO	NO	NO
hypothetical protein	3.35	NO	NO	NO
hypothetical protein	3.35	NO	NO	NO
Ribonuclease D (EC 3.1.26.3)	3.34	YES*	NO	YES*
putative regulatory protein	3.33	NO	NO	NO
Capsular polysaccharide synthesis enzyme CpsA, sugar transferase	3.32	NO	NO	NO
Transcriptional regulator, AsnC family	3.32	NO	NO	YES*
tRNA nucleotidyltransferase (EC 2.7.7.21) (EC 2.7.7.25)	3.31	NO	NO	NO
GGDEF family protein	3.31	NO	NO	YES*
Putative deoxyribonuclease YjjV	3.31	YES*	NO	YES*
Mannose-6-phosphate isomerase (EC 5.3.1.8)	3.30	NO	NO	NO
Multidrug resistance protein B	3.30	NO	NO	NO
N-acetylglucosamine-1-phosphate uridyltransferase (EC 2.7.7.23) / Glucosamine-1	3.30	YES*	NO	YES*
Methyltransferase (EC 2.1.1.-)	3.29	YES*	NO	YES*
hypothetical protein	3.29	NO	NO	NO
hypothetical protein	3.29	NO	NO	YES*
Serine/threonine protein kinase (EC 2.7.11.1)	3.28	NO	NO	NO
hypothetical protein	3.27	NO	NO	NO
Chemotaxis protein CheD	3.27	NO	NO	NO
Type II/IV secretion system ATPase TadZ/CpaE, associated with Flp pilus assembl	3.27	NO	NO	NO
6-phosphogluconolactonase (EC 3.1.1.31), eukaryotic type	3.27	NO	NO	NO
hypothetical protein	3.26	NO	NO	NO
Methylcrotonyl-CoA carboxylase carboxyl	3.26	NO	NO	NO

transferase subunit (EC 6.4.1.4)				
radical activating enzyme	3.26	YES	NO	NO
Fucose permease	3.26	NO	NO	NO
TolA protein	3.26	NO	NO	NO
hypothetical protein	3.26	NO	NO	NO
ClpB protein	3.24	NO	NO	NO
Excinuclease ABC subunit C	3.24	YES*	NO	YES
Glutaredoxin	3.23	NO	NO	NO
Secreted trypsin-like serine protease	3.22	NO	NO	NO
Glycosidase	3.22	NO	NO	YES*
trypsin, putative	3.22	NO	NO	NO
Cysteine desulfurase CsdA-CsdE, sulfur acceptor protein CsdE	3.22	YES*	NO	NO
Excinuclease ABC, C subunit-like	3.22	NO	NO	NO
MSHA biogenesis protein MshO	3.21	NO	NO	NO
transposase and inactivated derivative	3.21	NO	NO	NO
proteinase inhibitor, putative	3.21	NO	NO	YES
hypothetical protein	3.20	NO	NO	NO
Type II/IV secretion system protein TadC, associated with Flp pilus assembly	3.20	NO	NO	NO
hypothetical protein	3.18	NO	NO	NO
Aspartate aminotransferase (EC 2.6.1.1)	3.17	NO	NO	NO
Chemotaxis regulator - transmits chemoreceptor signals to flagelllar motor comp	3.17	NO	NO	YES
transposase and inactivated derivative	3.17	NO	NO	NO
membrane protein	3.17	NO	NO	NO
hypothetical protein	3.17	NO	NO	NO
hypothetical protein	3.17	NO	NO	NO
hypothetical protein	3.16	NO	NO	NO
Para-aminobenzoate synthase, aminase component (EC 2.6.1.85) / Aminodeoxychoris	3.16	NO	NO	NO
Tryptophanase (EC 4.1.99.1)	3.15	NO	NO	YES
Magnesium and cobalt efflux protein CorC	3.15	NO	NO	YES*
transposase and inactivated derivative	3.15	NO	NO	NO
DNA primase (EC 2.7.7.-)	3.13	NO	NO	YES
FOG: WD40 repeat	3.13	NO	NO	NO
hypothetical protein	3.12	NO	NO	NO
TrkA, Potassium channel-family protein	3.12	NO	NO	NO
hypothetical protein	3.12	NO	NO	NO
Transcriptional regulator, AraC family	3.12	NO	NO	NO
conserved protein of unknown function; putative YcgN protein	3.12	NO	NO	NO
hypothetical protein	3.12	NO	NO	YES*
hypothetical protein	3.10	NO	NO	NO
Ribosome small subunit-stimulated GTPase	3.10	NO	NO	YES*

EngC				
hypothetical protein	3.09	NO	NO	NO
hypothetical protein	3.08	NO	NO	YES*
Leucyl/phenylalanyl-tRNA--protein transferase (EC 2.3.2.6)	3.08	NO	NO	NO
HipA protein	3.08	NO	NO	NO
Cytochrome oxidase biogenesis protein Surf1, facilitates heme A insertion	3.08	NO	NO	NO
Methyl-accepting chemotaxis protein	3.07	YES	NO	YES
Outer membrane protein romA	3.07	NO	NO	NO
Transcriptional regulator, SorC family	3.07	NO	NO	NO
Beta-hexosaminidase (EC 3.2.1.52)	3.06	NO	NO	NO
hypothetical protein	3.06	NO	NO	YES*
Propionate--CoA ligase (EC 6.2.1.17)	3.06	NO	NO	NO
Methyl-accepting chemotaxis protein	3.05	NO	NO	YES
Sulfate permease	3.04	NO	NO	NO
hypothetical protein	3.03	NO	NO	NO
hypothetical protein	3.03	NO	NO	YES
hypothetical protein	3.03	NO	NO	NO
Oligopeptide transport system permease protein OppC (TC 3.A.1.5.1)	3.02	YES*	NO	NO
conserved hypothetical protein	3.01	YES*	NO	NO
Tyrosine-specific transport protein	3.01	YES*	NO	YES*
Phosphate transport system permease protein PstA (TC 3.A.1.7.1)	3.00	NO	NO	NO
Type IV pilus biogenesis protein PilE	3.00	NO	NO	NO
Probable 3-phenylpropionic acid transporter	3.00	NO	NO	NO
Putative metal chaperone, involved in Zn homeostasis, GTPase of COG0523 family	3.00	NO	NO	YES*
Chaperone protein HtpG	2.99	YES*	NO	YES*
Transcriptional regulator, TetR family	2.99	YES*	NO	YES*
Two component response regulator	2.99	NO	NO	NO
hypothetical protein	2.99	NO	NO	NO
UDP-glucose dehydrogenase (EC 1.1.1.22)	2.98	NO	NO	NO
Phosphatidylglycerophosphatase A (EC 3.1.3.27)	2.98	NO	NO	NO
hypothetical protein	2.98	NO	NO	NO
Permease of the drug/metabolite transporter (DMT) superfamily	2.97	NO	NO	NO
Transcriptional regulator, MarR family	2.96	NO	NO	NO
Permease of the drug/metabolite transporter (DMT) superfamily	2.96	NO	NO	NO
ATP-dependent DNA ligase	2.96	NO	NO	YES*
Thiamin ABC transporter, transmembrane component	2.96	NO	NO	NO
PutR, transcriptional activator of PutA and PutP	2.96	NO	NO	NO
Signal transduction histidine kinase	2.96	NO	NO	NO

Transcriptional regulator, LysR family	2.95	NO	NO	NO
hypothetical protein	2.95	NO	NO	NO
Oligopeptidase A (EC 3.4.24.70)	2.95	NO	NO	NO
hypothetical protein	2.94	NO	NO	YES*
2-oxoglutarate dehydrogenase complex, dehydrogenase component	2.94	YES*	NO	NO
Oxygen-insensitive NAD(P)H nitroreductase (EC 1.-.-.-) / Dihydropteridine reduc	2.94	YES*	NO	YES*
Secreted trypsin-like serine protease	2.93	NO	NO	YES
N-acetylmuramoyl-L-alanine amidase (EC 3.5.1.28) AmpD	2.93	NO	NO	NO
Glycosyltransferase involved in cell wall biogenesis (EC 2.4.-.-)	2.93	NO	NO	NO
hypothetical protein	2.93	NO	NO	NO
cAMP-binding proteins - catabolite gene activator and regulatory subunit of cAM	2.92	NO	NO	YES
Isochorismatase (EC 3.3.2.1)	2.91	NO	NO	NO
hypothetical protein	2.91	NO	NO	YES*
pR99_vep62	2.90	NO	YES*	NO
UDP-Bac2Ac4Ac hydrolyzing 2-epimerase NeuC homolog	2.89	YES*	NO	YES*
HTH-type transcriptional regulator zntR	2.89	NO	NO	YES*
Phosphorelay protein LuxU	2.89	YES*	NO	YES*
Putative two-component response regulatory protein	2.89	NO	NO	NO
hypothetical protein	2.88	NO	NO	NO
Dihydrofolate reductase (EC 1.5.1.3)	2.87	NO	NO	NO
Mannose-6-phosphate isomerase (EC 5.3.1.8)	2.87	NO	NO	NO
Phosphate regulon sensor protein PhoR (SphS) (EC 2.7.13.3)	2.87	YES*	NO	YES*
hypothetical protein	2.87	NO	NO	NO
Probable transcriptional activator for leuABCD operon	2.86	NO	NO	NO
hypothetical protein	2.86	YES	NO	NO
COG2357: Uncharacterized protein conserved in bacteria	2.86	NO	NO	NO
AraC-type DNA-binding domain-containing protein	2.86	NO	NO	NO
ABC-type branched-chain amino acid transport system, periplasmic component	2.86	NO	NO	NO
Probable Co/Zn/Cd efflux system membrane fusion protein	2.85	YES	NO	NO
hypothetical protein	2.85	NO	NO	NO
ABC transporter, ATP-binding protein YnjD	2.84	NO	NO	NO
Potassium uptake protein, integral membrane component, KtrA	2.84	NO	NO	NO
hypothetical protein	2.83	YES*	NO	YES*

Transcriptional regulator, AraC family	2.83	NO	NO	NO
Flagellar motor rotation protein MotB	2.83	YES*	NO	NO
Biopolymer transport protein ExbD/TolR	2.83	NO	NO	NO
Putative exported protein	2.83	NO	NO	NO
hypothetical protein	2.83	YES*	NO	YES*
HflK protein	2.82	NO	NO	NO
Glutamate synthase [NADPH] large chain (EC 1.4.1.13)	2.82	NO	NO	NO
Serine protein kinase (prkA protein), P-loop containing	2.81	NO	NO	YES
3,4-dihydroxyphenylacetate 2,3-dioxygenase (EC 1.13.11.15)	2.81	NO	NO	NO
Tol biopolymer transport system, TolR protein	2.81	YES*	NO	NO
Nitrite reductase [NAD(P)H] large subunit (EC 1.7.1.4)	2.80	NO	NO	NO
Putative inner membrane protein	2.80	NO	NO	YES
FOG: GGDEF domain	2.79	NO	NO	NO
ABC-type protease/lipase transport system, ATPase and permease component	2.79	NO	NO	NO
hypothetical protein	2.78	NO	NO	NO
Glutamate-1-semialdehyde aminotransferase (EC 5.4.3.8)	2.78	YES	NO	NO
FIG002076: hypothetical protein	2.78	YES	NO	YES
Methyl-accepting chemotaxis protein	2.78	YES*	NO	YES*
pR99_vep68	2.77	NO	YES*	YES
Chitinase (EC 3.2.1.14)	2.76	NO	NO	NO
hypothetical protein	2.76	NO	NO	NO
hypothetical protein	2.75	NO	NO	NO
Dihydrolipoamide acyltransferase component of branched-chain alpha-keto acid de	2.75	NO	NO	NO
ABC-type sulfate transport system, permease component	2.74	NO	NO	NO
hypothetical protein	2.74	NO	NO	NO
hypothetical protein	2.74	NO	NO	NO
Lipase chaperone	2.73	NO	NO	NO
hypothetical protein	2.73	NO	NO	YES*
Threonine synthase (EC 4.2.3.1)	2.72	NO	NO	YES
Thioredoxin 2 (EC 1.8.1.8)	2.72	NO	NO	YES
Peptide methionine sulfoxide reductase MsrB (EC 1.8.4.12)	2.71	NO	NO	NO
Bll6819 protein	2.71	NO	NO	YES
NAD-specific glutamate dehydrogenase (EC 1.4.1.2), large form	2.71	NO	NO	NO
Oligopeptide transport system permease protein OppB (TC 3.A.1.5.1)	2.71	NO	NO	NO
Lipoate synthase	2.71	NO	NO	NO
Xanthosine phosphorylase (EC 2.4.2.1)	2.70	NO	NO	NO

Glucosamine-link cellobiase (EC 3.2.1.21)	2.70	NO	NO	NO
hypothetical protein	2.69	NO	NO	NO
3-deoxy-D-manno-octulosonate 8-phosphate phosphatase (EC 3.1.3.45)	2.69	NO	NO	YES*
hypothetical protein	2.68	YES	NO	YES*
pR99_vep48	2.68	YES	NO	YES
Hypothetical protein, specific for Vibrio	2.68	NO	NO	NO
Transcriptional regulator	2.67	NO	NO	NO
hypothetical protein	2.67	NO	NO	NO
Multidrug resistance protein	2.66	NO	NO	NO
hypothetical protein	2.66	NO	NO	NO
GGDEF family protein	2.66	NO	NO	YES
Signal recognition particle GTPase	2.66	NO	NO	NO
Transcriptional regulator SlmA, TetR family	2.65	NO	NO	YES*
hypothetical protein	2.65	NO	NO	NO
Putative oxidoreductase SMc00968	2.65	NO	NO	NO
2,3,4,5-tetrahydropyridine-2,6-dicarboxylate N-succinyltransferase (EC 2.3.1.11	2.65	YES*	NO	NO
Methyl-accepting chemotaxis protein	2.64	NO	NO	NO
L-beta-lysine 5,6-aminomutase alpha subunit (EC 5.4.3.3)	2.63	YES	NO	NO
DNA polymerase III epsilon subunit (EC 2.7.7.7)	2.63	NO	NO	NO
Pyridoxamine 5'-phosphate oxidase-related, FMN-binding	2.63	YES	NO	NO
hypothetical protein	2.62	NO	NO	NO
Aminodeoxychorismate lyase (EC 4.1.3.38)	2.62	NO	NO	NO
UDP-glucose dehydrogenase (EC 1.1.1.22)	2.62	NO	NO	NO
pR99_vep05	2.61	YES	YES	NO
Sulfur carrier protein adenylyltransferase ThiF	2.61	NO	NO	NO
putative histidinol phosphatase and related hydrolases of the PHP family	2.61	NO	NO	NO
Glutamate Aspartate periplasmic binding protein precursor GltI (TC 3.A.1.3.4)	2.61	NO	NO	YES
RNA polymerase sigma factor RpoH	2.60	NO	NO	YES*
Phosphate transport system permease protein PstA (TC 3.A.1.7.1)	2.59	NO	NO	NO
Multi antimicrobial extrusion protein (Na(+)/drug antiporter), MATE family of M	2.57	NO	NO	NO
hypothetical protein	2.57	NO	NO	NO
Na+/H+ antiporter NhaC	2.57	NO	NO	NO
Adenosine (5')-pentaphospho-(5")-adenosine pyrophosphohydrolase (EC 3.6.1.-)	2.57	YES*	NO	YES*
Diadenosine tetraphosphatase	2.56	NO	NO	NO
hypothetical protein	2.56	NO	NO	NO
Carboxynorspermidine decarboxylase,	2.55	YES*	NO	YES

putative (EC 4.1.1.-)				
HTH-type transcriptional regulator BetI	2.55	NO	NO	YES*
Transcriptional regulator	2.55	NO	NO	YES*
Methyl-accepting chemotaxis protein I (serine chemoreceptor protein)	2.55	NO	NO	NO
Putative response regulator	2.55	YES	NO	NO
AttF component of AttEFGH ABC transport system / AttG component of AttEFGH ABC	2.55	NO	NO	NO
FOG: CheY-like receiver	2.55	NO	NO	YES
Na+/H+ antiporter NhaA type	2.54	NO	NO	NO
Methyl-accepting chemotaxis protein I (serine chemoreceptor protein)	2.54	NO	NO	NO
Anthranilate synthase, amidotransferase component (EC 4.1.3.27)	2.54	NO	NO	YES*
hypothetical protein	2.53	NO	NO	NO
Proline/sodium symporter PutP (TC 2.A.21.2.1) @ Propionate/sodium symporter	2.52	NO	NO	NO
hypothetical protein	2.52	NO	NO	YES*
Maleylacetoacetate isomerase (EC 5.2.1.2) @ Glutathione S-transferase, zeta (EC	2.52	NO	NO	NO
Bacterioferritin-associated ferredoxin	2.52	YES	NO	YES
Possible sterol desaturase	2.51	NO	NO	NO
hypothetical protein	2.51	NO	NO	YES
membrane protein, putative	2.51	NO	NO	NO
Alkaline phosphatase (EC 3.1.3.1)	2.51	NO	NO	NO
Lysine efflux permease	2.50	NO	NO	YES*
L-xylulose 5-phosphate 3-epimerase (EC 5.1.3.-)	2.50	NO	NO	NO
Heavy-metal-associated domain (N-terminus) and membrane-bounded cytochrome biog	2.50	NO	NO	YES
Arginine pathway regulatory protein ArgR, repressor of arg regulon	2.49	YES	NO	NO
hypothetical protein	2.49	NO	NO	YES*
Chitinase (EC 3.2.1.14)	2.49	NO	NO	YES*
Methionine ABC transporter ATP-binding protein	2.48	YES	NO	YES*
hypothetical protein	2.48	NO	NO	NO
Outer membrane protein YfgL, lipoprotein component of the protein assembly comp	2.48	YES*	NO	NO
membrane protein	2.48	YES*	NO	YES*
Na+/H+ antiporter	2.47	YES*	NO	NO
Inosine/xanthosine triphosphatase (EC 3.6.1.-); Hypothetical cytoplasmic protei	2.46	NO	NO	NO
hypothetical protein	2.46	NO	NO	NO
Ubiquinone/menaquinone biosynthesis methyltransferase UbiE (EC 2.1.1.-)	2.46	YES*	NO	YES*
Carbonic anhydrase (EC 4.2.1.1)	2.46	YES*	NO	YES*
Transcriptional regulatory protein UhpA	2.45	NO	NO	NO

GTP-binding protein Era	2.45	NO	NO	NO
putative acetyltransferase	2.45	NO	NO	NO
membrane protein	2.44	NO	NO	YES
Transcriptional regulator, LysR family	2.43	NO	NO	NO
Putative cell envelope opacity-associated protein A	2.43	NO	NO	YES*
Transcriptional regulators, LysR family	2.42	NO	NO	NO
DNA mismatch repair protein MutL	2.42	NO	NO	NO
hypothetical protein	2.42	NO	NO	NO
Prolyl endopeptidase (EC 3.4.21.26)	2.41	NO	NO	NO
tRNA-specific adenosine-34 deaminase (EC 3.5.4.-)	2.41	NO	NO	NO
ribosomal protein S6 glutaminyl transferase related protein	2.41	YES*	NO	YES*
Signal peptidase I (EC 3.4.21.89)	2.40	NO	NO	NO
Transcriptional regulator, LacI family	2.40	NO	NO	NO
hypothetical protein	2.40	NO	NO	NO
DNA recombination protein RmuC	2.39	NO	NO	NO
hypothetical protein	2.39	NO	NO	NO
Ribosomal-protein-S18p-alanine acetyltransferase (EC 2.3.1.-)	2.38	NO	NO	NO
hypothetical protein	2.37	NO	NO	NO
Flagellin protein FlaD	2.37	YES*	NO	YES*
Transcriptional regulator of succinyl CoA synthetase operon	2.37	YES*	NO	YES*
Cytoplasmic axial filament protein CafA and Ribonuclease G (EC 3.1.4.-)	2.37	NO	NO	YES*
UDP-N-acetylenolpyruvoylglucosamine reductase (EC 1.1.1.158)	2.37	YES*	NO	YES*
Hypothetical protein DUF454	2.36	NO	NO	NO
hypothetical protein	2.36	NO	NO	NO
Permeases of the major facilitator superfamily	2.36	NO	NO	NO
Multidrug efflux pump component MtrF	2.36	NO	NO	NO
pR99_vep69	2.35	YES	NO	YES
FIG000506: Hypothetical ATP-binding protein	2.34	NO	NO	NO
hypothetical protein	2.34	NO	NO	YES
Glutathione S-transferase (EC 2.5.1.18)	2.34	NO	NO	NO
Gamma-glutamyl phosphate reductase (EC 1.2.1.41)	2.34	NO	NO	YES
Probable transcriptional activator for leuABCD operon	2.33	NO	NO	NO
Fumarylacetoacetase (EC 3.7.1.2)	2.33	NO	NO	NO
AttH component of AttEFGH ABC transport system	2.33	NO	NO	YES
TRAP dicarboxylate transporter, DctQ subunit, unknown substrate 3	2.33	NO	NO	NO
Putative threonine efflux protein	2.33	NO	NO	NO

hypothetical protein	2.32	YES	NO	YES
hypothetical protein	2.32	NO	NO	YES
Histone acetyltransferase HPA2	2.32	NO	NO	NO
Leader peptidase (Prepilin peptidase) (EC 3.4.23.43) / N-methyltransferase (EC	2.31	NO	NO	NO
SAM-dependent methyltransferases	2.31	NO	NO	NO
pR99_vep24	2.30	YES	YES	NO
Ferric siderophore transport system, periplasmic binding protein TonB	2.30	NO	NO	YES
Molybdenum cofactor biosynthesis protein MoaC	2.30	NO	NO	NO
Inner membrane protein translocase component YidC, long form	2.29	NO	NO	NO
Legionaminic acid synthase (EC 2.5.1.56)	2.29	YES*	NO	YES*
hypothetical protein	2.29	NO	NO	YES*
Chromate transport protein ChrA	2.29	NO	NO	NO
Spermidine synthase-like protein	2.29	NO	NO	NO
Potassium uptake protein TrkH	2.29	NO	NO	NO
UPF0246 protein YaaA	2.29	NO	NO	YES*
S-(hydroxymethyl)glutathione dehydrogenase (EC 1.1.1.284)	2.28	NO	NO	NO
Transcriptional regulator, LysR family	2.28	NO	NO	NO
Cell division inhibitor	2.28	NO	NO	YES*
L-proline glycine betaine ABC transport system permease protein ProV (TC 3.A.1.	2.27	NO	NO	NO
Acylphosphate phosphohydrolase (EC 3.6.1.7), putative	2.27	NO	NO	NO
Transcriptional regulator, TetR family	2.27	YES*	NO	NO
ABC transporter ATP-binding protein YvcR	2.26	YES	NO	YES*
hypothetical protein	2.26	NO	NO	NO
Outer membrane protein SypB	2.26	NO	NO	NO
BarA-associated response regulator UvrY (= GacA = SirA)	2.26	NO	NO	YES*
Chorismate synthase (EC 4.2.3.5)	2.26	YES*	NO	YES*
Transcriptional regulator, ArsR family	2.25	NO	NO	YES*
Flagellin protein FlaG	2.25	YES*	NO	NO
MSHA biogenesis protein MshP	2.24	NO	NO	YES
RTX toxin	2.24	NO	NO	NO
Octanoate-[acyl-carrier-protein]-protein-N-octanoyltransferase	2.24	NO	NO	YES*
Soluble lytic murein transglycosylase precursor (EC 3.2.1.-)	2.23	YES*	NO	YES*
Protein-export membrane protein SecD (TC 3.A.5.1.1)	2.23	NO	NO	NO
Signal transduction histidine kinase	2.22	YES	NO	YES
Predicted methylated DNA-protein cysteine methyltransferase	2.21	NO	NO	NO
Flagellin protein FlaD	2.21	NO	NO	NO
transposase and inactivated derivative	2.21	NO	NO	NO

Cobyric acid synthase	2.20	YES*	NO	NO
5,10-methylenetetrahydrofolate reductase (EC 1.5.1.20)	2.20	NO	NO	NO
Transcriptional regulator, AraC family	2.20	NO	NO	NO
hypothetical protein	2.20	YES	NO	YES
UDP-N-acetylmuramoylalanine--D-glutamate ligase (EC 6.3.2.9)	2.20	NO	NO	YES
tRNA-(ms[2]io[6]A)-hydroxylase (EC 1.-.-.-)	2.20	NO	NO	YES
Uncharacterized protein	2.20	NO	NO	NO
Permease of the major facilitator superfamily	2.19	NO	NO	NO
Putative magnesium transporter MgtE	2.19	NO	NO	YES*
hypothetical protein	2.19	NO	NO	NO
Flagellar motor switch protein FliM	2.19	YES*	NO	NO
ABC-type uncharacterized transport system, periplasmic component	2.18	NO	NO	NO
Sulfur carrier protein ThiS	2.18	NO	NO	NO
pR99_vep27	2.18	NO	YES*	YES*
Exodeoxyribonuclease V alpha chain (EC 3.1.11.5) ## RecD	2.18	NO	NO	NO
hypothetical protein	2.17	NO	NO	NO
Outer membrane protein C precursor	2.17	NO	NO	NO
ElaA protein	2.17	NO	NO	NO
hypothetical protein	2.16	NO	NO	NO
Multidrug resistance protein D	2.16	NO	NO	NO
hypothetical protein	2.15	YES	NO	YES*
ADP-heptose synthase (EC 2.7.-.-) / D-glycero-beta-D-manno-heptose 7-phosphate	2.14	YES*	NO	YES*
AsmA protein	2.14	NO	NO	NO
Chaperone protein DnaJ	2.13	NO	NO	NO
hypothetical protein	2.13	NO	NO	NO
Putative threonine efflux protein	2.13	NO	NO	NO
Protein of unknown function Smg	2.12	YES*	NO	YES*
hypothetical protein	2.12	NO	NO	NO
Methylmalonate-semialdehyde dehydrogenase (EC 1.2.1.27)	2.12	NO	NO	NO
MSHA biogenesis protein MshJ	2.12	NO	NO	YES
ATPase of the AAA+ class	2.11	NO	NO	YES*
Uncharacterized protein conserved in bacteria, NMA0228-like	2.11	NO	NO	NO
Guanylate cyclase-related protein	2.11	NO	NO	NO
ATP-dependent Clp protease ATP-binding subunit ClpX	2.11	YES*	NO	YES*
Protein YigP (COG3165) clustered with ubiquinone biosynthetic genes	2.10	YES*	NO	YES*
Flagellar M-ring protein FliF	2.10	NO	NO	NO
Bacterioferritin	2.10	YES	NO	YES

Fumarate and nitrate reduction regulatory protein	2.09	NO	NO	NO
Membrane-fusion protein	2.09	YES	NO	NO
CMP-N-acetylneuraminate-beta-galactosamide-alpha-2,3-sialyltransferase (EC 2.4.	2.08	NO	NO	NO
3-polyprenyl-4-hydroxybenzoate carboxy-lyase UbiX (EC 4.1.1.-)	2.08	NO	NO	YES*
Putative multidrug resistance protein	2.08	NO	NO	NO
Functional role page for TorCAD operon transcriptional regulatory protein TorR	2.07	NO	NO	NO
Argininosuccinate synthase (EC 6.3.4.5)	2.07	NO	NO	NO
hypothetical protein	2.07	NO	NO	YES*
Permease of the drug/metabolite transporter (DMT) superfamily	2.07	YES	NO	YES
General secretion pathway protein J	2.07	NO	NO	NO
Molybdenum transport system permease protein ModB (TC 3.A.1.8.1)	2.07	NO	NO	NO
hypothetical protein	2.06	NO	NO	YES*
pR99_vep70	2.06	NO	NO	NO
hypothetical protein	2.06	NO	NO	YES
Flagellar sensor histidine kinase FleS	2.06	NO	NO	NO
Siroheme synthase / Precorrin-2 oxidase (EC 1.3.1.76) / Sirohydrochlorin ferroc	2.06	NO	NO	NO
hypothetical protein	2.05	NO	NO	NO
hypothetical protein	2.05	NO	NO	NO
pR99_vep20	2.05	YES	YES	YES
hypothetical protein	2.05	NO	NO	YES
Ferrous iron transport protein B	2.04	YES	NO	YES
2-keto-3-deoxy-D-arabino-heptulosonate-7-phosphate synthase I alpha (EC 2.5.1.5	2.04	YES	NO	NO
hypothetical protein	2.04	NO	NO	NO
N-Ribosylnicotinamide phosphorylase (EC 2.4.2.1)	2.04	NO	NO	NO
ABC transporter, periplasmic spermidine putrescine-binding protein PotD (TC 3.A	2.04	NO	NO	NO
1,4-alpha-glucan branching enzyme (EC 2.4.1.18)	2.04	YES*	NO	YES*
Transcriptional regulator	2.04	NO	NO	YES*
hypothetical protein	2.03	YES*	NO	YES*
POTASSIUM/PROTON ANTIPORTER ROSB	2.03	NO	NO	YES
Glutamine amidotransferases class-II	2.03	YES	NO	NO
hypothetical protein	2.03	NO	NO	NO
hypothetical protein	2.03	NO	NO	YES*
COG4123: Predicted O-methyltransferase	2.03	NO	NO	NO
Transcriptional regulator, TetR family	2.03	NO	NO	NO
membrane protein	2.02	NO	NO	NO
FIG005080: Possible exported protein	2.02	NO	NO	NO

Membrane-associated phospholipid phosphatase	2.02	NO	NO	YES*
Oxalate/formate antiporter	2.02	NO	NO	NO
Beta-N-acetylhexosaminidase, (GlcNAc)2 catabolism	2.02	NO	NO	NO
Gluconate utilization system Gnt-I transcriptional repressor	2.01	YES	NO	YES*
transposase	2.01	YES	NO	YES
CheW domain protein	2.01	NO	NO	NO
Type IV pilin PilA	2.01	NO	NO	NO
High-affinity choline uptake protein BetT	2.01	NO	NO	NO
ADP-L-glycero-D-manno-heptose-6-epimerase (EC 5.1.3.20)	2.00	NO	NO	YES*
hypothetical protein	-2.00	NO	NO	NO
Glutaredoxin	-2.01	NO	NO	YES*
Putative signal peptide protein	-2.01	NO	NO	NO
hypothetical protein	-2.01	NO	NO	NO
tRNA (cytosine34-2'-O-)-methyltransferase (EC 2.1.1.-)	-2.01	YES*	NO	NO
Putrescine aminotransferase (EC 2.6.1.82)	-2.02	NO	NO	YES*
hypothetical protein	-2.02	YES*	NO	YES*
Na+/H+ antiporter, putative	-2.02	YES*	NO	NO
Na(+)-translocating NADH-quinone reductase subunit F (EC 1.6.5.-)	-2.02	NO	NO	NO
hypothetical protein	-2.02	NO	NO	YES*
hypothetical protein	-2.03	NO	NO	YES*
Probable low-affinity inorganic phosphate transporter	-2.04	NO	NO	YES*
Membrane alanine aminopeptidase N (EC 3.4.11.2)	-2.05	YES	NO	NO
hypothetical protein	-2.05	NO	NO	NO
Stringent starvation protein B	-2.05	NO	NO	NO
Ferritin-like protein 2	-2.06	NO	NO	YES
Predicted sodium/dicarboxylate symporter	-2.07	NO	NO	NO
Histidinol-phosphatase (EC 3.1.3.15) / Imidazoleglycerol-phosphate dehydratase	-2.09	NO	NO	NO
ATP-dependent RNA helicase RhlE	-2.09	NO	NO	YES*
SSU ribosomal protein S9p (S16e)	-2.09	NO	NO	YES*
NAD(FAD)-utilizing dehydrogenase, sll0175 homolog	-2.09	NO	NO	NO
ATPase involved in DNA repair	-2.10	NO	NO	YES*
Putative protease	-2.11	NO	NO	NO
hypothetical protein	-2.11	NO	NO	YES*
Phosphoribosylformimino-5-aminoimidazole carboxamide ribotide isomerase (EC 5.3	-2.11	NO	NO	NO
3-oxoacyl-[acyl-carrier-protein] synthase, KASIII (EC 2.3.1.41)	-2.12	NO	NO	NO
Pantoate--beta-alanine ligase (EC 6.3.2.1)	-2.12	NO	NO	NO

hypothetical protein	-2.13	NO	NO	YES*
Uncharacterized low-complexity protein	-2.13	NO	NO	NO
Probable exported or periplasmic protein in ApbE locus	-2.13	NO	NO	NO
LSU m5C1962 methyltransferase RlmI	-2.13	NO	NO	NO
Transcriptional regulator, LysR family	-2.14	NO	NO	NO
SSU ribosomal protein S20p	-2.14	YES*	NO	NO
Preprotein translocase subunit YajC (TC 3.A.5.1.1)	-2.15	NO	NO	NO
Uncharacterized ABC transporter, auxiliary component YrbC	-2.16	NO	NO	NO
Small-conductance mechanosensitive channel	-2.16	NO	NO	YES*
DNA topoisomerase III (EC 5.99.1.2)	-2.16	NO	NO	YES*
Alanyl-tRNA synthetase (EC 6.1.1.7)	-2.17	NO	NO	NO
Oxygen-insensitive NAD(P)H nitroreductase (EC 1.-.-.-) / Dihydropteridine reduc	-2.17	NO	NO	NO
Probable GTPase related to EngC	-2.17	YES	NO	YES
N-acetylglucosamine-6-phosphate deacetylase (EC 3.5.1.25)	-2.19	YES	NO	YES
Transcriptional activator RfaH	-2.19	YES	NO	NO
LppC putative lipoprotein	-2.20	YES*	NO	NO
Methylase of polypeptide chain release factors	-2.21	NO	NO	NO
hypothetical protein	-2.21	YES*	NO	YES*
Syd protein	-2.23	NO	NO	NO
Transcription termination factor Rho	-2.23	NO	NO	NO
Uncharacterized conserved protein	-2.23	NO	NO	NO
Histone acetyltransferase HPA2 and related acetyltransferases	-2.24	NO	NO	NO
Endonuclease IV (EC 3.1.21.2)	-2.25	NO	NO	YES
Cytosine deaminase (EC 3.5.4.1)	-2.26	NO	NO	NO
Uncharacterized conserved protein	-2.26	NO	NO	NO
hypothetical protein	-2.26	NO	NO	YES*
Putative threonine efflux protein	-2.27	NO	NO	NO
Histone acetyltransferase HPA2	-2.28	NO	NO	NO
Phosphogluconate repressor HexR, RpiR family	-2.28	NO	NO	NO
Methyltransferase (EC 2.1.1.-)	-2.29	NO	NO	NO
hypothetical protein	-2.29	YES*	NO	NO
UDP-N-acetylmuramate--alanine ligase (EC 6.3.2.8)	-2.31	NO	NO	NO
Ribosomal protein L11 methyltransferase (EC 2.1.1.-)	-2.32	NO	NO	NO
DNA-binding protein HU-beta	-2.35	NO	NO	NO
Topoisomerase IV subunit A (EC 5.99.1.-)	-2.35	NO	NO	NO
RNA methyltransferase, TrmA family	-2.35	NO	NO	YES*
Glutaminase (EC 3.5.1.2)	-2.35	NO	NO	YES*

Transposase	-2.35	YES*	NO	NO
Short chain fatty acids transporter	-2.36	NO	NO	NO
3-oxoacyl-[acyl-carrier protein] reductase (EC 1.1.1.100)	-2.36	NO	NO	NO
Acetyltransferase	-2.38	NO	NO	NO
Na(+)-translocating NADH-quinone reductase subunit E (EC 1.6.5.-)	-2.39	NO	NO	NO
Probable type IV pilus assembly FimV-related transmembrane protein	-2.39	NO	NO	YES*
YrbA protein	-2.39	NO	NO	NO
Putative chemotaxis protein CheY	-2.40	NO	NO	YES*
6-phosphofructokinase (EC 2.7.1.11)	-2.40	NO	NO	YES
Predicted amidohydrolase	-2.42	NO	NO	NO
Acyl-CoA thioesterase YciA, involved in membrane biogenesis	-2.44	NO	NO	NO
S-ribosylhomocysteine lyase (EC 4.4.1.21) / Autoinducer-2 production protein Lu	-2.45	NO	NO	NO
hypothetical protein	-2.46	NO	NO	NO
Putative cytoplasmic protein	-2.47	YES	NO	NO
Hypothetical protein VC0266 (sugar utilization related?)	-2.48	NO	NO	NO
Putative membrane protein	-2.50	NO	NO	NO
Thiamine biosynthesis protein thiI	-2.52	NO	NO	YES*
Pyruvate/2-oxoglutarate dehydrogenase complex, dihydrolipoamide dehydrogenase c	-2.53	YES*	NO	YES*
hypothetical protein	-2.54	YES*	NO	YES
Shikimate kinase I (EC 2.7.1.71)	-2.55	NO	NO	NO
Acyl carrier protein (ACP1)	-2.57	NO	NO	YES
Rod shape-determining protein MreB	-2.58	NO	NO	NO
hypothetical protein	-2.58	YES*	NO	YES*
3-dehydroquinate dehydratase II (EC 4.2.1.10)	-2.59	NO	NO	NO
putative transport system permease protein	-2.60	NO	NO	NO
Cytochrome c-type protein NrfB precursor	-2.60	NO	NO	YES*
Alcohol dehydrogenase (EC 1.1.1.1)	-2.60	NO	NO	NO
Ribose-phosphate pyrophosphokinase (EC 2.7.6.1)	-2.61	NO	NO	NO
Autoinducer 2 sensor kinase/phosphatase LuxQ (EC 2.7.3.-) (EC 3.1.3.-)	-2.62	YES*	NO	YES*
Aerobic respiration control protein arcA	-2.63	NO	NO	YES*
OsmC/Ohr family protein	-2.63	NO	NO	YES
Phospho-N-acetylmuramoyl-pentapeptide-transferase (EC 2.7.8.13)	-2.63	NO	NO	NO
Nitrogen regulation protein NR(II) (EC 2.7.3.-)	-2.66	NO	NO	NO
Putative cytochrome d ubiquinol oxidase subunit III (EC 1.10.3.-) (Cytochrome b	-2.66	NO	NO	NO
Adenylate kinase (EC 2.7.4.3)	-2.67	YES	NO	YES

hypothetical protein	-2.68	NO	NO	NO
Nucleoid-associated protein NdpA	-2.70	NO	NO	YES*
ABC transporter, periplasmic spermidine putrescine-binding protein PotD (TC 3.A	-2.72	YES*	NO	YES*
Alkylphosphonate utilization operon protein PhnA	-2.72	NO	NO	NO
S-adenosylmethionine:tRNA ribosyltransferase-isomerase (EC 5.-.-.-)	-2.74	NO	NO	NO
Methyl-accepting chemotaxis protein	-2.77	NO	NO	NO
Transcription termination protein NusB	-2.82	YES*	NO	YES*
Polysaccharide export lipoprotein Wza	-2.82	NO	NO	YES*
Transcriptional regulator, TetR family	-2.82	NO	NO	NO
2-keto-4-pentenoate hydratase (EC 4.2.1.-)	-2.84	NO	NO	NO
Putative sugar isomerase involved in processing of exogenous sialic acid	-2.84	NO	NO	NO
SSU ribosomal protein S21p	-2.91	YES*	NO	YES
Ribonucleotide reductase of class III (anaerobic), large subunit (EC 1.17.4.2)	-2.93	NO	NO	NO
Vitamin B12 ABC transporter, permease component BtuC	-2.97	YES*	NO	NO
Preprotein translocase subunit SecE (TC 3.A.5.1.1)	-2.98	YES	NO	NO
Lipoprotein nlpI precursor	-3.02	NO	NO	NO
Permease of the major facilitator superfamily	-3.06	NO	NO	YES*
hypothetical protein	-3.06	NO	NO	NO
LSU ribosomal protein L27p	-3.09	NO	NO	NO
Maltose/maltodextrin ABC transporter, permease protein MalG	-3.10	NO	NO	NO
Aspartokinase (EC 2.7.2.4)	-3.14	YES	NO	YES
Regulator of competence-specific genes	-3.16	YES	NO	YES*
hypothetical protein	-3.19	NO	NO	NO
hypothetical protein	-3.25	NO	NO	NO
Acyl carrier protein (ACP2)	-3.27	YES*	NO	NO
hypothetical protein	-3.31	NO	NO	NO
Acetyltransferase	-3.33	NO	NO	NO
Thioredoxin	-3.34	YES*	NO	NO
Adenosine deaminase (EC 3.5.4.4)	-3.34	NO	NO	NO
Cell division protein FtsK	-3.35	NO	NO	NO
hypothetical protein	-3.37	NO	NO	NO
ABC-type multidrug transport system, ATPase and permease component	-3.37	NO	NO	YES*
Ribose ABC transport system, ATP-binding protein RbsA (TC 3.A.1.2.1)	-3.39	NO	NO	NO
hypothetical protein	-3.41	NO	NO	NO
hypothetical protein	-3.46	NO	NO	NO
ATP-dependent RNA helicase VC1407	-3.49	NO	NO	NO
hypothetical protein	-3.53	NO	NO	NO

hypothetical protein	-3.53	YES*	NO	NO
YrdC/Sua5 family protein, required for threonylcarbamoyladenosine (t(6)A) forma	-3.55	YES*	NO	NO
Quinolinate phosphoribosyltransferase [decarboxylating] (EC 2.4.2.19)	-3.63	NO	NO	NO
SH3 domain protein	-3.63	NO	NO	YES*
Cytochrome c553	-3.64	NO	NO	NO
tRNA-guanine transglycosylase (EC 2.4.2.29)	-3.73	NO	NO	NO
Ribose ABC transport system, high affinity permease RbsD (TC 3.A.1.2.1)	-3.76	NO	NO	YES*
Histone acetyltransferase HPA2	-3.82	NO	NO	NO
YaeQ protein	-4.00	NO	NO	YES*
hypothetical protein	-4.06	NO	NO	NO
ATP-dependent RNA helicase DbpA	-4.09	NO	NO	YES*
hypothetical protein	-4.29	NO	NO	NO
Protein-export membrane protein SecD (TC 3.A.5.1.1)	-4.50	NO	NO	NO
Purine nucleotide synthesis repressor	-4.50	YES	NO	NO
Queuosine biosynthesis QueD, PTPS-I	-4.84	NO	NO	YES*
Guanylate kinase (EC 2.7.4.8)	-4.91	NO	NO	NO
Uncharacterized protein conserved in bacteria	-5.63	NO	NO	YES
Regulator of nucleoside diphosphate kinase	-5.64	NO	NO	NO
Uncharacterized protein conserved in bacteria	-7.96	NO	NO	NO
