# Supplementary material for: The Effect of the Environmental Temperature on the Adaptation to Host in the Zoonotic Pathogen Vibrio vulnificus
Source: Front Microbiol. 2020 Mar 27;11:489. doi: 10.3389/fmicb.2020.00489 (PMC7137831; doi:10.3389/fmicb.2020.00489)
Supplement: TABLE S4 — Differentially expressed genes by V. vulnificus at 37°C vs. 20°C in CM9. The fold change value for each gene is indicated with also the inclusion of those previously described to be differentially expressed in iron stimulon, fur regulon and eel serum (Pajuelo et al., 2016; Hernández-Cabanyero et al., 2019). ∗: present in iron stimulon, fur regulon or eel serum but with upside downregulation. ∗∗: only genes with values of fold change −2 ≤ X ≤ 2 with a p-value cut-off of 0.05 at 37°C vs. 20°C were considered. +: gene upregulated at 37°C; -: gene downregulated at 37°C. [file Data_Sheet_4.PDF]

Table S4. Differentially expressed genes by *V. vulnificus* at 37ºC vs 20ºC in CM9. The fold change value for each gene is indicated with also the inclusion of those previously described to be differentially expressed in iron stimulon, fur regulon and eel serum (Pajuelo et al., 2016; Hernández-Cabanyero et al., 2019).
*: present in iron stimulon, fur regulon or eel serum but with upside downregulation.
**: only genes with values of fold change -2≤X≤2 with a p-value cut-off of 0.05 at 37ºC vs 20ºC were considered. +: gene upregulated at 37ºC; -: gene downregulated at 37ºC.

Gene	Fold change**	Iron stimulon	Fur regulon	Iron-overloaded human serum
hypothetical protein	10.44	YES	NO	YES*
DNA polymerase III psi subunit (EC 2.7.7.7)	2.63	NO	NO	YES*
hypothetical protein	12.50	NO	NO	YES*
Acetyltransferase	21.90	NO	NO	YES*
Lipoate synthase	2.68	NO	NO	YES*
Membrane-associated phospholipid phosphatase	2.22	NO	NO	YES*
putative outer membrane lipoprotein	2.35	NO	NO	YES*
Plasmid-related protein	3.44	YES	NO	YES*
hypothetical protein	2.46	NO	NO	YES*
Transcriptional regulator, LuxR family	2.24	NO	NO	YES*
Secreted trypsin-like serine protease	4.09	NO	NO	YES*
DNA uptake protein	3.96	NO	NO	YES*
FIG023406: hypothetical protein	8.41	NO	NO	YES*
Phosphomethylpyrimidine kinase (EC 2.7.4.7)	4.76	NO	NO	YES*
Ribonuclease BN (EC 3.1.-.-)	3.62	NO	NO	YES*
hypothetical protein	6.32	YES	NO	YES*
putative acetyltransferase	3.91	NO	NO	YES*
Response regulator	4.94	NO	NO	YES*
hypothetical protein	4.78	NO	NO	YES*
putative regulatory protein	6.10	NO	NO	YES*
Type IV pilus biogenesis protein PilE	2.98	NO	NO	YES*
Arginine deiminase (EC 3.5.3.6)	2.66	YES	NO	YES*
tRNA pseudouridine synthase C (EC 4.2.1.70) ## tRNA Psi65	2.92	NO	NO	YES*
Methionine ABC transporter ATP-binding protein	2.19	YES	NO	YES*
Protein ydjA	2.92	YES	NO	YES*
Potassium uptake protein, integral membrane component, KtrA	3.43	NO	NO	YES*
Glutaredoxin	2.26	YES	NO	YES*
Thymidine kinase (EC 2.7.1.21)	4.30	NO	NO	YES*
Aspartokinase (EC 2.7.2.4)	-3.50	YES	NO	YES

3-oxoacyl-[ACP] synthase	2.26	NO	NO	YES*
hypothetical protein	3.26	NO	NO	YES*
Alkylphosphonate utilization operon protein PhnA	-3.38	NO	NO	YES
DNA helicase IV	4.62	NO	NO	YES*
General secretion pathway protein A / General secretion pathway protein B	3.10	NO	NO	YES*
ABC-type sugar transport system, periplasmic component	4.34	NO	NO	YES*
Protein sprT	3.19	NO	NO	YES*
DNA polymerase III chi subunit (EC 2.7.7.7)	2.16	NO	NO	YES*
Cytochrome c-type heme lyase subunit nrfF, nitrite reductase complex assembly	23.34	NO	NO	YES*
hypothetical protein	4.93	NO	NO	YES*
hypothetical protein	4.16	NO	NO	YES*
Putative response regulator	5.76	NO	NO	YES*
tRNA-(ms[2]io[6]A)-hydroxylase (EC 1.-.-.-)	4.79	NO	NO	YES*
hypothetical protein	3.89	NO	NO	YES*
Na+-driven multidrug efflux pump	3.82	NO	NO	YES*
OsmC/Ohr family protein	-3.65	NO	NO	YES
hypothetical protein	4.65	NO	NO	YES*
putative transcriptional regulator, XRE family	4.49	NO	NO	YES*
Oligopeptide transport ATP-binding protein OppF (TC 3.A.1.5.1)	7.92	NO	NO	YES*
Transcriptional regulator VpsT	5.11	YES	NO	YES*
Branched-chain amino acid transport ATP-binding protein LivF (TC 3.A.1.4.1)	5.54	NO	NO	YES*
hypothetical protein	4.46	NO	NO	YES*
hypothetical protein	8.84	NO	NO	YES*
hypothetical protein	4.46	NO	NO	YES*
Signal transduction histidine kinase	2.60	NO	NO	YES*
tRNA-specific adenosine-34 deaminase (EC 3.5.4.-)	2.10	NO	NO	YES*
Secreted trypsin-like serine protease	4.40	NO	NO	YES*
Metallo-beta-lactamase family protein, RNA-specific	4.20	NO	NO	YES*
Permease of the drug/metabolite transporter (DMT) superfamily	4.14	YES	NO	YES*
Methyl-accepting chemotaxis protein I (serine chemoreceptor protein)	3.66	NO	NO	YES*
hypothetical protein	4.52	NO	NO	YES*
DNA-binding response regulator	2.73	YES*	NO	YES*
Putative transporter	3.65	YES	NO	YES*
hypothetical protein	2.46	NO	NO	YES*
two-component system sensor protein	2.76	NO	NO	YES*
hypothetical protein	3.86	NO	NO	YES*

Heavy-metal-associated domain (N-terminus) and membrane-bounded cytochrome biog	3.97	NO	NO	YES*
Lipoprotein releasing system ATP-binding protein LolD	2.77	NO	NO	YES*
Transcriptional regulator	2.58	NO	NO	YES*
Transposase and inactivated derivatives	4.87	YES*	NO	YES*
hypothetical protein	4.21	NO	NO	YES*
hypothetical protein	3.18	NO	NO	YES*
Ornithine racemase (EC 5.1.1.12)	4.05	YES	NO	YES*
Transcriptional regulator, AraC family	5.46	NO	NO	YES*
membrane protein	-2.09	NO	NO	YES
hypothetical protein	2.99	NO	NO	YES*
hypothetical protein	3.75	NO	NO	YES*
Dihydroneopterin triphosphate pyrophosphohydolase type 2	3.14	YES*	NO	YES*
HD-domain protein	3.51	NO	NO	YES*
Chitin catabolic cascade sensor histidine kinase ChiS	2.51	NO	NO	YES*
hypothetical protein	2.87	NO	NO	YES*
Permease of the major facilitator superfamily	-3.78	NO	NO	YES
transposase and inactivated derivative	2.67	NO	NO	YES*
YjeF protein, function unknown	2.81	NO	NO	YES*
Quinolinate phosphoribosyltransferase [decarboxylating] (EC 2.4.2.19)	-2.61	NO	NO	YES
Putative analog of CcoH, COG3198	3.91	NO	NO	YES*
hypothetical protein	3.43	NO	NO	YES*
COG2357: Uncharacterized protein conserved in bacteria	4.44	NO	NO	YES*
hypothetical protein	2.90	NO	NO	YES*
DNA-binding response regulator, LuxR family	2.20	NO	NO	YES*
Menaquinone-specific isochorismate synthase (EC 5.4.4.2)	2.16	NO	NO	YES*
Transcriptional regulator, AraC family	2.78	NO	NO	YES*
transposase and inactivated derivative	2.63	NO	NO	YES*
UDP-2,3-diacylglucosamine hydrolase (EC 3.6.1.-)	2.82	YES	NO	YES*
ATPase involved in DNA repair	2.36	YES	NO	YES*
Signal transduction histidine kinase	7.71	YES	NO	YES*
hypothetical protein	3.51	NO	NO	YES*
hypothetical protein	-2.35	NO	NO	YES
Unsaturated fatty acid biosythesis repressor FabR, TetR family	-2.68	YES	NO	YES
Response regulator	4.06	NO	NO	YES*
Transcriptional regulator, MerR family	2.08	NO	NO	YES*
DNA mismatch repair endonuclease MutH	2.59	NO	NO	YES*
hypothetical protein	2.51	NO	NO	YES*

Putative MCP-type signal transduction protein	2.60	NO	NO	YES*
2-keto-4-pentenoate hydratase (EC 4.2.1.-)	-2.65	NO	NO	YES
transposase and inactivated derivative	2.31	NO	NO	YES*
hypothetical protein	3.27	NO	NO	YES
Aldehyde dehydrogenase (EC 1.2.1.3); Probable coniferyl aldehyde dehydrogenase	2.73	NO	NO	YES
Serine protein kinase (prkA protein), P-loop containing	8.30	NO	NO	YES
Glutamate Aspartate periplasmic binding protein precursor GltI (TC 3.A.1.3.4)	5.48	NO	NO	YES
hypothetical protein	2.35	YES	NO	YES
Flagellar protein FlgT	3.72	NO	NO	YES
Indole-3-glycerol phosphate synthase (EC 4.1.1.48) / Phosphoribosylanthranilate	2.78	NO	NO	YES
putative Glutathione-regulated potassium-efflux system protein KefB	2.26	NO	NO	YES
Thiamine kinase (EC 2.7.1.89) @ Adenosylcobinamide kinase (EC 2.7.1.156)	2.99	NO	NO	YES
rRNA small subunit methyltransferase I	2.06	NO	NO	YES
Histone acetyltransferase HPA2	6.42	NO	NO	YES
hypothetical protein	3.87	NO	NO	YES
hypothetical protein	7.06	YES	NO	YES
hypothetical protein	9.03	NO	NO	YES
Flagellar motor switch protein FliM	2.55	YES*	NO	YES
hypothetical protein	2.22	NO	NO	YES
hypothetical protein	6.18	NO	NO	YES
Glutamate synthase [NADPH] small chain (EC 1.4.1.13)	4.62	YES	NO	YES
pR99_vep58	3.63	NO	YES*	YES
Glutamine amidotransferases class-II	2.88	YES	NO	YES
General secretion pathway protein D	2.09	YES	NO	YES
hypothetical protein	3.69	NO	NO	YES
Outer membrane stress sensor protease DegQ, serine protease	2.11	NO	NO	YES
hypothetical protein	14.74	NO	NO	YES
O-succinylbenzoate-CoA synthase (EC 4.2.1.-)	2.45	NO	NO	YES
Permease of the major facilitator superfamily	2.92	NO	NO	YES
hypothetical protein	5.23	YES	NO	YES
L-xylulose 5-phosphate 3-epimerase (EC 5.1.3.-)	3.79	NO	NO	YES
Pyridoxamine 5'-phosphate oxidase-related, FMN-binding	5.87	YES	NO	YES
FIG067310: hypothetical protein	6.57	NO	NO	YES
RarD protein	7.04	YES	NO	YES
Peptide methionine sulfoxide reductase MsrA (EC 1.8.4.11) / Peptide methionine	2.29	NO	NO	YES

Putative two-component response regulatory protein	12.66	NO	NO	YES
hypothetical protein	4.61	NO	NO	YES
Endonuclease III (EC 4.2.99.18)	3.83	NO	NO	YES
Putative protein-S-isoprenylcysteine methyltransferase	4.96	NO	NO	YES
FIG004684: SpoVR-like protein	5.60	YES	NO	YES
Magnesium transporter	2.29	NO	NO	YES
ABC-type dipeptide transport system, periplasmic component	3.92	NO	NO	YES
Phosphosugar mutase of unknown sugar (see annotation)	2.52	NO	NO	YES
hypothetical protein	6.99	NO	NO	YES
Membrane protein YcjF	2.45	YES	NO	YES
Predicted ABC-type transport system, permease component	4.10	YES	NO	YES
Oligopeptide transport ATP-binding protein OppD (TC 3.A.1.5.1)	2.14	NO	NO	YES
Putative transcription antitermination protein NusG	15.58	NO	NO	YES
hypothetical protein	2.61	NO	NO	YES
hypothetical protein	5.21	YES	NO	YES
PTS system, N-acetylmuramic acid-specific IIB component (EC 2.7.1.69) / PTS sys	3.53	NO	NO	YES
Anti-anti-sigma regulatory factor	10.07	NO	NO	YES
hypothetical protein	2.24	NO	NO	YES
Uncharacterized protein, similar to the N-terminal domain of Lon protease	3.01	NO	NO	YES
Hypothetical protein, specific for Vibrio	4.87	NO	NO	YES
hypothetical protein	3.19	NO	NO	YES
RNA polymerase sigma-54 factor RpoN	3.81	YES*	NO	YES
Anaerobic glycerol-3-phosphate dehydrogenase subunit C (EC 1.1.5.3)	3.71	NO	NO	YES
ABC transporter ATP-binding protein YvcR	2.85	YES	NO	YES
hypothetical protein	3.62	YES	NO	YES
Ribosome recycling factor	4.12	NO	NO	YES
predicted 4-deoxy-L-threo-5-hexosulose-uronate ketol-isomerase (EC 5.3.1.17)	2.83	NO	NO	YES
Anaerobic glycerol-3-phosphate dehydrogenase subunit B (EC 1.1.5.3)	4.37	NO	NO	YES
hypothetical protein	3.10	NO	NO	YES
S-adenosyl-L-methionine dependent methyltransferase, similar to cyclopropane-fa	4.13	YES	NO	YES
FIG026291: Hypothetical periplasmic protein	3.92	NO	NO	YES
Nitrite reductase [NAD(P)H] small subunit (EC 1.7.1.4)	8.47	YES	NO	YES
Methyl-accepting chemotaxis protein	2.00	YES	NO	YES
pR99_vep56	4.50	NO	YES*	NO

pR99_vep59	3.02	NO	YES*	NO
pR99_vep05	3.25	YES	YES	NO
pR99_vep66	2.13	YES	YES	NO
pR99_traI	2.21	YES	YES	NO
pR99_vep20	2.85	YES	YES	NO
pR99_vep24	3.79	YES	YES	NO
hypothetical protein	-2.21	YES	NO	NO
NAD-dependent glyceraldehyde-3-phosphate dehydrogenase (EC 1.2.1.12)	2.66	YES*	NO	NO
54K polar flagellar sheath protein A	3.23	YES*	NO	NO
Flagellar hook-associated protein FlgK	8.99	YES*	NO	NO
Periplasmic nitrate reductase component NapE	2.16	YES*	NO	NO
N-acetylglucosamine-6-phosphate deacetylase (EC 3.5.1.25)	-2.20	YES	NO	NO
RNA polymerase sigma-70 factor, ECF subfamily	-3.10	YES	NO	NO
FIG106692: Outer membrane lipoprotein	3.00	YES*	NO	NO
Purine nucleotide synthesis repressor	-4.31	YES	NO	NO
Acyl-phosphate:glycerol-3-phosphate O-acyltransferase PlsY	3.31	YES*	NO	NO
Oligopeptide transport system permease protein OppC (TC 3.A.1.5.1)	2.82	YES*	NO	NO
Biotin-protein ligase (EC 6.3.4.15) / Biotin operon repressor	2.39	YES*	NO	NO
Fructose-1,6-bisphosphatase, GlpX type (EC 3.1.3.11)	-2.50	YES	NO	NO
Preprotein translocase subunit SecE (TC 3.A.5.1.1)	-2.99	YES	NO	NO
Adenylate kinase (EC 2.7.4.3)	-2.68	YES	NO	NO
S-adenosylmethionine synthetase (EC 2.5.1.6)	2.80	YES*	NO	NO
Flagellin protein FlaG	6.46	YES*	NO	NO
Putative cytoplasmic protein	-2.15	YES	NO	NO
Tol biopolymer transport system, TolR protein	2.74	YES*	NO	NO
Transcriptional activator RfaH	-2.86	YES	NO	NO
hypothetical protein	2.99	YES*	NO	NO
Flagellar basal-body P-ring formation protein FlgA	3.24	YES*	NO	NO
Protein YigP (COG3165) clustered with ubiquinone biosynthetic genes	2.02	YES*	NO	NO
Zn-dependent hydrolase (EC 3.-.-.-)	2.91	YES*	NO	NO
Phosphoserine phosphatase (EC 3.1.3.3)	3.26	YES*	NO	NO
Molybdenum ABC transporter, periplasmic molybdenum-binding protein ModA (TC 3.A	2.76	YES*	NO	NO
Glutamate racemase (EC 5.1.1.3)	2.18	YES*	NO	NO
Flagellar motor rotation protein MotA	3.59	YES*	NO	NO
Methyl-accepting chemotaxis protein	4.46	YES*	NO	NO

Flagellar synthesis regulator FleN	2.12	YES*	NO	NO
Outer membrane receptor protein	4.64	YES*	NO	NO
Transcriptional regulator, AraC family	4.15	YES*	NO	NO
Excinuclease ABC subunit C	3.28	YES*	NO	NO
hypothetical protein	2.81	YES	NO	NO
Protein-L-isoaspartate O-methyltransferase (EC 2.1.1.77)	2.85	YES	NO	NO
1-phosphofructokinase (EC 2.7.1.56)	2.46	YES	NO	NO
Signal transduction histidine kinase	3.61	YES	NO	NO
Probable Co/Zn/Cd efflux system membrane fusion protein	5.11	YES	NO	NO
Nitrate reductase cytochrome c550-type subunit	3.03	YES	NO	NO
hypothetical protein	5.96	YES	NO	NO
GGDEF and EAL domain proteins	2.58	YES	NO	NO
Uncharacterized iron-regulated protein	3.72	YES	NO	NO
hypothetical protein	3.73	YES	NO	NO
pR99_vep48	2.75	YES	NO	NO
hypothetical protein	3.24	YES	NO	NO
Predicted L-lactate dehydrogenase, Fe-S oxidoreductase subunit YkgE	5.01	YES	NO	NO
hypothetical protein	2.12	YES	NO	NO
Outer membrane receptor protein	3.66	YES	NO	NO
pR99_vep69	4.23	YES	NO	NO
FIG002076: hypothetical protein	6.91	YES	NO	NO
Transposase	-3.39	YES*	NO	NO
Predicted transcriptional regulator	3.56	YES	NO	NO
Response regulator	2.45	YES	NO	NO
Manganese superoxide dismutase (EC 1.15.1.1)	10.97	YES	NO	NO
COG1496: Uncharacterized conserved protein	6.45	YES	NO	NO
Transposase	2.54	YES	NO	NO
Periplasmic thiol:disulfide interchange protein DsbA	4.35	YES	NO	NO
Hydroxymethylglutaryl-CoA reductase (EC 1.1.1.34)	2.48	YES	NO	NO
Deoxyguanosinetriphosphate triphosphohydrolase (EC 3.1.5.1)	2.50	YES	NO	NO
5-nucleotidase SurE (EC 3.1.3.5)	2.38	YES	NO	NO
Para-aminobenzoate synthase, aminase component (EC 2.6.1.85)	3.56	YES	NO	NO
hypothetical protein	5.82	YES	NO	NO
transposase and inactivated derivative	2.80	YES	NO	NO
Adenylosuccinate synthetase (EC 6.3.4.4)	5.21	YES	NO	NO
hypothetical protein	4.65	YES	NO	NO
Putative regulator protein	2.58	YES	NO	NO
LSU ribosomal protein L31p	5.43	YES	NO	NO

RNA polymerase sigma factor SigZ	2.46	YES	NO	NO
hypothetical protein	7.24	YES	NO	NO
Transposase	2.84	YES	NO	NO
hypothetical protein	6.69	YES	NO	NO
CofC, F420 2-Phospho-l-lactate Guanylyltransferase	4.16	YES	NO	NO
3-oxoacyl-[ACP] reductase (EC 1.1.1.100)	2.37	YES	NO	NO
Putative response regulator	5.78	YES	NO	NO
hypothetical protein	6.09	YES	NO	NO
FIG139976: hypothetical protein	2.67	YES	NO	NO
Ribosomal-protein-S5p-alanine acetyltransferase	3.49	YES	NO	NO
Bacterioferritin	2.26	YES	NO	NO
Cobalt-zinc-cadmium resistance protein	2.75	YES	NO	NO
hypothetical protein	3.24	YES	NO	NO
Periplasmic nitrate reductase precursor (EC 1.7.99.4)	3.00	YES	NO	NO
Hypothetical protein, ydbT homolog	5.53	YES	NO	NO
FIGfam010717	3.04	YES	NO	NO
Aryl carrier domain	4.75	YES	NO	NO
hypothetical protein	4.38	YES	NO	NO
Bll6819 protein	8.60	NO	NO	NO
Mu-like prophage protein gp16	4.84	NO	NO	NO
Phosphate starvation-inducible ATPase PhoH with RNA binding motif	2.11	NO	NO	NO
Predicted ATP-dependent endonuclease of the OLD family	5.01	NO	NO	NO
HDIG domain protein	2.75	NO	NO	NO
Acetyltransferase (EC 2.3.1.-)	3.27	NO	NO	NO
Acetyltransferase	2.73	NO	NO	NO
Fumarylacetoacetase (EC 3.7.1.2)	5.30	NO	NO	NO
Glyoxylase family protein	3.57	NO	NO	NO
Inactive homolog of metal-dependent proteases, putative molecular chaperone	2.75	NO	NO	NO
Type II/IV secretion system ATPase TadZ/CpaE, associated with Flp pilus assembl	9.99	NO	NO	NO
Fimbrial protein pilin	3.22	NO	NO	NO
Leader peptidase (Prepilin peptidase) (EC 3.4.23.43) / N-methyltransferase (EC	4.35	NO	NO	NO
MSHA biogenesis protein MshK	2.14	NO	NO	NO
MSHA biogenesis protein MshO	4.16	NO	NO	NO
MSHA biogenesis protein MshP	4.25	NO	NO	NO
Acetyl-coenzyme A synthetase (EC 6.2.1.1)	19.67	NO	NO	NO
Possible sterol desaturase	3.71	NO	NO	NO
3-oxoacyl-[acyl-carrier-protein] synthase, KASIII (EC 2.3.1.41)	-2.29	NO	NO	NO
Betaine aldehyde dehydrogenase (EC	4.14	NO	NO	NO

1.2.1.8)				
Acetolactate synthase large subunit (EC 2.2.1.6)	2.11	NO	NO	NO
Phosphoserine phosphatase	9.79	NO	NO	NO
Uncharacterized domain COG3236 / GTP cyclohydrolase II (EC 3.5.4.25)	4.02	NO	NO	NO
3-dehydroquinate dehydratase II (EC 4.2.1.10)	-2.28	NO	NO	NO
Ribonucleotide reductase of class III (anaerobic), large subunit (EC 1.17.4.2)	-2.86	NO	NO	NO
1-hydroxy-2-methyl-2-(E)-butenyl 4-diphosphate synthase (EC 1.17.7.1)	2.70	NO	NO	NO
Molybdenum cofactor biosynthesis protein MoaC	2.21	NO	NO	NO
Thiamine biosynthesis protein thiI	-2.92	NO	NO	NO
Thiamin biosynthesis protein ThiC	5.01	NO	NO	NO
Cytochrome c-type protein NapC	3.28	NO	NO	NO
Putative cytochrome d ubiquinol oxidase subunit III (EC 1.10.3.-) (Cytochrome b	-2.38	NO	NO	NO
3-polyprenyl-4-hydroxybenzoate carboxy-lyase UbiX (EC 4.1.1.-)	2.07	NO	NO	NO
Homogentisate 1,2-dioxygenase (EC 1.13.11.5)	10.38	NO	NO	NO
3-hydroxyisobutyrate dehydrogenase (EC 1.1.1.31)	9.24	NO	NO	NO
Exodeoxyribonuclease I (EC 3.1.11.1)	3.04	NO	NO	NO
Glycogen debranching enzyme	12.81	NO	NO	NO
trypsin, putative	3.49	NO	NO	NO
Cell division transporter, ATP-binding protein FtsE (TC 3.A.5.1.1)	2.37	NO	NO	NO
Cell division protein FtsX	2.97	NO	NO	NO
Cell division protein FtsL	4.48	NO	NO	NO
Cell division protein BolA	-2.16	NO	NO	NO
DNA polymerase III subunits gamma and tau (EC 2.7.7.7)	2.05	NO	NO	NO
DNA polymerase III epsilon subunit (EC 2.7.7.7)	4.58	NO	NO	NO
DNA polymerase IV (EC 2.7.7.7)	2.35	NO	NO	NO
DNA polymerase III epsilon subunit (EC 2.7.7.7)	10.51	NO	NO	NO
TrkA, Potassium channel-family protein	2.41	NO	NO	NO
Potassium uptake protein TrkH	3.92	NO	NO	NO
Serine/threonine protein kinase PrkC, regulator of stationary phase	5.92	NO	NO	NO
Predicted hydrolase	3.13	NO	NO	NO
Predicted metal-dependent hydrolase with the TIM-barrel fold	2.40	NO	NO	NO
putative histidinol phosphatase and related hydrolases of the PHP family	2.11	NO	NO	NO
Cytoplasmic copper homeostasis protein cutC	4.24	NO	NO	NO

hypothetical protein	2.83	NO	NO	NO
hypothetical protein	4.21	NO	NO	NO
hypothetical protein	4.26	NO	NO	NO
hypothetical protein	2.17	NO	NO	NO
hypothetical protein	9.40	NO	NO	NO
hypothetical protein	16.23	NO	NO	NO
hypothetical protein	2.29	NO	NO	NO
hypothetical protein	2.39	NO	NO	NO
hypothetical protein	3.93	NO	NO	NO
hypothetical protein	3.16	NO	NO	NO
hypothetical protein	3.93	NO	NO	NO
hypothetical protein	2.60	NO	NO	NO
hypothetical protein	5.89	NO	NO	NO
hypothetical protein	19.94	NO	NO	NO
hypothetical protein	4.97	NO	NO	NO
hypothetical protein	8.33	NO	NO	NO
hypothetical protein	12.09	NO	NO	NO
hypothetical protein	3.40	NO	NO	NO
hypothetical protein	2.33	NO	NO	NO
hypothetical protein	5.54	NO	NO	NO
hypothetical protein	4.31	NO	NO	NO
hypothetical protein	4.43	NO	NO	NO
hypothetical protein	7.84	NO	NO	NO
hypothetical protein	3.15	NO	NO	NO
hypothetical protein	2.15	NO	NO	NO
hypothetical protein	2.33	NO	NO	NO
hypothetical protein	4.05	NO	NO	NO
hypothetical protein	4.77	NO	NO	NO
hypothetical protein	2.99	NO	NO	NO
hypothetical protein	2.79	NO	NO	NO
hypothetical protein	40.58	NO	NO	NO
hypothetical protein	5.82	NO	NO	NO
hypothetical protein	-3.26	NO	NO	NO
hypothetical protein	13.65	NO	NO	NO
hypothetical protein	2.43	NO	NO	NO
hypothetical protein	8.38	NO	NO	NO
hypothetical protein	4.60	NO	NO	NO
Hypothetical protein DUF454	5.47	NO	NO	NO
hypothetical protein	5.21	NO	NO	NO
hypothetical protein	2.44	NO	NO	NO
hypothetical protein	8.93	NO	NO	NO
hypothetical protein	4.91	NO	NO	NO
hypothetical protein	15.36	NO	NO	NO
hypothetical protein	11.52	NO	NO	NO
hypothetical protein	12.65	NO	NO	NO

hypothetical protein	5.43	NO	NO	NO
hypothetical protein	6.08	NO	NO	NO
hypothetical protein	9.42	NO	NO	NO
hypothetical protein	12.89	NO	NO	NO
hypothetical protein	3.29	NO	NO	NO
hypothetical protein	5.27	NO	NO	NO
hypothetical protein	3.90	NO	NO	NO
hypothetical protein	15.89	NO	NO	NO
hypothetical protein	2.34	NO	NO	NO
hypothetical protein	2.20	NO	NO	NO
hypothetical protein	5.05	NO	NO	NO
hypothetical protein	8.81	NO	NO	NO
hypothetical protein	7.03	NO	NO	NO
hypothetical protein	3.33	NO	NO	NO
hypothetical protein	-2.77	NO	NO	NO
hypothetical protein	16.65	NO	NO	NO
Uncharacterized conserved protein	5.99	NO	NO	NO
Uncharacterized protein conserved in bacteria	-5.25	NO	NO	NO
Uncharacterized protein conserved in bacteria	2.53	NO	NO	NO
Transposase	2.14	NO	NO	NO
Transposase and inactivated derivatives	4.20	NO	NO	NO
transposase and inactivated derivative	2.19	NO	NO	NO
transposase and inactivated derivative	2.84	NO	NO	NO
ISBma1, transposase	2.90	NO	NO	NO
transposase and inactivated derivative	2.26	NO	NO	NO
Transposase and inactivated derivatives	2.92	NO	NO	NO
Transposase and inactivated derivatives	3.37	NO	NO	NO
ISBma1, transposase	3.08	NO	NO	NO
transposase and inactivated derivative	2.98	NO	NO	NO
transposase and inactivated derivative	2.47	NO	NO	NO
Iron-sulfur cluster-binding protein	2.80	NO	NO	NO
7,8-didemethyl-8-hydroxy-5-deazariboflavin synthase subunit 2	11.18	NO	NO	NO
ATP-dependent DNA ligase	2.35	NO	NO	NO
Ubiquitin-protein ligase	4.40	NO	NO	NO
Alkaline phosphatase (EC 3.1.3.1)	6.73	NO	NO	NO
Glycerate kinase (EC 2.7.1.31)	2.98	NO	NO	NO
Maleylacetoacetate isomerase (EC 5.2.1.2) @ Glutathione S-transferase, zeta (EC	4.92	NO	NO	NO
Kynurenine 3-monooxygenase (EC 1.14.13.9)	5.47	NO	NO	NO
Aspartate/tyrosine/aromatic aminotransferase	8.53	NO	NO	NO
Methylcrotonyl-CoA carboxylase carboxyl transferase subunit (EC 6.4.1.4)	6.90	NO	NO	NO

Glucosamine kinase GpsK (EC 2.7.1.8)	11.07	NO	NO	NO
5-Enolpyruvylshikimate-3-phosphate synthase (EC 2.5.1.19)	2.37	NO	NO	NO
PrpF protein involved in 2-methylcitrate cycle	11.90	NO	NO	NO
Isocitrate lyase (EC 4.1.3.1)	4.36	NO	NO	NO
Beta-galactosidase/beta-glucuronidase	2.82	NO	NO	NO
Beta-galactosidase (EC 3.2.1.23) / Beta-glucosidase/6-phospho-beta-glucosidase	8.38	NO	NO	NO
Mannose-6-phosphate isomerase (EC 5.3.1.8)	7.62	NO	NO	NO
Glycosidase	4.38	NO	NO	NO
Malate synthase-related protein	3.62	NO	NO	NO
Mannose-6-phosphate isomerase (EC 5.3.1.8)	9.32	NO	NO	NO
Galactose-1-phosphate uridylyltransferase (EC 2.7.7.10)	4.92	NO	NO	NO
Aldose 1-epimerase (EC 5.1.3.3)	7.62	NO	NO	NO
NAD-specific glutamate dehydrogenase (EC 1.4.1.2), large form	4.16	NO	NO	NO
Lactoylglutathione lyase	3.62	NO	NO	NO
Guanylate kinase (EC 2.7.4.8)	-3.63	NO	NO	NO
Myo-inositol 2-dehydrogenase (EC 1.1.1.18)	8.25	NO	NO	NO
D-lactate dehydrogenase (EC 1.1.1.28)	2.69	NO	NO	NO
Evolved beta-D-galactosidase, alpha subunit	9.19	NO	NO	NO
Lipase precursor (EC 3.1.1.3)	5.25	NO	NO	NO
Lipase chaperone	7.98	NO	NO	NO
3-hydroxydecanoyl-[ACP] dehydratase (EC 4.2.1.60)	3.30	NO	NO	NO
Glycerophosphoryl diester phosphodiesterase (EC 3.1.4.46)	3.10	NO	NO	NO
[Protein-PII] uridylyltransferase (EC 2.7.7.59)	3.43	NO	NO	NO
Dihydroorotate dehydrogenase (EC 1.3.3.1)	-3.34	NO	NO	NO
Queuosine biosynthesis QueD, PTPS-I	-5.79	NO	NO	NO
GMP synthase [glutamine-hydrolyzing] (EC 6.3.5.2)	-3.15	NO	NO	NO
Beta-N-acetylhexosaminidase, (GlcNAc)2 catabolism	6.64	NO	NO	NO
Glucosamine-link cellobiase (EC 3.2.1.21)	12.67	NO	NO	NO
Tryptophanase (EC 4.1.99.1)	7.80	NO	NO	NO
tRNA-guanine transglycosylase (EC 2.4.2.29)	-3.11	NO	NO	NO
tRNA nucleotidyltransferase (EC 2.7.7.21) (EC 2.7.7.25)	3.52	NO	NO	NO
Methionyl-tRNA formyltransferase (EC 2.1.2.9)	7.47	NO	NO	NO
Flagellar P-ring protein FlgI	11.88	NO	NO	NO
Flagellin protein FlaF	3.97	NO	NO	NO

Flagellar sensor histidine kinase FleS	3.14	NO	NO	NO
Flagellar regulatory protein FleQ	4.08	NO	NO	NO
Flagellar M-ring protein FliF	3.59	NO	NO	NO
Flagellar hook-associated protein FlgK	16.04	NO	NO	NO
Methyl-accepting chemotaxis protein I (serine chemoreceptor protein)	4.57	NO	NO	NO
Methyl-accepting chemotaxis protein II (mcp-II) (aspartate chemoreceptor protei	2.87	NO	NO	NO
Methyl-accepting chemotaxis protein	2.49	NO	NO	NO
Positive regulator of CheA protein activity (CheW)	-2.39	NO	NO	NO
Methyl-accepting chemotaxis protein I (serine chemoreceptor protein)	11.23	NO	NO	NO
FOG: CheY-like receiver	4.42	NO	NO	NO
Methyl-accepting chemotaxis protein	6.12	NO	NO	NO
Chemotaxis regulator - transmits chemoreceptor signals to flagelllar motor comp	5.79	NO	NO	NO
Methyl-accepting chemotaxis protein	4.28	NO	NO	NO
Chemotactic transducer-related protein	3.19	NO	NO	NO
Methyl-accepting chemotaxis protein	15.49	NO	NO	NO
Methyl-accepting chemotaxis protein I (serine chemoreceptor protein)	7.32	NO	NO	NO
Protoporphyrinogen IX oxidase, oxygen-independent, HemG (EC 1.3.-.-)	3.29	NO	NO	NO
Putative oxidoreductase SMc00968	7.08	NO	NO	NO
Cell wall-associated hydrolase	-2.16	NO	NO	NO
Rare lipoprotein A precursor	2.55	NO	NO	NO
Transglycosylase, Slt family	3.01	NO	NO	NO
Proposed peptidoglycan lipid II flippase MurJ	2.63	NO	NO	NO
Phospho-N-acetylmuramoyl-pentapeptide-transferase (EC 2.7.8.13)	-2.37	NO	NO	NO
Lipoprotein nlpI precursor	-2.94	NO	NO	NO
Membrane-bound lytic murein transglycosylase D precursor (EC 3.2.1.-)	3.25	NO	NO	NO
Secreted trypsin-like serine protease	13.54	NO	NO	NO
FIG000906: Predicted Permease	2.07	NO	NO	NO
Permeases of the major facilitator superfamily	3.57	NO	NO	NO
Permease of the major facilitator superfamily	6.41	NO	NO	NO
Permease of the major facilitator superfamily	4.44	NO	NO	NO
Permease of the major facilitator superfamily	3.21	NO	NO	NO
pR99_vep04	4.23	NO	NO	NO
pR99_vep19	4.55	NO	NO	NO
pR99_vep31	6.55	NO	NO	NO
pR99_vep70	5.02	NO	NO	NO

Hypothetical protein in cluster with HutR, VCA0066 homolog	6.06	NO	NO	NO
PTS system, beta-glucoside-specific IIB component (EC 2.7.1.69) / PTS system, b	2.41	NO	NO	NO
Fructose-specific phosphocarrier protein HPr (EC 2.7.1.69) / PTS system, fructo	2.84	NO	NO	NO
D-alanyl-D-alanine carboxypeptidase (EC 3.4.16.4)	2.53	NO	NO	NO
FOG: TPR repeat protein	-2.66	NO	NO	NO
Guanylate cyclase-related protein	5.20	NO	NO	NO
Predicted hydrolase of the metallo-beta-lactamase superfamily, clustered with K	7.86	NO	NO	NO
Ribosomal RNA large subunit methyltransferase N (EC 2.1.1.-)	-2.89	NO	NO	NO
Putative cytoplasmic protein	3.36	NO	NO	NO
putative cytoplasmic protein	2.21	NO	NO	NO
Protein of unknown function DUF81	2.43	NO	NO	NO
membrane protein	2.50	NO	NO	NO
membrane protein	5.34	NO	NO	NO
membrane protein	5.74	NO	NO	NO
CONSERVED MEMBRANE PROTEIN	2.27	NO	NO	NO
BatD	4.94	NO	NO	NO
Ribosomal large subunit pseudouridine synthase D (EC 4.2.1.70)	2.80	NO	NO	NO
LSU ribosomal protein L27p	-3.07	NO	NO	NO
Ribosomal-protein-S18p-alanine acetyltransferase (EC 2.3.1.-)	3.81	NO	NO	NO
FIG027190: Putative transmembrane protein	3.26	NO	NO	NO
ATPase of the AAA+ class	-2.19	NO	NO	NO
putative	3.53	NO	NO	NO
S-ribosylhomocysteine lyase (EC 4.4.1.21) / Autoinducer-2 production protein Lu	-3.61	NO	NO	NO
Regulatory protein RecX	2.62	NO	NO	NO
ATP-dependent DNA helicase RecG (EC 3.6.1.-)	5.79	NO	NO	NO
Oxidoreductase, short-chain dehydrogenase/reductase family (EC 1.1.1.-)	16.77	NO	NO	NO
Paraquat-inducible protein B	4.01	NO	NO	NO
Poly(A) polymerase (EC 2.7.7.19)	3.42	NO	NO	NO
C4-dicarboxylate transport transcriptional regulatory protein	20.73	NO	NO	NO
C4-dicarboxylate transport transcriptional regulatory protein	2.92	NO	NO	NO
Anti-anti-sigma regulatory factor	4.89	NO	NO	NO
Response regulator	4.14	NO	NO	NO
HTH-type transcriptional regulator BetI	2.95	NO	NO	NO
Two component response regulator	10.52	NO	NO	NO
Transcriptional regulator	2.63	NO	NO	NO

Putative transcriptional regulator	4.10	NO	NO	NO
Transcriptional regulator, AraC family	3.17	NO	NO	NO
Transcriptional regulator, AraC family	2.67	NO	NO	NO
Transcriptional regulatory protein CitB, DpiA	8.33	NO	NO	NO
Sensor kinase CitA, DpiB (EC 2.7.3.-)	2.82	NO	NO	NO
cAMP-binding proteins - catabolite gene activator and regulatory subunit of cAM	4.97	NO	NO	NO
FOG: EAL domain protein	2.19	NO	NO	NO
FOG: EAL domain protein	3.73	NO	NO	NO
FOG: EAL domain protein	3.89	NO	NO	NO
GGDEF domain protein	6.17	NO	NO	NO
GGDEF family protein	4.37	NO	NO	NO
GGDEF family protein	3.22	NO	NO	NO
GGDEF family protein	2.09	NO	NO	NO
Extracellular solute-binding protein, family 3/GGDEF domain protein	3.53	NO	NO	NO
GGDEF family protein	3.07	NO	NO	NO
Transcriptional regulator, LysR family	3.09	NO	NO	NO
Transcriptional regulator, LysR family	3.93	NO	NO	NO
Probable transcriptional activator for leuABCD operon	5.76	NO	NO	NO
Probable transcriptional activator for leuABCD operon	5.40	NO	NO	NO
Transcriptional regulator, MarR family	6.56	NO	NO	NO
Putative HTH-type transcriptional regulator ybaO	5.15	NO	NO	NO
Error-prone repair protein UmuD	2.09	NO	NO	NO
ATPase involved in DNA repair	-2.40	NO	NO	NO
Methylated-DNA--protein-cysteine methyltransferase (EC 2.1.1.63)	3.66	NO	NO	NO
Exodeoxyribonuclease V alpha chain (EC 3.1.11.5) ## RecD	3.01	NO	NO	NO
Predicted endonuclease distantly related to archaeal Holliday junction resolvas	2.29	NO	NO	NO
Helicase	3.38	NO	NO	NO
Transcriptional regulator, LacI family	5.32	NO	NO	NO
Fusaric acid resistance protein fusE	9.53	NO	NO	NO
Predicted exporter of the RND superfamily	4.56	NO	NO	NO
MFS family multidrug transport protein, bicyclomycin resistance protein	2.84	NO	NO	NO
Multidrug resistance protein 2	2.42	NO	NO	NO
Na+-driven multidrug efflux pump	3.36	NO	NO	NO
Permease of the drug/metabolite transporter (DMT) superfamily	11.53	NO	NO	NO
Permease of the drug/metabolite transporter (DMT) superfamily	2.31	NO	NO	NO
Glutathione S-transferase, omega (EC 2.5.1.18)	2.38	NO	NO	NO

Heat shock protein HslJ	-2.69	NO	NO	NO
Fumarate and nitrate reduction regulatory protein	3.95	NO	NO	NO
Polyferredoxin NapH (periplasmic nitrate reductase)	3.92	NO	NO	NO
Ferredoxin-type protein NapG (periplasmic nitrate reductase)	2.82	NO	NO	NO
Periplasmic protein torT precursor	4.22	NO	NO	NO
HflK protein	7.14	NO	NO	NO
DNA primase (EC 2.7.7.-)	2.37	NO	NO	NO
Ribonuclease E inhibitor RraB	-2.77	NO	NO	NO
General secretion pathway protein H	2.93	NO	NO	NO
Chromosome partition protein MukF	2.26	NO	NO	NO
Chromosome segregation ATPase	6.94	NO	NO	NO
Sensor histidine kinase	2.16	NO	NO	NO
Signal transduction histidine kinase	3.81	NO	NO	NO
Similarity with glutathionylspermidine synthase (EC 6.3.1.8), group 1	10.15	NO	NO	NO
FIG002708: Protein SirB1	2.30	NO	NO	NO
conserved protein of unknown function; putative YcgN protein	13.57	NO	NO	NO
Excinuclease ABC, C subunit-like	4.53	NO	NO	NO
FIG003276: zinc-binding protein	5.37	NO	NO	NO
nonspecific acid phosphatase precursor	5.42	NO	NO	NO
Metallo-beta-lactamase superfamily protein PA0057	6.27	NO	NO	NO
Membrane-fusion protein	3.06	NO	NO	NO
Uncharacterized protein YtfM precursor	6.17	NO	NO	NO
Uncharacterized protein, probably surface-located	6.41	NO	NO	NO
Preprotein translocase subunit YajC (TC 3.A.5.1.1)	-3.69	NO	NO	NO
Molybdopterin-guanine dinucleotide biosynthesis protein MobB / Molybdopterin bi	2.84	NO	NO	NO
Translation initiation factor 1	3.51	NO	NO	NO
Glucose-1-phosphate adenylyltransferase (EC 2.7.7.27)	4.30	NO	NO	NO
Phosphoglycerate transport system transcriptional regulatory protein PgtA	9.53	NO	NO	NO
CcdA protein (antitoxin to CcdB)	3.83	NO	NO	NO
Ferritin-like protein 2	-2.10	NO	NO	NO
Anthranilate synthase, amidotransferase component (EC 4.1.3.27)	2.25	NO	NO	NO
TonB-dependent receptor	4.17	NO	NO	NO
TonB-dependent receptor	4.30	NO	NO	NO
TonB system biopolymer transport component; Chromosome segregation ATPase	12.19	NO	NO	NO

Biopolymer transport protein ExbD/TolR	8.01	NO	NO	NO
Ferric iron ABC transporter, ATP-binding protein	2.58	NO	NO	NO
Outer membrane protein SypB	5.21	NO	NO	NO
Sugar transferase SypR involved in lipopolysaccharide synthesis	15.83	NO	NO	NO
Anti anti-sigma regulatory factor SypA	12.17	NO	NO	NO
Lipid A biosynthesis lauroyl acyltransferase (EC 2.3.1.-)	2.69	NO	NO	NO
Phosphoheptose isomerase (EC 5.3.1.-)	2.09	NO	NO	NO
UDP-glucose 4-epimerase (EC 5.1.3.2)	4.26	NO	NO	NO
3-deoxy-D-manno-octulosonate 8-phosphate phosphatase (EC 3.1.3.45)	2.46	NO	NO	NO
Undecaprenyl pyrophosphate synthetase (EC 2.5.1.31)	2.16	NO	NO	NO
LptA, protein essential for LPS transport across the periplasm	2.28	NO	NO	NO
Chitinase (EC 3.2.1.14)	8.85	NO	NO	NO
RTX toxin	7.17	NO	NO	NO
Hemolysins and related proteins containing CBS domains	3.14	NO	NO	NO
Bacillosamine/Legionaminic acid biosynthesis aminotransferase PglE; 4-keto-6-de	2.00	NO	NO	NO
Histone acetyltransferase HPA2	-4.97	NO	NO	NO
Sensor histidine kinase	4.21	NO	NO	NO
ABC transporter substrate-binding protein	4.15	NO	NO	NO
Methionine ABC transporter ATP-binding protein	2.53	NO	NO	NO
ABC-type amino acid transport, signal transduction systems, periplasmic compone	6.19	NO	NO	NO
ABC transporter ATP-binding protein YvcR	3.44	NO	NO	NO
ABC-type uncharacterized transport system, permease component	7.27	NO	NO	NO
ABC-type sulfate transport system, permease component	8.17	NO	NO	NO
ABC-type amino acid transport, signal transduction systems, periplasmic compone	6.63	NO	NO	NO
Phosphate transport system permease protein PstA (TC 3.A.1.7.1)	6.61	NO	NO	NO
ABC-type protease/lipase transport system, ATPase and permease component	5.50	NO	NO	NO
Ribose ABC transport system, permease protein RbsC (TC 3.A.1.2.1)	3.45	NO	NO	NO
Ribose ABC transport system, periplasmic ribose-binding protein RbsB (TC 3.A.1.	6.60	NO	NO	NO
ABC-type sugar transport system, ATPase component	7.07	NO	NO	NO
ABC-type amino acid transport/signal transduction systems, periplasmic componen	13.81	NO	NO	NO

ABC-type uncharacterized transport system, periplasmic component	4.88	NO	NO	NO
Chromate transport protein ChrA	5.51	NO	NO	NO
Molybdenum transport ATP-binding protein ModC (TC 3.A.1.8.1)	2.71	NO	NO	NO
Molybdenum transport system permease protein ModB (TC 3.A.1.8.1)	3.39	NO	NO	NO
(GlcNAc)2 ABC transporter, ATP-binding component 1	9.99	NO	NO	NO
TolA protein	2.28	NO	NO	NO
Cobalt-zinc-cadmium resistance protein CzcA; Cation efflux system protein CusA	15.10	NO	NO	NO
TRAP dicarboxylate transporter, DctQ subunit, unknown substrate 3	6.80	NO	NO	NO
Transport ATP-binding protein CydD	2.80	NO	NO	NO
Potassium voltage-gated channel subfamily KQT; possible potassium channel, VIC	4.19	NO	NO	NO
TRAP-type C4-dicarboxylate transport system, large permease component	7.19	NO	NO	NO
TRAP transporter solute receptor, unknown substrate 6	5.05	NO	NO	NO
TRAP transporter solute receptor, TAXI family precursor	13.46	NO	NO	NO
TRAP-type transport system, small permease component, predicted N-acetylneurami	11.45	NO	NO	NO
Probable low-affinity inorganic phosphate transporter	-2.04	NO	NO	NO
Nucleoside permease NupC	8.09	NO	NO	NO
Oligopeptide transport system permease protein OppB (TC 3.A.1.5.1)	3.14	NO	NO	NO
Fucose permease	2.87	NO	NO	NO
Potassium channel protein	15.76	NO	NO	NO
Na+/H+ antiporter NhaC	10.99	NO	NO	NO
sodium-solute symporter, putative	12.23	NO	NO	NO
DNA-binding protein HU-beta	-2.70	NO	NO	NO
