# Supplementary material for: The Effect of the Environmental Temperature on the Adaptation to Host in the Zoonotic Pathogen Vibrio vulnificus
Source: Front Microbiol. 2020 Mar 27;11:489. doi: 10.3389/fmicb.2020.00489 (PMC7137831; doi:10.3389/fmicb.2020.00489)
Supplement: TABLE S5 — V. vulnificus R99 strain temperature stimulon. The temperature stimulon consists on the set of DEGs by V. vulnificus in response to an increase in temperature. The value of fold change per gene at each infective temperature (25, 28, and 37°C vs. non-infective temperature [20°C]) is shown. ∗: only genes with values of fold change −2 ≤ X ≤ 2 with a p-value cut-off of 0.05 considered. +: gene upregulated at infective temperature; −: gene downregulated in at infective temperature. NS, non-significant. [file Data_Sheet_5.PDF]

Table S5. *V. vulnificus* R99 strain temperature stimulon. The temperature stimulon consists on the set of DEGs by *V. vulnificus* in response to an increase in temperature. The value of fold change per gene at each infective temperature (25, 28 and 37ºC vs non-infective temperature [20ºC]) is shown.

*: only genes with values of fold change -2≤X≤2 with a p-value cut-off of 0.05 considered. +: gene upregulated at infective temperature; -: gene downregulated in at infective temperature. NS: non-significant.

Gene	25ºC	28ºC	37ºC
sensor histidine kinase	17.633621	NS	NS
Enoyl-CoA hydratase (EC 4.2.1.17) / Delta(3)-cis-delta(2)-trans-enoyl-CoA isome	15.036013	NS	NS
Predicted deacylase	13.650302	NS	NS
Predicted ATP-dependent endonuclease of the OLD family	12.293001	NS	5.008788
2,4-dienoyl-CoA reductase [NADPH] (EC 1.3.1.34)	11.301286	NS	NS
Peptide ABC transporter, ATP-binding protein	10.3776245	NS	NS
Methyl-accepting chemotaxis protein	8.530206	NS	4.280818
Branched-chain amino acid aminotransferase (EC 2.6.1.42)	7.780031	NS	NS
Sodium-dependent transporter	7.691552	NS	NS
Phosphate starvation-inducible ATPase PhoH with RNA binding motif	7.1379533	NS	2.105939
hypothetical protein	7.0342674	NS	NS
Predicted polymerase	6.3766685	NS	NS
Anthranilate phosphoribosyltransferase (EC 2.4.2.18)	6.076541	NS	NS
C4-dicarboxylate transport transcriptional regulatory protein	5.9864335	NS	2.924044
3-ketoacyl-CoA thiolase (EC 2.3.1.16) @ Acetyl-CoA acetyltransferase (EC 2.3.1.	5.8579407	NS	NS
Methyl-accepting chemotaxis protein I (serine chemoreceptor protein)	5.6527376	NS	4.566214
CDP-diacylglycerol--serine O-phosphatidyltransferase (EC 2.7.8.8)	5.4738417	NS	NS
Transglycosylase, Slt family	5.471669	NS	3.01175
hypothetical protein	5.3167324	NS	3.509237
Transporter, putative	5.2565365	NS	NS
Exodeoxyribonuclease I (EC 3.1.11.1)	5.1237063	NS	3.039368
Ornithine carbamoyltransferase (EC 2.1.3.3)	5.007048	NS	NS
D-3-phosphoglycerate dehydrogenase (EC 1.1.1.95)	4.9785595	NS	NS
hypothetical protein	4.9400783	NS	3.726937
TRAP transporter solute receptor, unknown substrate 6	4.7969832	NS	5.045959
GGDEF and EAL domain proteins	4.783554	NS	2.578629
Alpha-amylase (EC 3.2.1.1)	4.645408	NS	NS
Anaerobic glycerol-3-phosphate dehydrogenase subunit B (EC 1.1.5.3)	4.610371	NS	4.369078

Formate dehydrogenase subunit or accessory protein	4.363587	NS	NS
FIGfam010717	4.303573	NS	3.040891
Protoporphyrinogen IX oxidase, oxygen-independent, HemG (EC 1.3.-.-)	4.276845	NS	3.285215
Di-and tricarboxylate transporter	4.1870294	NS	NS
Deoxyribodipyrimidine photolyase (EC 4.1.99.3)	4.1759377	NS	NS
Multidrug resistance transporter, Bcr/CflA family	4.1274476	NS	NS
Allophanate hydrolase 2 subunit 1 (EC 3.5.1.54)	4.1056437	NS	NS
Glyoxylase family protein	4.063802	NS	3.565259
Periplasmic alpha-amylase (EC 3.2.1.1)	4.0228124	NS	NS
Predicted permease	4.011177	NS	NS
Outer membrane protein Imp, required for envelope biogenesis / Organic solvent	3.9775214	NS	NS
Indole-3-glycerol phosphate synthase (EC 4.1.1.48) / Phosphoribosylanthranilate	3.974333	NS	2.782499
Dipeptide-binding ABC transporter, periplasmic substrate-binding component (TC	3.8697708	NS	NS
Cystathionine beta-lyase (EC 4.4.1.8)	3.7736626	NS	NS
Arginine deiminase (EC 3.5.3.6)	3.7351062	NS	2.65863
putative Glutathione-regulated potassium-efflux system protein KefB	3.6775665	NS	2.262733
Ferric iron ABC transporter, ATP-binding protein	3.6646733	NS	2.584985
Phosphosugar mutase of unknown sugar (see annotation)	3.6364658	NS	2.5243
hypothetical protein	3.5998235	NS	10.43589
COG1720: Uncharacterized conserved protein	3.525201	NS	NS
Ribosomal-protein-S5p-alanine acetyltransferase	3.4802496	NS	3.490994
Cysteine synthase B (EC 2.5.1.47)	3.4168298	NS	NS
Acetolactate synthase large subunit (EC 2.2.1.6)	3.4111426	NS	2.108565
Glutathione-regulated potassium-efflux system ancillary protein KefG	3.3490748	NS	NS
hypothetical protein	3.3419077	NS	4.96521
3-hydroxydecanoyl-[ACP] dehydratase (EC 4.2.1.60)	3.3287716	NS	3.299426
Long-chain fatty acid transport protein	3.3124068	NS	NS
Predicted metal-dependent hydrolase with the TIM-barrel fold	3.2129762	NS	2.401689
DNA helicase IV	3.2085247	NS	4.621372
Predicted signal transduction protein	3.197946	NS	NS
hypothetical protein	3.1767373	NS	4.429771
Response regulator	3.1166224	NS	2.451299
2-dehydropantoate 2-reductase (EC 1.1.1.169)	3.1098208	NS	NS
hypothetical protein	3.1077037	NS	NS
Membrane-bound lytic murein transglycosylase D precursor (EC 3.2.1.-)	3.1050043	NS	3.246029
Nitrogen regulation protein NR(I)	3.0582986	NS	NS
Predicted L-lactate dehydrogenase, Fe-S oxidoreductase subunit YkgE	3.05424	NS	5.009349

Cytochrome c-type biogenesis protein DsbD, protein-disulfide reductase (EC 1.8.	3.0197127	NS	NS
S-(hydroxymethyl)glutathione dehydrogenase (EC 1.1.1.284)	3.018746	2.283796	NS
Predicted membrane-associated metal-dependent hydrolase	3.0101163	NS	NS
Cell division protein FtsK	3.0061727	NS	NS
diguanylate cyclase (GGDEF domain) with PAS/PAC sensor	3.0017948	NS	NS
Vulnibactin utilization protein VuuB	2.9877326	NS	NS
FIG139976: hypothetical protein	2.9803486	NS	2.672751
Glutamate synthase [NADPH] small chain (EC 1.4.1.13)	2.9760196	NS	4.622984
DinG family ATP-dependent helicase YoaA	2.9626977	NS	NS
membrane protein	2.9564452	NS	NS
ABC-type multidrug transport system, ATPase component	2.9395044	NS	NS
ABC-type protease exporter, membrane fusion protein (MFP) family component PrtE	2.922912	NS	NS
hypothetical protein	2.8871713	NS	NS
oxidoreductase, short-chain dehydrogenase/reductase family	2.8541143	NS	NS
Pyruvate kinase (EC 2.7.1.40)	2.8378842	NS	NS
Catalase (EC 1.11.1.6) / Peroxidase (EC 1.11.1.7)	2.832722	NS	NS
Methyl-accepting chemotaxis protein	2.8225641	NS	NS
Alkyl hydroperoxide reductase protein F (EC 1.6.4.-)	2.8160775	NS	NS
pR99_vep20	2.8155577	2.049634	2.849567
Predicted P-loop ATPase fused to an acetyltransferase COG1444	2.8155577	NS	NS
Transport ATP-binding protein CydD	2.8104684	NS	2.798192
Dephospho-CoA kinase (EC 2.7.1.24)	2.8076005	NS	NS
Pole remodelling regulatory diguanylate cyclase	2.7947526	NS	NS
Predicted endonuclease distantly related to archaeal Holliday junction resolvas	2.7529902	NS	2.290208
Aldehyde dehydrogenase (EC 1.2.1.3); Probable coniferyl aldehyde dehydrogenase	2.7519772	NS	2.734248
Glutamate-ammonia-ligase adenylyltransferase (EC 2.7.7.42)	2.7349985	NS	NS
ABC-type dipeptide transport system, periplasmic component	2.7248597	NS	3.924421
Putative oxidoreductase YncB	2.7213526	NS	NS
MSHA biogenesis protein MshH	2.7196867	NS	NS
Deoxyguanosinetriphosphate triphosphohydrolase (EC 3.1.5.1)	2.715066	NS	2.495501
Histidinol dehydrogenase (EC 1.1.1.23)	2.713716	NS	NS
Glutathione S-transferase (EC 2.5.1.18)	2.7067754	2.339893	NS
Hydrolase, alpha/beta fold family functionally coupled to Phosphoribulokinase	2.6883676	NS	NS
Lipopolysaccharide heptosyltransferase I (EC	2.6785324	NS	NS

2.4.1.-)			
Molybdopterin-guanine dinucleotide biosynthesis protein MobB / Molybdopterin bi	2.6780012	NS	2.841867
ABC-type antimicrobial peptide transport system, permease component	2.6726508	NS	NS
Universal stress protein A	2.6659405	NS	NS
Membrane fusion component of tripartite multidrug resistance system	2.6581724	NS	NS
Autoinducer 2-binding periplasmic protein LuxP precursor	2.6471848	NS	NS
hypothetical protein	2.6447964	NS	NS
Conserved protein YcjX with nucleoside triphosphate hydrolase domain	2.6413398	NS	NS
Periplasmic nitrate reductase precursor (EC 1.7.99.4)	2.635737	NS	3.001851
Na+/H+ antiporter, putative	2.5972867	-2.01755	NS
Endonuclease III (EC 4.2.99.18)	2.5753593	NS	3.826308
Transcriptional regulator, TetR family	2.5723367	NS	NS
Menaquinone-specific isochorismate synthase (EC 5.4.4.2)	2.5678172	NS	2.160179
Succinylglutamic semialdehyde dehydrogenase (EC 1.2.1.71)	2.5545	NS	NS
RNA polymerase sigma factor RpoS	2.5483637	NS	NS
DNA mismatch repair protein MutS	2.524442	NS	NS
Uncharacterized protein YtfM precursor	2.5207975	NS	NS
Acetolactate synthase small subunit (EC 2.2.1.6)	2.5078824	NS	NS
Protein ydjA	2.5049582	NS	2.923801
Aspartate aminotransferase (AspB-4) (EC 2.6.1.1)	2.4919877	NS	NS
PQQ-dependent oxidoreductase, gdhB family	2.481172	NS	NS
Transcriptional regulator, LysR family	2.4649072	NS	NS
Lipoprotein releasing system ATP-binding protein LolD	2.447621	NS	2.772184
3-oxoacyl-[ACP] reductase (EC 1.1.1.100)	2.4440184	NS	2.369528
LppC putative lipoprotein	2.4319096	-2.19902	NS
Flagellar regulatory protein FleQ	2.4312468	NS	4.083206
Arylesterase precursor (EC 3.1.1.2)	2.4074624	NS	NS
ABC-type dipeptide transport system, periplasmic component	2.4063435	NS	NS
hypothetical protein	2.4048078	NS	3.860833
C4-dicarboxylate transporter	2.4021306	NS	NS
Methyl-accepting chemotaxis protein II (mcp-II) (aspartate chemoreceptor protei	2.3932016	NS	2.874289
hypothetical protein	2.3894882	NS	2.11782
Predicted transcriptional regulator of pyridoxine metabolism	2.3754983	NS	NS
FIG023406: hypothetical protein	2.3747501	NS	8.407999
UDP-N-acetylmuramoylalanine--D-glutamate ligase (EC 6.3.2.9)	2.3646564	NS	NS
NAD-dependent malic enzyme (EC 1.1.1.38)	2.3631244	NS	NS

DNA-directed RNA polymerase specialized sigma subunit	2.3616438	NS	NS
Chaperone protein HscA	2.3612194	NS	NS
hypothetical protein	2.3611825	NS	NS
GNAT family acetyltransferase VC2332	2.3610108	NS	NS
Signal transduction histidine kinase	2.360962	NS	3.807263
Nitrate reductase cytochrome c550-type subunit	2.3607128	NS	3.031758
Poly(A) polymerase (EC 2.7.7.19)	2.3587563	NS	3.419063
Putative protein-S-isoprenylcysteine methyltransferase	2.3408287	NS	4.956035
5-methylaminomethyl-2-thiouridine-forming enzyme mnmC	2.3400133	NS	NS
Formamidopyrimidine-DNA glycosylase (EC 3.2.2.23)	2.3392522	NS	NS
C4-dicarboxylate transport transcriptional regulatory protein	2.3380098	NS	20.7279
Isochorismatase (EC 3.3.2.1) of siderophore biosynthesis	2.334391	NS	NS
Diaminopimelate epimerase (EC 5.1.1.7)	2.330744	NS	NS
3'-to-5' exoribonuclease RNase R	2.330175	NS	NS
L-serine dehydratase (EC 4.3.1.17)	2.3241944	NS	NS
Putative regulator protein	2.318987	NS	2.577148
RND efflux system, outer membrane lipoprotein CmeC	2.3189244	NS	NS
Protein ThiJ	2.3181689	NS	NS
Fimbrial protein pilin	2.3144357	NS	3.221888
5-nucleotidase SurE (EC 3.1.3.5)	2.3141794	NS	2.383641
Cystathionine gamma-synthase (EC 2.5.1.48)	2.3134406	NS	NS
Amino acid ABC transporter, periplasmic amino acid-binding protein	2.3034685	NS	NS
Superoxide dismutase [Cu-Zn] precursor (EC 1.15.1.1)	2.3003666	NS	NS
tRNA pseudouridine 13 synthase (EC 4.2.1.-)	2.2914948	NS	NS
FIG027190: Putative transmembrane protein	2.2806547	NS	3.263454
tRNA uridine 5-carboxymethylaminomethyl modification enzyme GidA	2.2625494	NS	NS
Chromate transport protein ChrA	2.2618177	2.289316	5.51203
Galactose operon repressor, GalR-LacI family of transcriptional regulators	2.2563038	NS	NS
Dihydrofolate synthase (EC 6.3.2.12) / Folylpolyglutamate synthase (EC 6.3.2.17	2.249957	NS	NS
Nucleoside-diphosphate-sugar epimerase	2.2480888	NS	NS
Flagellar hook-associated protein FlgL	2.2463586	NS	NS
hypothetical protein	2.2372055	NS	NS
GMP reductase (EC 1.7.1.7)	2.2356791	NS	NS
Transcriptional regulator, LysR family	2.2219005	NS	NS
hypothetical protein	2.2199478	NS	2.902826
[Protein-PII] uridylyltransferase (EC 2.7.7.59)	2.2157984	NS	3.433445
General secretion pathway protein D	2.2143822	NS	2.09154

Anaerobic glycerol-3-phosphate dehydrogenase subunit C (EC 1.1.5.3)	2.20668	NS	3.70566
Protein-L-isoaspartate O-methyltransferase (EC 2.1.1.77)	2.2058172	NS	2.854161
Regulator of sigma D	2.205115	NS	NS
hypothetical protein	2.2039194	NS	NS
Transcriptional regulator, LysR family	2.202769	NS	NS
Phosphoglycerol transferase I (EC 2.7.8.20)	2.1933377	NS	NS
Oligopeptidase A (EC 3.4.24.70)	2.1909428	2.947259	NS
Predicted D-lactate dehydrogenase, Fe-S protein, FAD/FMN-containing	2.1898384	NS	NS
Spermidine Putrescine ABC transporter permease component PotB (TC 3.A.1.11.1)	2.1892068	NS	NS
Phosphate:acyl-ACP acyltransferase PlsX	2.1840563	NS	NS
Flagellar sensor histidine kinase FleS	2.1772754	2.060877	3.139379
Flp pilus assembly protein TadD, contains TPR repeat	2.1692927	4.621865	NS
hypothetical protein	2.1670592	NS	NS
nonspecific acid phosphatase precursor	2.1640363	NS	5.42063
Multicopper oxidase	2.1603348	NS	NS
hypothetical protein	2.1598792	NS	NS
PrpF protein involved in 2-methylcitrate cycle	2.158008	NS	11.90218
Transcriptional regulator	2.1551664	NS	NS
Argininosuccinate synthase (EC 6.3.4.5)	2.148654	NS	NS
Uncharacterized iron-regulated protein	2.144782	NS	3.723993
DnaK-related protein	2.1445506	NS	NS
Exported zinc metalloprotease YfgC precursor	2.1303604	NS	NS
hypothetical protein	2.1278176	NS	NS
Lipoprotein releasing system transmembrane protein LolC	2.122141	NS	NS
Hypothetical Transcriptional Regulator	2.1204119	NS	NS
Beta-galactosidase (EC 3.2.1.23) / Beta-glucosidase/6-phospho-beta-glucosidase	2.1192954	NS	8.384006
Sugar binding protein of sugar ABC transporter	2.1086817	NS	NS
Flagellar hook-associated protein FlgK	2.106341	NS	8.986894
Nitrate ABC transporter, ATP-binding protein	2.1012132	NS	NS
Flagellar hook-associated protein FlgK	2.1011608	NS	16.0425
SgrR, sugar-phosphate stress, transcriptional activator of SgrS small RNA	2.1009822	NS	NS
HTH-type transcriptional regulator IlvY	2.097533	NS	NS
Transcriptional regulator, AraC family	2.0939493	NS	4.149908
putative; ORF located using Glimmer/Genemark	2.0933728	NS	NS
ABC-type multidrug transport system, permease component	2.086592	NS	NS
Adenosylcobinamide-phosphate guanylyltransferase (EC 2.7.7.62)	2.085703	NS	NS
Aspartokinase (EC 2.7.2.4) / Homoserine dehydrogenase (EC 1.1.1.3)	2.083541	NS	NS

DNA polymerase III alpha subunit (EC 2.7.7.7)	2.0764935	NS	NS
Lipoprotein NlpD	2.0750525	NS	NS
hypothetical protein	2.0747108	NS	NS
Heat shock protein HtpX / FIG017973: domain of unknown function	2.061469	NS	NS
Pyrrolidone-carboxylate peptidase (EC 3.4.19.3)	2.0559328	NS	NS
RNA polymerase sigma factor RpoD	2.0559328	NS	NS
Tricarboxylate transport protein TctB	2.0559328	NS	NS
hypothetical protein	2.0559328	NS	NS
hypothetical protein	2.0559328	NS	NS
Inosose isomerase (EC 5.3.99.-)	2.0559328	NS	NS
Arginyl-tRNA synthetase (EC 6.1.1.19)	2.0559328	NS	NS
Putative aminotransferase in phosphonate-related cluster	2.0559328	NS	NS
2-aminoethylphosphonate ABC transporter periplasmic binding component (TC 3.A.1	2.0559328	NS	NS
BatA (Bacteroides aerotolerance operon)	2.0559328	NS	NS
hypothetical protein	2.0559328	NS	NS
Nitrate ABC transporter, permease protein	2.0559328	NS	NS
Glycogen synthase, ADP-glucose transglucosylase (EC 2.4.1.21)	2.0559328	NS	NS
Heavy metal sensor histidine kinase	2.0559328	NS	NS
hypothetical protein	2.0559328	NS	NS
L-asparaginase (EC 3.5.1.1)	2.0559328	NS	NS
PTS system, N-acetylgalactosamine-specific IID component (EC 2.7.1.69)	2.0559328	NS	NS
PTS system, N-acetylgalactosamine-specific IIB component (EC 2.7.1.69)	2.0559328	NS	NS
hypothetical protein	2.0559328	NS	NS
Polyribonucleotide nucleotidyltransferase (EC 2.7.7.8)	2.0559328	NS	NS
hypothetical protein	2.0528421	NS	6.094233
Flagellar M-ring protein FliF	2.048578	2.103639	3.589294
L-aspartate oxidase (EC 1.4.3.16)	2.0470474	NS	NS
Flp pilus assembly protein TadB	2.0453174	14.05842	NS
Hydroxymethylglutaryl-CoA reductase (EC 1.1.1.34)	2.0362046	NS	2.478388
Glycerol-3-phosphate acyltransferase (EC 2.3.1.15)	2.0311153	NS	NS
Sugar diacid utilization regulator SdaR	2.0301952	NS	NS
Flavohemoprotein (Hemoglobin-like protein) (Flavohemoglobin) (Nitric oxide diox	2.027945	4.855946	NS
Signal transduction histidine kinase	2.027697	NS	NS
TonB-dependent receptor	2.0255797	NS	4.298257
Glycerophosphoryl diester phosphodiesterase (EC 3.1.4.46)	2.0185304	NS	3.103301
Beta-N-acetylhexosaminidase, (GlcNAc)2 catabolism	2.0184424	2.018442	6.644849
DNA-binding response regulator, LuxR family	2.0179563	NS	2.195736

Permease of the drug/metabolite transporter (DMT) superfamily	2.0111935	2.071576	4.138587
Transcriptional regulator, AraC family	2.0083818	NS	NS
DNA polymerase III subunits gamma and tau (EC 2.7.7.7)	2.0059292	NS	2.046717
Ferric siderophore transport system, periplasmic binding protein TonB	2.005544	2.296907	NS
Anhydro-N-acetylmuramic acid kinase (EC 2.7.1.-)	2.001347	NS	NS
Chromosomal replication initiator protein DnaA	2.000126	NS	NS
Acetyltransferase	-2.0052705	-3.32852	NS
hypothetical protein	-2.0094075	NS	NS
Preprotein translocase subunit SecG (TC 3.A.5.1.1)	-2.017569	NS	NS
Tellurite resistance protein	-2.0195274	NS	NS
hypothetical protein	-2.026549	NS	NS
hypothetical protein	-2.0496402	NS	NS
ABC-type multidrug transport system, ATPase and permease component	-2.0513568	-3.36617	NS
3-dehydroquinate dehydratase II (EC 4.2.1.10)	-2.057474	-2.58948	-2.28458
Protein yciN	-2.0632617	NS	NS
LSU ribosomal protein L27p	-2.0659502	-3.09346	-3.07308
hypothetical protein	-2.0692267	NS	NS
Pyruvate/2-oxoglutarate dehydrogenase complex, dihydrolipoamide dehydrogenase c	-2.0782237	-2.52644	NS
Stringent starvation protein B	-2.085506	-2.05464	NS
hypothetical protein	-2.0857985	-3.0607	NS
Permease of the drug/metabolite transporter (DMT) superfamily	-2.0898557	NS	NS
Molybdenum cofactor biosynthesis protein MoaA	-2.0973907	NS	NS
tRNA-guanine transglycosylase (EC 2.4.2.29)	-2.117573	-3.72911	-3.1117
ATPase involved in DNA repair	-2.1224654	-2.09894	-2.40098
Ribosomal protein L11 methyltransferase (EC 2.1.1.-)	-2.124322	-2.31784	NS
Nucleoid-associated protein NdpA	-2.149329	-2.69757	NS
Uncharacterized conserved protein	-2.1793926	-2.23378	NS
Heat shock protein HslJ	-2.1965508	NS	-2.69114
Transcription antitermination protein NusG	-2.2221994	NS	NS
SSU ribosomal protein S20p	-2.2322054	-2.14117	NS
Predicted amidohydrolase	-2.2343657	-2.42141	NS
Stringent starvation protein A	-2.2441401	NS	NS
Protein-export membrane protein SecD (TC 3.A.5.1.1)	-2.2527306	-4.4982	NS
Alkylphosphonate utilization operon protein PhnA	-2.2548108	-2.72226	-3.38181
hypothetical protein	-2.2837787	NS	NS
Thioredoxin	-2.2903166	-3.33959	NS
SH3 domain protein	-2.3036113	-3.62976	NS
Acetyltransferase	-2.304022	-2.38195	NS
hypothetical protein	-2.3342729	-3.365	NS

Probable type IV pilus assembly FimV-related transmembrane protein	-2.356212	-2.39213	NS
hypothetical protein	-2.358299	-3.24584	NS
LSU m5C1962 methyltransferase RlmI	-2.362093	-2.13382	NS
hypothetical protein	-2.370647	-3.19323	NS
Transcription termination protein NusB	-2.3865533	-2.81601	NS
Aerobic respiration control protein arcA	-2.3901467	-2.62871	NS
Adenosine deaminase (EC 3.5.4.4)	-2.4058738	-3.34014	NS
hypothetical protein	-2.409579	-2.0124	NS
Lipoprotein nlpI precursor	-2.4228418	-3.01951	-2.94158
Quinolinate phosphoribosyltransferase [decarboxylating] (EC 2.4.2.19)	-2.4616697	-3.62549	-2.60784
Vitamin B12 ABC transporter, permease component BtuC	-2.4709415	-2.96838	NS
ATP-dependent RNA helicase DbpA	-2.4717946	-4.08918	NS
DNA-binding protein Fis	-2.4758687	NS	NS
Ribose ABC transport system, high affinity permease RbsD (TC 3.A.1.2.1)	-2.4826012	-3.76359	NS
Mutator mutT protein (7,8-dihydro-8-oxoguanine-triphosphatase) (EC 3.6.1.-)	-2.4961164	NS	NS
ATP-dependent RNA helicase VC1407	-2.4971998	-3.48567	NS
COG0398: uncharacterized membrane protein	-2.5381055	NS	NS
Ribosomal RNA large subunit methyltransferase N (EC 2.1.1.-)	-2.5645993	NS	-2.88891
Preprotein translocase subunit SecE (TC 3.A.5.1.1)	-2.5979316	-2.98	-2.99255
putative transport system permease protein	-2.6136	-2.59685	NS
Fructose-1,6-bisphosphatase, GlpX type (EC 3.1.3.11)	-2.6344335	NS	-2.50383
Purine nucleotide synthesis repressor	-2.6531482	-4.5048	-4.31488
Short chain fatty acids transporter	-2.6542306	-2.35786	NS
Probable GTPase related to EngC	-2.7446728	-2.17269	NS
Ribonuclease E inhibitor RraB	-2.7580595	NS	-2.76579
Manganese-dependent inorganic pyrophosphatase (EC 3.6.1.1)	-2.7732368	NS	NS
Putative protease	-2.7978938	-2.10744	NS
Histone acetyltransferase HPA2	-2.7994504	-2.27837	NS
Arylsulfatase (EC 3.1.6.1)	-2.8214326	NS	NS
Preprotein translocase subunit YajC (TC 3.A.5.1.1)	-2.84544	-2.14992	-3.6928
hypothetical protein	-2.852739	-3.45584	NS
Ribonucleotide reductase of class III (anaerobic), large subunit (EC 1.17.4.2)	-2.896299	-2.92646	-2.85622
Maltose/maltodextrin ABC transporter, permease protein MalG	-2.9061017	-3.09875	NS
hypothetical protein	-2.9737103	-3.53385	NS
YaeQ protein	-2.9847617	-4.00146	NS
Regulator of nucleoside diphosphate kinase	-3.0835826	-5.6367	NS
YrdC/Sua5 family protein, required for threonylcarbamoyladenosine (t(6)A) forma	-3.093876	-3.55459	NS

Ribose-phosphate pyrophosphokinase (EC 2.7.6.1)	-3.1140018	-2.61221	NS
Uncharacterized protein conserved in bacteria	-3.121173	-7.96287	NS
hypothetical protein	-3.142826	-3.53359	-2.35204
Aspartokinase (EC 2.7.2.4)	-3.2229664	-3.13521	-3.50249
Putative membrane protein	-3.3580072	-2.50256	NS
Adenylate kinase (EC 2.7.4.3)	-3.9976776	-2.67107	-2.68292
Permease of the major facilitator superfamily	-4.0015993	-3.05551	-3.77856
hypothetical protein	-4.062897	-4.05962	NS
Queuosine biosynthesis QueD, PTPS-I	-4.0924773	-4.84107	-5.79169
Uncharacterized protein conserved in bacteria	-4.323332	-5.62742	-5.25184
SSU ribosomal protein S21p	-4.3781548	-2.91034	NS
Regulator of competence-specific genes	-4.5268135	-3.15569	NS
Guanylate kinase (EC 2.7.4.8)	-5.6962714	-4.90619	-3.62912
Unknown, probable transcriptional regulator	NS	46.1203	NS
RNA polymerase sigma-54 factor RpoN	NS	27.12909	3.808779
Periplasmic nitrate reductase component NapE	NS	22.49176	2.164153
Chitinase (EC 3.2.1.14)	NS	22.29679	NS
FIG026291: Hypothetical periplasmic protein	NS	21.45245	3.916338
Transcriptional regulator, ArsR family	NS	19.8818	NS
Transcriptional regulator, AraC family	NS	18.55934	NS
hypothetical protein	NS	16.3581	NS
Transposase and inactivated derivatives	NS	15.95393	4.203063
Transcriptional regulator	NS	15.45806	NS
hypothetical protein	NS	15.44581	NS
hypothetical protein	NS	15.42027	NS
FIG002577: Putative lipoprotein precursor	NS	14.70399	NS
pR99_vep40	NS	14.21037	NS
Flp pilus assembly protein	NS	14.01807	NS
Uncharacterized protein, similar to the N-terminal domain of Lon protease	NS	12.9131	3.010788
hypothetical protein	NS	12.82822	NS
Methyl-accepting chemotaxis protein	NS	12.77338	4.458769
NAD-dependent glyceraldehyde-3-phosphate dehydrogenase (EC 1.2.1.12)	NS	12.45717	2.663526
Glutaredoxin 3	NS	12.41621	NS
hypothetical protein	NS	12.2201	NS
Putative inner membrane protein	NS	11.66353	NS
Flp pilus assembly protein	NS	11.48201	NS
Methionyl-tRNA formyltransferase (EC 2.1.2.9)	NS	11.44326	7.467871
YoeB toxin protein	NS	11.4186	NS
hypothetical protein	NS	11.21453	2.433077
Multidrug resistance protein A	NS	10.62447	NS
Phosphoserine phosphatase	NS	10.55191	9.790239
hypothetical protein	NS	10.4917	NS
Fic family protein	NS	9.885819	NS

hypothetical protein	NS	9.875895	NS
Putative membrane protein	NS	9.695326	NS
Predicted transcriptional regulator	NS	9.673851	NS
DNA-binding response regulator	NS	9.542086	2.73449
Sigma factor RpoE negative regulatory protein RseA	NS	9.452895	NS
pR99_vep31	NS	9.377135	6.54623
Uxu operon transcriptional regulator	NS	9.060198	NS
Transposase and inactivated derivatives	NS	8.948555	4.86725
hypothetical protein	NS	8.790865	NS
Flagellar biosynthesis protein FlgN	NS	8.746007	NS
probable exported protein YPO3233	NS	8.683262	NS
surface localized decaheme cytochrome c lipoprotein, MtrC	NS	8.661251	NS
ABC-type uncharacterized transport system, permease component	NS	8.607057	7.271247
Transcriptional regulator, IclR family	NS	8.533197	NS
Ascorbate utilization transcriptional regulator UlaR, HTH-type	NS	8.524795	NS
S-adenosylmethionine synthetase (EC 2.5.1.6)	NS	8.514127	2.798301
Negative regulator of flagellin synthesis FlgM	NS	8.503853	NS
hypothetical protein	NS	8.424397	2.992375
Protein F-related protein	NS	8.413033	NS
RNA polymerase sigma-70 factor, ECF subfamily	NS	8.373605	NS
Pyruvate formate-lyase activating enzyme (EC 1.97.1.4)	NS	8.366781	NS
Succinate-semialdehyde dehydrogenase [NADP+] (EC 1.2.1.16)	NS	8.355952	NS
Low molecular weight protein tyrosine phosphatase (EC 3.1.3.48)	NS	8.193376	NS
Nitrite-sensitive transcriptional repressor NsrR	NS	8.178842	NS
hypothetical protein	NS	8.010679	NS
hypothetical protein	NS	7.990999	NS
Transcriptional regulator, TetR family	NS	7.895486	NS
Putative inner membrane protein YjeT (clustered with HflC)	NS	7.886826	NS
Large exoproteins involved in heme utilization or adhesion	NS	7.884373	NS
FIG106692: Outer membrane lipoprotein	NS	7.659782	3.002952
Thymidylate kinase	NS	7.548713	NS
Flagellar motor rotation protein MotA	NS	7.542477	3.590035
Topoisomerase IV subunit B (EC 5.99.1.-)	NS	7.503558	NS
hypothetical protein	NS	7.440616	2.198054
DNA polymerase III psi subunit (EC 2.7.7.7)	NS	7.409012	2.634232
pR99_vep56	NS	7.400682	4.498708
Predicted DNA-binding protein	NS	7.225947	NS
Permease of the major facilitator superfamily	NS	7.210206	4.437656
RND efflux system, inner membrane transporter	NS	7.185519	NS

CmeB			
DNA polymerase III epsilon subunit (EC 2.7.7.7)	NS	7.154403	10.50728
Chemotactic transducer-related protein	NS	7.127604	3.192313
ISBma1, transposase	NS	7.050584	NS
Metallo-beta-lactamase family protein, RNA-specific	NS	6.972295	4.199346
FIG111991: hypothetical protein	NS	6.900707	NS
Acriflavin resistance protein	NS	6.871643	NS
O-acetylhomoserine sulfhydrylase (EC 2.5.1.49)	NS	6.810109	NS
Cytochrome c-type protein NapC	NS	6.695212	3.277108
SN-glycerol-3-phosphate transport system permease protein UgpA (TC 3.A.1.1.3)	NS	6.677998	NS
Transcriptional regulator	NS	6.664003	2.633363
Nucleoside permease NupC	NS	6.641904	NS
Arylsulfatase (EC 3.1.6.1)	NS	6.615501	NS
Predicted exporter of the RND superfamily	NS	6.580833	4.562882
Zn-dependent hydrolase (EC 3.-.-.-)	NS	6.563469	2.907794
hypothetical protein	NS	6.538224	2.457313
Predicted hydrolase of the metallo-beta-lactamase superfamily, clustered with K	NS	6.528672	7.862558
Response regulator	NS	6.438239	4.141798
Sigma factor RpoE negative regulatory protein RseB precursor	NS	6.423748	NS
Transcriptional regulator	NS	6.407932	NS
7,8-didemethyl-8-hydroxy-5-deazariboflavin synthase subunit 2	NS	6.40727	11.17647
Toxin secretion ATP-binding protein	NS	6.37802	NS
Permease of the major facilitator superfamily	NS	6.350598	6.413693
Iron-sulfur cluster-binding protein	NS	6.33068	2.804255
Probable L-ascorbate-6-phosphate lactonase UlaG (EC 3.1.1.-) (L-ascorbate utili	NS	6.323865	NS
hypothetical protein	NS	6.315115	5.272431
hypothetical protein	NS	6.291917	4.263629
hypothetical protein	NS	6.273459	NS
Putative HTH-type transcriptional regulator ybaO	NS	6.261986	5.148615
Outer membrane lipoprotein-sorting protein	NS	6.234162	NS
Sigma factor RpoE regulatory protein RseC	NS	6.22063	NS
ISBma1, transposase	NS	6.137998	3.084461
TRAP-type C4-dicarboxylate transport system, large permease component	NS	6.117555	7.185109
hypothetical protein	NS	6.111663	8.381614
hypothetical protein	NS	6.097192	NS
Phosphoglycerate transport system transcriptional regulatory protein PgtA	NS	6.082424	9.528832
Rossmann fold nucleotide-binding protein Smf possibly involved in DNA uptake	NS	6.059255	NS
Extracellular solute-binding protein, family 3/GGDEF domain protein	NS	6.050036	3.53109

hypothetical protein	NS	6.038546	NS
HD-domain protein	NS	6.034125	3.509209
Nitrite transporter from formate/nitrite family	NS	5.993912	NS
Tyrosyl-tRNA synthetase (EC 6.1.1.1) ## cluster 1	NS	5.870317	NS
Glutathione S-transferase (EC 2.5.1.18)	NS	5.858734	NS
Fusaric acid resistance protein fusE	NS	5.83528	9.530993
Biotin synthase (EC 2.8.1.6)	NS	5.833773	NS
pR99_vep13	NS	5.808457	NS
O-succinylbenzoate-CoA synthase (EC 4.2.1.-)	NS	5.792969	2.445356
Flagellar synthesis regulator FleN	NS	5.791265	2.119378
Uridine phosphorylase (EC 2.4.2.3)	NS	5.779126	NS
hypothetical protein	NS	5.777963	NS
ABC-type sugar transport system, ATPase component	NS	5.770969	7.073522
Permease of the major facilitator superfamily	NS	5.754135	2.920057
outer membrane protein, MtrB	NS	5.752618	NS
Dihydroneopterin triphosphate pyrophosphohydolase type 2	NS	5.735094	3.143793
pR99_vep58	NS	5.734087	3.628934
ClpB protein	NS	5.732785	NS
Membrane-fusion protein	NS	5.694051	3.055714
Extracellular deoxyribonuclease Dns (EC 3.1.21.-)	NS	5.667341	NS
hypothetical protein	NS	5.639995	NS
hypothetical protein	NS	5.632246	NS
Multidrug resistance protein 2	NS	5.623928	2.419998
membrane protein	NS	5.620293	NS
hypothetical protein	NS	5.596438	3.930999
Transcriptional regulator, AraC family	NS	5.58985	NS
Murein endopeptidase	NS	5.542912	NS
Branched-chain amino acid transport ATP-binding protein LivF (TC 3.A.1.4.1)	NS	5.541038	NS
Aspartate/tyrosine/aromatic aminotransferase	NS	5.54082	8.530037
pR99_vep04	NS	5.502737	4.225995
Capsular polysaccharide synthesis enzyme CpsB	NS	5.485167	NS
FIG005666: putative helicase	NS	5.467385	NS
ABC-type amino acid transport/signal transduction system	NS	5.454673	NS
hypothetical protein	NS	5.435696	NS
Ribose/xylose/arabinose/galactoside ABC-type transport systems, permease compon	NS	5.414871	NS
RNA polymerase sigma factor for flagellar operon	NS	5.40807	NS
hypothetical protein	NS	5.374476	8.927035
GGDEF family protein	NS	5.373334	4.369651
D-alanyl-D-alanine carboxypeptidase (EC 3.4.16.4)	NS	5.371105	NS
Apolipoprotein N-acyltransferase (EC 2.3.1.-) / Copper homeostasis protein CutE	NS	5.354291	NS
Lipoprotein releasing system transmembrane	NS	5.292759	NS

protein LolE			
Ribose ABC transport system, ATP-binding protein RbsA (TC 3.A.1.2.1)	NS	5.278161	NS
pR99_vep59	NS	5.272421	3.024249
hypothetical protein	NS	5.269057	NS
Putative ATP-dependent Lon protease	NS	5.240175	NS
Transcriptional regulator, MarR family	NS	5.239327	NS
hypothetical protein	NS	5.238221	NS
GGDEF domain protein	NS	5.200305	6.166299
Biotin-protein ligase (EC 6.3.4.15) / Biotin operon repressor	NS	5.166174	2.39464
Membrane-associated zinc metalloprotease	NS	5.157984	NS
FIG001592: Phosphocarrier protein kinase/phosphorylase, nitrogen regulation ass	NS	5.154534	NS
Anti anti-sigma regulatory factor SypA	NS	5.140286	12.17396
Potential queD like 2	NS	5.03772	NS
hypothetical protein	NS	5.000691	NS
Serine/threonine protein kinase PrkC, regulator of stationary phase	NS	4.961401	5.920676
Acyl-phosphate:glycerol-3-phosphate O-acyltransferase PlsY	NS	4.943143	3.307802
CcdA protein (antitoxin to CcdB)	NS	4.912883	3.826168
Hypothetical protein in cluster with HutR, VCA0066 homolog	NS	4.91025	6.056172
Arylsulfatase (EC 3.1.6.1)	NS	4.877459	NS
hypothetical protein	NS	4.857378	NS
2,3-dihydroxybenzoate-AMP ligase (EC 2.7.7.58)	NS	4.84734	NS
Formate efflux transporter (TC 2.A.44 family)	NS	4.798073	NS
hypothetical protein	NS	4.769379	NS
Permease of the drug/metabolite transporter (DMT) superfamily	NS	4.753079	NS
Succinate dehydrogenase iron-sulfur protein (EC 1.3.99.1)	NS	4.741779	NS
hypothetical protein	NS	4.741276	NS
Nitrite reductase [NAD(P)H] small subunit (EC 1.7.1.4)	NS	4.734997	NS
Metallo-beta-lactamase superfamily protein PA0057	NS	4.728792	6.265328
hypothetical protein	NS	4.689222	12.64549
Bacterial surface protein	NS	4.679295	NS
Anti-anti-sigma regulatory factor	NS	4.665831	4.891569
hypothetical protein	NS	4.607416	NS
pR99_vep60	NS	4.601345	NS
hypothetical protein	NS	4.589597	2.992132
pR99_mazF///pR99_mazE	NS	4.584055	NS
hypothetical protein	NS	4.574401	NS
Membrane protein	NS	4.57386	NS
putative	NS	4.567668	3.529148

TRAP-type transport system, small permease component, predicted N-acetylneurami	NS	4.564681	11.4504
Zinc ABC transporter, periplasmic-binding protein ZnuA	NS	4.557753	NS
Protein of unknown function DUF81	NS	4.543744	2.426607
Glutathione S-transferase (EC 2.5.1.18)	NS	4.521714	NS
Membrane-associated phospholipid phosphatase	NS	4.51697	2.219338
Oligo-1,6-glucosidase (EC 3.2.1.10)	NS	4.49787	NS
Chromosome segregation ATPase	NS	4.454456	6.938818
4-hydroxy-3-methylbut-2-enyl diphosphate reductase (EC 1.17.1.2)	NS	4.44357	NS
probable extracellular solute-binding protein	NS	4.365556	NS
hypothetical protein	NS	4.347176	NS
Transcriptional regulator, GntR family	NS	4.34472	NS
Maltose regulon regulatory protein MalI (repressor for malXY)	NS	4.342106	NS
Capsular polysaccharide synthesis enzyme CpsC, polysaccharide export	NS	4.339153	NS
H(+)/Cl(-) exchange transporter ClcA	NS	4.328774	NS
Cell division protein FtsJ / Ribosomal RNA large subunit methyltransferase E (E	NS	4.3241	NS
Phosphoserine phosphatase (EC 3.1.3.3)	NS	4.320269	3.263265
hypothetical protein	NS	4.303489	4.046626
Lactoylglutathione lyase	NS	4.298824	3.62264
ATP-dependent DNA helicase RecG (EC 3.6.1.-)	NS	4.297174	5.792419
Non-ribosomal peptide synthetase modules, siderophore biosynthesis	NS	4.284414	NS
Ribosome-associated heat shock protein implicated in the recycling of the 50S s	NS	4.259928	NS
pR99_vep12	NS	4.257535	NS
Transcriptional regulator, LysR family, in formaldehyde detoxification operon	NS	4.253669	NS
Lipase-related protein	NS	4.248165	NS
TPR repeat protein	NS	4.245316	NS
Cobalamin synthase	NS	4.24245	NS
hypothetical protein	NS	4.233888	NS
Phosphoglycerol transferase	NS	4.205035	NS
ISBma1, transposase	NS	4.203361	2.903872
Transposase and inactivated derivatives	NS	4.195397	3.365765
DNA polymerase III beta subunit (EC 2.7.7.7)	NS	4.186789	NS
GGDEF family protein	NS	4.167585	NS
hypothetical protein	NS	4.165513	NS
Glutamate synthase [NADPH] large chain (EC 1.4.1.13)	NS	4.153332	NS
hypothetical protein	NS	4.143488	16.22814
1-deoxy-D-xylulose 5-phosphate synthase (EC 2.2.1.7)	NS	4.139219	NS
Transcriptional regulator, VCA0231 ortholog	NS	4.134259	NS

DNA-binding heavy metal response regulator	NS	4.134098	NS
Nucleoside permease NupC	NS	4.126966	8.092066
Flagellar basal-body P-ring formation protein FlgA	NS	4.091673	3.240857
Acetyltransferase	NS	4.078538	2.731777
Response regulator of citrate/malate metabolism	NS	4.06145	NS
Regulatory protein RecX	NS	4.05475	2.621633
D-lactate dehydrogenase (EC 1.1.1.28)	NS	4.007041	2.689382
Accessory colonization factor AcfD precursor	NS	4.003623	NS
Lumazine protein, riboflavin synthase homolog	NS	3.999564	NS
Glycerol-3-phosphate regulon repressor, DeoR family	NS	3.998983	NS
hypothetical protein	NS	3.994679	15.89464
hypothetical protein	NS	3.967116	5.434009
DNA-3-methyladenine glycosylase (EC 3.2.2.20)	NS	3.961325	NS
Protein of avirulence locus ImpE	NS	3.953795	NS
HIPA PROTEIN	NS	3.951761	NS
Error-prone repair protein UmuD	NS	3.948993	2.089362
Potassium channel protein	NS	3.937569	15.76332
hypothetical protein	NS	3.936728	NS
hypothetical protein	NS	3.931977	9.030752
hypothetical protein	NS	3.92318	NS
hypothetical protein	NS	3.917819	NS
Conserved membrane protein	NS	3.889065	2.265244
membrane protein	NS	3.882069	NS
putative lipoprotein L	NS	3.861702	NS
Methyl-accepting chemotaxis protein	NS	3.84652	NS
FIG067310: hypothetical protein	NS	3.817754	6.574144
Phosphate ABC transporter, periplasmic phosphate-binding protein PstS (TC 3.A.1	NS	3.808095	NS
S-adenosyl-L-methionine dependent methyltransferase, similar to cyclopropane-fa	NS	3.777833	4.127845
hypothetical protein	NS	3.755144	NS
Helix-turn-helix protein, CopG family	NS	3.752151	NS
Gluconokinase (EC 2.7.1.12)	NS	3.747518	NS
Transposase and inactivated derivatives	NS	3.74723	2.92202
D-alanyl-D-alanine carboxypeptidase (EC 3.4.16.4)	NS	3.746297	2.530957
Outer membrane protein N, non-specific porin	NS	3.741135	NS
Adenylate cyclase (EC 4.6.1.1)	NS	3.732303	NS
Putative cytoplasmic protein	NS	3.730185	3.362075
Transcriptional regulator VpsT	NS	3.720902	NS
hypothetical protein	NS	3.70898	NS
RTX toxins and related Ca2+-binding proteins	NS	3.705424	NS
Flagellum-specific ATP synthase FliI	NS	3.704629	NS
FOG: GGDEF domain	NS	3.686244	NS
Transcriptional regulator, AraC family	NS	3.68069	2.673129
Hypothetical protein DUF454	NS	3.665523	NS

Evolved beta-D-galactosidase, alpha subunit	NS	3.664319	9.188948
Sugar transferase SypR involved in lipopolysaccharide synthesis	NS	3.661115	15.82595
Cold shock protein CspD	NS	3.658621	NS
Pantothenate kinase type III, CoaX-like (EC 2.7.1.33)	NS	3.658299	NS
hypothetical protein	NS	3.658259	NS
Molybdenum ABC transporter, periplasmic molybdenum-binding protein ModA (TC 3.A	NS	3.654606	2.764654
Hypothetical nudix hydrolase YeaB	NS	3.649558	NS
Hydrogen peroxide-inducible genes activator	NS	3.64479	NS
Phosphate transport ATP-binding protein PstB (TC 3.A.1.7.1)	NS	3.633321	NS
GNAT family acetyltransferase YiiD potentially involved in tRNA processing	NS	3.614939	NS
4-amino-6-deoxy-N-Acetyl-D-hexosaminyl-(Lipid carrier) acetyltrasferase	NS	3.614757	NS
Kynurenine 3-monooxygenase (EC 1.14.13.9)	NS	3.601193	5.466429
transposase and inactivated derivative	NS	3.600711	2.470476
Succinyl-CoA synthetase, alpha subunit	NS	3.598096	NS
hypothetical protein	NS	3.582917	4.652159
Uncharacterized protein YtfM precursor	NS	3.582029	6.174675
FIG065221: ATPase, AAA family	NS	3.580472	NS
Flagellin protein FlaF	NS	3.578903	3.968288
L-proline glycine betaine ABC transport system permease protein ProW (TC 3.A.1.	NS	3.574404	NS
Regulatory protein LuxO	NS	3.570586	NS
hypothetical protein	NS	3.568344	NS
Functional role page for Chaperone protein TorD	NS	3.556776	NS
Zinc-regulated TonB-dependent outer membrane receptor	NS	3.554333	NS
transposase and inactivated derivative	NS	3.547935	2.256754
Rrf2-linked NADH-flavin reductase	NS	3.546248	NS
Sodium-Choline Symporter	NS	3.537825	NS
hypothetical protein	NS	3.516486	4.929526
hypothetical protein	NS	3.510224	NS
MSHA pilin protein MshA BUT NOT	NS	3.505442	NS
Zinc ABC transporter, inner membrane permease protein ZnuB	NS	3.502504	NS
5-Enolpyruvylshikimate-3-phosphate synthase (EC 2.5.1.19)	NS	3.498247	2.366224
Lysophospholipase L2 (EC 3.1.1.5)	NS	3.49735	NS
Manganese superoxide dismutase (EC 1.15.1.1)	NS	3.488762	10.96764
transposase and inactivated derivative	NS	3.486342	2.975026
Nitrite reductase [NAD(P)H] small subunit (EC 1.7.1.4)	NS	3.483955	8.474683
Glycine cleavage system transcriptional activator GcvA	NS	3.472024	NS
FIG002208: Acetyltransferase (EC 2.3.1.-)	NS	3.470565	NS

hypothetical protein	NS	3.469223	3.92987
Outer membrane protein assembly factor YaeT precursor	NS	3.466239	NS
Membrane fusion protein of RND family multidrug efflux pump	NS	3.462693	NS
hypothetical protein	NS	3.457785	NS
Hypotehtical protein in Cytochrome oxidase biogenesis cluster	NS	3.438026	NS
SeqA protein, negative modulator of initiation of replication	NS	3.437429	NS
Putative MCP-type signal transduction protein	NS	3.424255	2.602193
hypothetical protein	NS	3.422518	2.443609
hypothetical protein	NS	3.413247	3.904439
hypothetical protein	NS	3.402497	NS
hypothetical protein	NS	3.401822	NS
hypothetical protein	NS	3.39746	NS
Translation initiation factor SUI1-related protein	NS	3.394143	NS
Ferric siderophore transport system, periplasmic binding protein TonB	NS	3.379466	NS
Functional role page for Anaerobic nitric oxide reductase transcription regulat	NS	3.378167	NS
Cytoplasmic copper homeostasis protein cutC	NS	3.374184	4.236153
transposase and inactivated derivative	NS	3.370625	2.188619
transposase and inactivated derivative	NS	3.362699	2.306015
pR99_vep19	NS	3.361309	4.549897
pR99_vep61	NS	3.361124	NS
Selenoprotein W-related protein	NS	3.360696	NS
hypothetical protein	NS	3.357955	NS
hypothetical protein	NS	3.353674	NS
hypothetical protein	NS	3.347814	6.988169
hypothetical protein	NS	3.346702	NS
Ribonuclease D (EC 3.1.26.3)	NS	3.344844	NS
putative regulatory protein	NS	3.329251	6.096464
Capsular polysaccharide synthesis enzyme CpsA, sugar transferase	NS	3.319366	NS
Transcriptional regulator, AsnC family	NS	3.318915	NS
tRNA nucleotidyltransferase (EC 2.7.7.21) (EC 2.7.7.25)	NS	3.314531	3.521101
GGDEF family protein	NS	3.310958	3.067131
Putative deoxyribonuclease YjjV	NS	3.305346	NS
Mannose-6-phosphate isomerase (EC 5.3.1.8)	NS	3.300856	9.319922
Multidrug resistance protein B	NS	3.300019	NS
N-acetylglucosamine-1-phosphate uridyltransferase (EC 2.7.7.23) / Glucosamine-1	NS	3.295207	NS
Methyltransferase (EC 2.1.1.-)	NS	3.293832	NS
hypothetical protein	NS	3.28967	NS
hypothetical protein	NS	3.28809	NS
Serine/threonine protein kinase (EC 2.7.11.1)	NS	3.283099	NS

hypothetical protein	NS	3.274903	NS
Chemotaxis protein CheD	NS	3.268766	NS
Type II/IV secretion system ATPase TadZ/CpaE, associated with Flp pilus assembl	NS	3.268379	9.989751
6-phosphogluconolactonase (EC 3.1.1.31), eukaryotic type	NS	3.268109	NS
hypothetical protein	NS	3.262322	2.392241
Methylcrotonyl-CoA carboxylase carboxyl transferase subunit (EC 6.4.1.4)	NS	3.258864	6.895303
radical activating enzyme	NS	3.258566	NS
Fucose permease	NS	3.257631	2.872857
TolA protein	NS	3.25648	2.2827
hypothetical protein	NS	3.256449	7.844153
ClpB protein	NS	3.242584	NS
Excinuclease ABC subunit C	NS	3.235654	3.276774
Glutaredoxin	NS	3.232857	NS
Secreted trypsin-like serine protease	NS	3.223477	4.399869
Glycosidase	NS	3.222583	4.384706
trypsin, putative	NS	3.219751	3.493779
Cysteine desulfurase CsdA-CsdE, sulfur acceptor protein CsdE	NS	3.219115	NS
Excinuclease ABC, C subunit-like	NS	3.218488	4.532754
MSHA biogenesis protein MshO	NS	3.214865	4.155046
transposase and inactivated derivative	NS	3.213308	2.673759
proteinase inhibitor, putative	NS	3.213071	NS
hypothetical protein	NS	3.203958	4.60478
Type II/IV secretion system protein TadC, associated with Flp pilus assembly	NS	3.198479	NS
hypothetical protein	NS	3.182384	NS
Aspartate aminotransferase (EC 2.6.1.1)	NS	3.173406	NS
Chemotaxis regulator - transmits chemoreceptor signals to flagelllar motor comp	NS	3.170324	5.78723
transposase and inactivated derivative	NS	3.170111	2.632344
membrane protein	NS	3.169024	NS
hypothetical protein	NS	3.167571	NS
hypothetical protein	NS	3.167493	NS
hypothetical protein	NS	3.160866	NS
Para-aminobenzoate synthase, aminase component (EC 2.6.1.85) / Aminodeoxychoris	NS	3.157461	NS
Tryptophanase (EC 4.1.99.1)	NS	3.154298	7.80204
Magnesium and cobalt efflux protein CorC	NS	3.149694	NS
transposase and inactivated derivative	NS	3.146292	2.841062
DNA primase (EC 2.7.7.-)	NS	3.132607	2.368873
FOG: WD40 repeat	NS	3.126863	NS
hypothetical protein	NS	3.12492	NS
TrkA, Potassium channel-family protein	NS	3.124564	2.413612
hypothetical protein	NS	3.124553	NS

Transcriptional regulator, AraC family	NS	3.122178	3.165384
conserved protein of unknown function; putative YcgN protein	NS	3.118569	13.57201
hypothetical protein	NS	3.117575	NS
hypothetical protein	NS	3.099508	NS
Ribosome small subunit-stimulated GTPase EngC	NS	3.097737	NS
hypothetical protein	NS	3.087756	NS
hypothetical protein	NS	3.083501	NS
Leucyl/phenylalanyl-tRNA--protein transferase (EC 2.3.2.6)	NS	3.080562	NS
HipA protein	NS	3.080172	NS
Cytochrome oxidase biogenesis protein Surf1, facilitates heme A insertion	NS	3.079344	NS
Methyl-accepting chemotaxis protein	NS	3.074728	2.004457
Outer membrane protein romA	NS	3.073468	NS
Transcriptional regulator, SorC family	NS	3.07211	NS
Beta-hexosaminidase (EC 3.2.1.52)	NS	3.061565	NS
hypothetical protein	NS	3.059363	NS
Propionate--CoA ligase (EC 6.2.1.17)	NS	3.055228	NS
Methyl-accepting chemotaxis protein	NS	3.050733	6.116395
Sulfate permease	NS	3.035797	NS
hypothetical protein	NS	3.03419	NS
hypothetical protein	NS	3.032365	NS
hypothetical protein	NS	3.030241	4.157454
Oligopeptide transport system permease protein OppC (TC 3.A.1.5.1)	NS	3.022979	2.823528
conserved hypothetical protein	NS	3.013603	NS
Tyrosine-specific transport protein	NS	3.005713	NS
Phosphate transport system permease protein PstA (TC 3.A.1.7.1)	NS	3.002231	6.611865
Type IV pilus biogenesis protein PilE	NS	3.000374	2.978651
Probable 3-phenylpropionic acid transporter	NS	2.995905	NS
Putative metal chaperone, involved in Zn homeostasis, GTPase of COG0523 family	NS	2.995594	NS
Chaperone protein HtpG	NS	2.993339	NS
Transcriptional regulator, TetR family	NS	2.993121	NS
Two component response regulator	NS	2.98968	10.51531
hypothetical protein	NS	2.989371	4.781708
UDP-glucose dehydrogenase (EC 1.1.1.22)	NS	2.984059	NS
Phosphatidylglycerophosphatase A (EC 3.1.3.27)	NS	2.979487	NS
hypothetical protein	NS	2.975682	NS
Permease of the drug/metabolite transporter (DMT) superfamily	NS	2.973911	NS
Transcriptional regulator, MarR family	NS	2.962291	6.561351
Permease of the drug/metabolite transporter (DMT) superfamily	NS	2.962241	2.310947
ATP-dependent DNA ligase	NS	2.961268	2.348503

Thiamin ABC transporter, transmembrane component	NS	2.960226	NS
PutR, transcriptional activator of PutA and PutP	NS	2.958648	NS
Signal transduction histidine kinase	NS	2.958409	NS
Transcriptional regulator, LysR family	NS	2.953663	NS
hypothetical protein	NS	2.950119	2.870998
hypothetical protein	NS	2.943224	NS
2-oxoglutarate dehydrogenase complex, dehydrogenase component	NS	2.935926	NS
Oxygen-insensitive NAD(P)H nitroreductase (EC 1.-.-.-) / Dihydropteridine reduc	NS	2.935365	NS
Secreted trypsin-like serine protease	NS	2.930874	13.54437
N-acetylmuramoyl-L-alanine amidase (EC 3.5.1.28) AmpD	NS	2.929918	NS
Glycosyltransferase involved in cell wall biogenesis (EC 2.4.-.-)	NS	2.928679	NS
hypothetical protein	NS	2.927796	12.49941
cAMP-binding proteins - catabolite gene activator and regulatory subunit of cAM	NS	2.920308	4.96903
Isochorismatase (EC 3.3.2.1)	NS	2.910444	NS
hypothetical protein	NS	2.909161	NS
pR99_vep62	NS	2.895724	NS
UDP-Bac2Ac4Ac hydrolyzing 2-epimerase NeuC homolog	NS	2.891699	NS
HTH-type transcriptional regulator zntR	NS	2.89124	NS
Phosphorelay protein LuxU	NS	2.889789	NS
Putative two-component response regulatory protein	NS	2.888001	12.65671
hypothetical protein	NS	2.884324	NS
Dihydrofolate reductase (EC 1.5.1.3)	NS	2.872198	NS
Mannose-6-phosphate isomerase (EC 5.3.1.8)	NS	2.870261	7.617444
Phosphate regulon sensor protein PhoR (SphS) (EC 2.7.13.3)	NS	2.86852	NS
hypothetical protein	NS	2.867037	NS
Probable transcriptional activator for leuABCD operon	NS	2.863401	5.756949
hypothetical protein	NS	2.863101	NS
COG2357: Uncharacterized protein conserved in bacteria	NS	2.861655	4.435479
AraC-type DNA-binding domain-containing protein	NS	2.861213	NS
ABC-type branched-chain amino acid transport system, periplasmic component	NS	2.85732	NS
Probable Co/Zn/Cd efflux system membrane fusion protein	NS	2.848724	5.114262
hypothetical protein	NS	2.846376	NS
ABC transporter, ATP-binding protein YnjD	NS	2.837561	NS
Potassium uptake protein, integral membrane component, KtrA	NS	2.835108	3.430551
hypothetical protein	NS	2.833238	NS

Transcriptional regulator, AraC family	NS	2.832176	NS
Flagellar motor rotation protein MotB	NS	2.831627	NS
Biopolymer transport protein ExbD/TolR	NS	2.829511	8.007627
Putative exported protein	NS	2.829195	NS
hypothetical protein	NS	2.828751	NS
HflK protein	NS	2.824472	7.137057
Glutamate synthase [NADPH] large chain (EC 1.4.1.13)	NS	2.818668	NS
Serine protein kinase (prkA protein), P-loop containing	NS	2.814071	8.296692
3,4-dihydroxyphenylacetate 2,3-dioxygenase (EC 1.13.11.15)	NS	2.811926	NS
Tol biopolymer transport system, TolR protein	NS	2.805827	2.744892
Nitrite reductase [NAD(P)H] large subunit (EC 1.7.1.4)	NS	2.803775	NS
Putative inner membrane protein	NS	2.797605	NS
FOG: GGDEF domain	NS	2.793103	NS
ABC-type protease/lipase transport system, ATPase and permease component	NS	2.787721	5.498999
hypothetical protein	NS	2.780213	NS
Glutamate-1-semialdehyde aminotransferase (EC 5.4.3.8)	NS	2.777177	NS
FIG002076: hypothetical protein	NS	2.775748	6.914798
Methyl-accepting chemotaxis protein	NS	2.775341	NS
pR99_vep68	NS	2.766057	NS
Chitinase (EC 3.2.1.14)	NS	2.764302	8.847322
hypothetical protein	NS	2.759298	5.824562
hypothetical protein	NS	2.75036	NS
Dihydrolipoamide acyltransferase component of branched-chain alpha-keto acid de	NS	2.748558	NS
ABC-type sulfate transport system, permease component	NS	2.743323	8.173394
hypothetical protein	NS	2.738559	NS
hypothetical protein	NS	2.736097	2.217428
Lipase chaperone	NS	2.730495	7.979578
hypothetical protein	NS	2.72993	NS
Threonine synthase (EC 4.2.3.1)	NS	2.719443	NS
Thioredoxin 2 (EC 1.8.1.8)	NS	2.717436	NS
Peptide methionine sulfoxide reductase MsrB (EC 1.8.4.12)	NS	2.712689	NS
Bll6819 protein	NS	2.712447	8.598411
NAD-specific glutamate dehydrogenase (EC 1.4.1.2), large form	NS	2.710558	4.163602
Oligopeptide transport system permease protein OppB (TC 3.A.1.5.1)	NS	2.709386	3.142988
Lipoate synthase	NS	2.705677	2.678628
Xanthosine phosphorylase (EC 2.4.2.1)	NS	2.696094	NS
Glucosamine-link cellobiase (EC 3.2.1.21)	NS	2.695401	12.66526

hypothetical protein	NS	2.693115	5.212725
3-deoxy-D-manno-octulosonate 8-phosphate phosphatase (EC 3.1.3.45)	NS	2.688793	2.456352
hypothetical protein	NS	2.684901	NS
pR99_vep48	NS	2.681708	2.754507
Hypothetical protein, specific for Vibrio	NS	2.677738	4.868012
Transcriptional regulator	NS	2.674402	NS
hypothetical protein	NS	2.671247	3.291504
Multidrug resistance protein	NS	2.657822	NS
hypothetical protein	NS	2.656998	6.182774
GGDEF family protein	NS	2.656659	3.224478
Signal recognition particle GTPase	NS	2.656257	NS
Transcriptional regulator SlmA, TetR family	NS	2.653191	NS
hypothetical protein	NS	2.650326	4.307527
Putative oxidoreductase SMc00968	NS	2.650188	7.081549
2,3,4,5-tetrahydropyridine-2,6-dicarboxylate N-succinyltransferase (EC 2.3.1.11	NS	2.647787	NS
Methyl-accepting chemotaxis protein	NS	2.639699	2.491676
L-beta-lysine 5,6-aminomutase alpha subunit (EC 5.4.3.3)	NS	2.631856	NS
DNA polymerase III epsilon subunit (EC 2.7.7.7)	NS	2.631494	4.576823
Pyridoxamine 5'-phosphate oxidase-related, FMN-binding	NS	2.629479	5.869297
hypothetical protein	NS	2.62406	NS
Aminodeoxychorismate lyase (EC 4.1.3.38)	NS	2.621422	NS
UDP-glucose dehydrogenase (EC 1.1.1.22)	NS	2.62015	NS
pR99_vep05	NS	2.610873	3.249423
Sulfur carrier protein adenylyltransferase ThiF	NS	2.610784	NS
putative histidinol phosphatase and related hydrolases of the PHP family	NS	2.60808	2.110252
Glutamate Aspartate periplasmic binding protein precursor GltI (TC 3.A.1.3.4)	NS	2.607474	5.483985
RNA polymerase sigma factor RpoH	NS	2.604659	NS
Phosphate transport system permease protein PstA (TC 3.A.1.7.1)	NS	2.594957	NS
Multi antimicrobial extrusion protein (Na(+)/drug antiporter), MATE family of M	NS	2.572849	NS
hypothetical protein	NS	2.572751	NS
Na+/H+ antiporter NhaC	NS	2.571656	10.98525
Adenosine (5')-pentaphospho-(5")-adenosine pyrophosphohydrolase (EC 3.6.1.-)	NS	2.570361	NS
Diadenosine tetraphosphatase	NS	2.56057	NS
hypothetical protein	NS	2.555869	NS
Carboxynorspermidine decarboxylase, putative (EC 4.1.1.-)	NS	2.55469	NS
HTH-type transcriptional regulator BetI	NS	2.554616	2.949945
Transcriptional regulator	NS	2.554466	2.575646
Methyl-accepting chemotaxis protein I (serine	NS	2.554007	NS

chemoreceptor protein)			
Putative response regulator	NS	2.551205	5.784237
AttF component of AttEFGH ABC transport system / AttG component of AttEFGH ABC	NS	2.54642	NS
FOG: CheY-like receiver	NS	2.545553	4.417939
Na+/H+ antiporter NhaA type	NS	2.538639	NS
Methyl-accepting chemotaxis protein I (serine chemoreceptor protein)	NS	2.535354	NS
Anthranilate synthase, amidotransferase component (EC 4.1.3.27)	NS	2.535111	2.249749
hypothetical protein	NS	2.534178	NS
Proline/sodium symporter PutP (TC 2.A.21.2.1) @ Propionate/sodium symporter	NS	2.524558	NS
hypothetical protein	NS	2.523202	NS
Maleylacetoacetate isomerase (EC 5.2.1.2) @ Glutathione S-transferase, zeta (EC	NS	2.521686	4.920186
Bacterioferritin-associated ferredoxin	NS	2.520366	NS
Possible sterol desaturase	NS	2.511162	3.705227
hypothetical protein	NS	2.506253	5.893524
membrane protein, putative	NS	2.505901	NS
Alkaline phosphatase (EC 3.1.3.1)	NS	2.505746	6.72794
Lysine efflux permease	NS	2.504076	NS
L-xylulose 5-phosphate 3-epimerase (EC 5.1.3.-)	NS	2.499023	3.791189
Heavy-metal-associated domain (N-terminus) and membrane-bounded cytochrome biog	NS	2.497884	3.971705
Arginine pathway regulatory protein ArgR, repressor of arg regulon	NS	2.494957	NS
hypothetical protein	NS	2.488938	NS
Chitinase (EC 3.2.1.14)	NS	2.487434	NS
Methionine ABC transporter ATP-binding protein	NS	2.481513	2.190337
hypothetical protein	NS	2.477397	4.76858
Outer membrane protein YfgL, lipoprotein component of the protein assembly comp	NS	2.477043	NS
membrane protein	NS	2.47526	NS
Na+/H+ antiporter	NS	2.467256	NS
Inosine/xanthosine triphosphatase (EC 3.6.1.-); Hypothetical cytoplasmic protei	NS	2.463739	NS
hypothetical protein	NS	2.459954	NS
Ubiquinone/menaquinone biosynthesis methyltransferase UbiE (EC 2.1.1.-)	NS	2.457715	NS
Carbonic anhydrase (EC 4.2.1.1)	NS	2.455018	NS
Transcriptional regulatory protein UhpA	NS	2.453674	NS
GTP-binding protein Era	NS	2.452355	NS
putative acetyltransferase	NS	2.44591	3.910749
membrane protein	NS	2.444067	NS
Transcriptional regulator, LysR family	NS	2.432106	NS
Putative cell envelope opacity-associated protein A	NS	2.428469	NS
Transcriptional regulators, LysR family	NS	2.419833	NS

DNA mismatch repair protein MutL	NS	2.419747	NS
hypothetical protein	NS	2.419308	4.907345
Prolyl endopeptidase (EC 3.4.21.26)	NS	2.406416	NS
tRNA-specific adenosine-34 deaminase (EC 3.5.4.-)	NS	2.406218	2.104574
ribosomal protein S6 glutaminyl transferase related protein	NS	2.406183	NS
Signal peptidase I (EC 3.4.21.89)	NS	2.398126	NS
Transcriptional regulator, LacI family	NS	2.397536	5.32479
hypothetical protein	NS	2.395949	NS
DNA recombination protein RmuC	NS	2.392803	NS
hypothetical protein	NS	2.385758	NS
Ribosomal-protein-S18p-alanine acetyltransferase (EC 2.3.1.-)	NS	2.380729	3.813447
hypothetical protein	NS	2.374376	NS
Flagellin protein FlaD	NS	2.374108	NS
Transcriptional regulator of succinyl CoA synthetase operon	NS	2.373433	NS
Cytoplasmic axial filament protein CafA and Ribonuclease G (EC 3.1.4.-)	NS	2.366069	NS
UDP-N-acetylenolpyruvoylglucosamine reductase (EC 1.1.1.158)	NS	2.365217	NS
Hypothetical protein DUF454	NS	2.358943	5.471067
hypothetical protein	NS	2.358585	NS
Permeases of the major facilitator superfamily	NS	2.356622	3.567894
Multidrug efflux pump component MtrF	NS	2.355003	NS
pR99_vep69	NS	2.348404	4.226203
FIG000506: Hypothetical ATP-binding protein	NS	2.341046	NS
hypothetical protein	NS	2.340587	NS
Gamma-glutamyl phosphate reductase (EC 1.2.1.41)	NS	2.335087	NS
Probable transcriptional activator for leuABCD operon	NS	2.33007	5.404777
Fumarylacetoacetase (EC 3.7.1.2)	NS	2.329993	5.300276
AttH component of AttEFGH ABC transport system	NS	2.329902	NS
TRAP dicarboxylate transporter, DctQ subunit, unknown substrate 3	NS	2.327301	6.803001
Putative threonine efflux protein	NS	2.325649	NS
hypothetical protein	NS	2.324674	6.318505
hypothetical protein	NS	2.321689	4.461745
Histone acetyltransferase HPA2	NS	2.31833	6.424567
Leader peptidase (Prepilin peptidase) (EC 3.4.23.43) / N-methyltransferase (EC	NS	2.313916	4.352097
SAM-dependent methyltransferases	NS	2.310031	NS
pR99_vep24	NS	2.298411	3.793781
Molybdenum cofactor biosynthesis protein MoaC	NS	2.295287	2.2128
Inner membrane protein translocase component YidC, long form	NS	2.293224	NS

Legionaminic acid synthase (EC 2.5.1.56)	NS	2.290835	NS
hypothetical protein	NS	2.289337	NS
Spermidine synthase-like protein	NS	2.288979	NS
Potassium uptake protein TrkH	NS	2.288678	3.920855
UPF0246 protein YaaA	NS	2.285596	NS
Transcriptional regulator, LysR family	NS	2.283018	NS
Cell division inhibitor	NS	2.277669	NS
L-proline glycine betaine ABC transport system permease protein ProV (TC 3.A.1.	NS	2.271382	NS
Acylphosphate phosphohydrolase (EC 3.6.1.7), putative	NS	2.271022	NS
Transcriptional regulator, TetR family	NS	2.266218	NS
ABC transporter ATP-binding protein YvcR	NS	2.261556	2.85295
hypothetical protein	NS	2.259661	NS
Outer membrane protein SypB	NS	2.256912	5.214192
BarA-associated response regulator UvrY (= GacA = SirA)	NS	2.256182	NS
Chorismate synthase (EC 4.2.3.5)	NS	2.256082	NS
Transcriptional regulator, ArsR family	NS	2.254006	NS
Flagellin protein FlaG	NS	2.251123	6.460588
MSHA biogenesis protein MshP	NS	2.243086	4.245048
RTX toxin	NS	2.236491	7.168073
Octanoate-[acyl-carrier-protein]-protein-N-octanoyltransferase	NS	2.235435	NS
Soluble lytic murein transglycosylase precursor (EC 3.2.1.-)	NS	2.228645	NS
Protein-export membrane protein SecD (TC 3.A.5.1.1)	NS	2.227985	NS
Signal transduction histidine kinase	NS	2.21872	7.709089
Predicted methylated DNA-protein cysteine methyltransferase	NS	2.210761	NS
Flagellin protein FlaD	NS	2.210231	NS
transposase and inactivated derivative	NS	2.209231	NS
Cobyric acid synthase	NS	2.203523	NS
5,10-methylenetetrahydrofolate reductase (EC 1.5.1.20)	NS	2.202977	NS
Transcriptional regulator, AraC family	NS	2.201165	5.460703
hypothetical protein	NS	2.200806	NS
UDP-N-acetylmuramoylalanine--D-glutamate ligase (EC 6.3.2.9)	NS	2.19873	NS
tRNA-(ms[2]io[6]A)-hydroxylase (EC 1.-.-.-)	NS	2.197636	4.792256
Uncharacterized protein	NS	2.195206	NS
Permease of the major facilitator superfamily	NS	2.190513	NS
Putative magnesium transporter MgtE	NS	2.190163	NS
hypothetical protein	NS	2.189556	4.654007
Flagellar motor switch protein FliM	NS	2.186882	2.552607
ABC-type uncharacterized transport system, periplasmic component	NS	2.184181	4.879006

Sulfur carrier protein ThiS	NS	2.179821	NS
pR99_vep27	NS	2.17744	NS
Exodeoxyribonuclease V alpha chain (EC 3.1.11.5) ## RecD	NS	2.176843	3.011676
hypothetical protein	NS	2.174352	2.336989
Outer membrane protein C precursor	NS	2.171663	NS
ElaA protein	NS	2.168774	NS
hypothetical protein	NS	2.164872	NS
Multidrug resistance protein D	NS	2.160618	NS
hypothetical protein	NS	2.153939	NS
ADP-heptose synthase (EC 2.7.-.-) / D-glycero-beta-D-manno-heptose 7-phosphate	NS	2.144588	NS
AsmA protein	NS	2.135913	NS
Chaperone protein DnaJ	NS	2.13437	NS
hypothetical protein	NS	2.129695	NS
Putative threonine efflux protein	NS	2.12641	NS
Protein of unknown function Smg	NS	2.124139	NS
hypothetical protein	NS	2.123389	5.543967
Methylmalonate-semialdehyde dehydrogenase (EC 1.2.1.27)	NS	2.122855	NS
MSHA biogenesis protein MshJ	NS	2.117128	NS
ATPase of the AAA+ class	NS	2.112419	-2.19455
Uncharacterized protein conserved in bacteria, NMA0228-like	NS	2.112308	NS
Guanylate cyclase-related protein	NS	2.107426	5.195814
ATP-dependent Clp protease ATP-binding subunit ClpX	NS	2.105531	NS
Protein YigP (COG3165) clustered with ubiquinone biosynthetic genes	NS	2.103728	2.024202
Bacterioferritin	NS	2.095396	2.256245
Fumarate and nitrate reduction regulatory protein	NS	2.087426	3.952053
Membrane-fusion protein	NS	2.086677	NS
CMP-N-acetylneuraminate-beta-galactosamide-alpha-2,3-sialyltransferase (EC 2.4.	NS	2.080221	NS
3-polyprenyl-4-hydroxybenzoate carboxy-lyase UbiX (EC 4.1.1.-)	NS	2.079277	2.067552
Putative multidrug resistance protein	NS	2.075142	NS
Functional role page for TorCAD operon transcriptional regulatory protein TorR	NS	2.074838	NS
Argininosuccinate synthase (EC 6.3.4.5)	NS	2.072695	NS
hypothetical protein	NS	2.071791	NS
General secretion pathway protein J	NS	2.068735	NS
Molybdenum transport system permease protein ModB (TC 3.A.1.8.1)	NS	2.06844	3.394163
hypothetical protein	NS	2.064575	NS
pR99_vep70	NS	2.063749	5.020094
hypothetical protein	NS	2.061655	NS
Siroheme synthase / Precorrin-2 oxidase (EC	NS	2.05749	NS

1.3.1.76) / Sirohydrochlorin ferroc			
hypothetical protein	NS	2.0542	2.831558
hypothetical protein	NS	2.050568	NS
hypothetical protein	NS	2.046493	NS
Ferrous iron transport protein B	NS	2.0435	NS
2-keto-3-deoxy-D-arabino-heptulosonate-7-phosphate synthase I alpha (EC 2.5.1.5	NS	2.042349	NS
hypothetical protein	NS	2.041059	NS
N-Ribosylnicotinamide phosphorylase (EC 2.4.2.1)	NS	2.040473	NS
ABC transporter, periplasmic spermidine putrescine-binding protein PotD (TC 3.A	NS	2.038611	NS
1,4-alpha-glucan branching enzyme (EC 2.4.1.18)	NS	2.038285	NS
Transcriptional regulator	NS	2.037723	NS
hypothetical protein	NS	2.034636	NS
POTASSIUM/PROTON ANTIPORTER ROSB	NS	2.033561	NS
Glutamine amidotransferases class-II	NS	2.030247	2.876468
hypothetical protein	NS	2.030123	NS
hypothetical protein	NS	2.029627	NS
COG4123: Predicted O-methyltransferase	NS	2.029553	NS
Transcriptional regulator, TetR family	NS	2.026235	NS
membrane protein	NS	2.02445	5.340091
FIG005080: Possible exported protein	NS	2.022896	NS
Membrane-associated phospholipid phosphatase	NS	2.022812	NS
Oxalate/formate antiporter	NS	2.018493	NS
Gluconate utilization system Gnt-I transcriptional repressor	NS	2.01386	NS
transposase	NS	2.009301	NS
CheW domain protein	NS	2.006153	NS
Type IV pilin PilA	NS	2.005938	NS
High-affinity choline uptake protein BetT	NS	2.005398	NS
ADP-L-glycero-D-manno-heptose-6-epimerase (EC 5.1.3.20)	NS	2.002966	NS
hypothetical protein	NS	-2.00413	NS
Glutaredoxin	NS	-2.0063	NS
Putative signal peptide protein	NS	-2.01231	NS
tRNA (cytosine34-2'-O-)-methyltransferase (EC 2.1.1.-)	NS	-2.01379	NS
Putrescine aminotransferase (EC 2.6.1.82)	NS	-2.0156	NS
hypothetical protein	NS	-2.01724	NS
Na(+)-translocating NADH-quinone reductase subunit F (EC 1.6.5.-)	NS	-2.0215	NS
hypothetical protein	NS	-2.0246	NS
hypothetical protein	NS	-2.0274	NS
Probable low-affinity inorganic phosphate transporter	NS	-2.0384	-2.03604
Membrane alanine aminopeptidase N (EC 3.4.11.2)	NS	-2.05	NS
hypothetical protein	NS	-2.05009	NS

Ferritin-like protein 2	NS	-2.05611	-2.09919
Predicted sodium/dicarboxylate symporter	NS	-2.07121	NS
Histidinol-phosphatase (EC 3.1.3.15) / Imidazoleglycerol-phosphate dehydratase	NS	-2.08933	NS
ATP-dependent RNA helicase RhlE	NS	-2.09072	NS
SSU ribosomal protein S9p (S16e)	NS	-2.09072	NS
NAD(FAD)-utilizing dehydrogenase, sll0175 homolog	NS	-2.09273	NS
hypothetical protein	NS	-2.11339	NS
Phosphoribosylformimino-5-aminoimidazole carboxamide ribotide isomerase (EC 5.3	NS	-2.11418	NS
3-oxoacyl-[acyl-carrier-protein] synthase, KASIII (EC 2.3.1.41)	NS	-2.11791	-2.28884
Pantoate--beta-alanine ligase (EC 6.3.2.1)	NS	-2.11858	NS
hypothetical protein	NS	-2.12597	NS
Uncharacterized low-complexity protein	NS	-2.13053	NS
Probable exported or periplasmic protein in ApbE locus	NS	-2.13055	NS
Transcriptional regulator, LysR family	NS	-2.1357	NS
Uncharacterized ABC transporter, auxiliary component YrbC	NS	-2.15563	NS
Small-conductance mechanosensitive channel	NS	-2.15661	NS
DNA topoisomerase III (EC 5.99.1.2)	NS	-2.16059	NS
Alanyl-tRNA synthetase (EC 6.1.1.7)	NS	-2.16889	NS
Oxygen-insensitive NAD(P)H nitroreductase (EC 1.-.-.-) / Dihydropteridine reduc	NS	-2.16969	NS
N-acetylglucosamine-6-phosphate deacetylase (EC 3.5.1.25)	NS	-2.18535	-2.20124
Transcriptional activator RfaH	NS	-2.19156	-2.85685
Methylase of polypeptide chain release factors	NS	-2.20951	NS
hypothetical protein	NS	-2.21187	NS
Syd protein	NS	-2.23083	NS
Transcription termination factor Rho	NS	-2.23133	NS
Histone acetyltransferase HPA2 and related acetyltransferases	NS	-2.23946	NS
Endonuclease IV (EC 3.1.21.2)	NS	-2.24686	NS
Cytosine deaminase (EC 3.5.4.1)	NS	-2.25524	NS
Uncharacterized conserved protein	NS	-2.26181	NS
hypothetical protein	NS	-2.26476	NS
Putative threonine efflux protein	NS	-2.26851	NS
Phosphogluconate repressor HexR, RpiR family	NS	-2.28078	NS
Methyltransferase (EC 2.1.1.-)	NS	-2.28704	NS
hypothetical protein	NS	-2.29278	NS
UDP-N-acetylmuramate--alanine ligase (EC 6.3.2.8)	NS	-2.31306	NS
DNA-binding protein HU-beta	NS	-2.34552	-2.69544
Topoisomerase IV subunit A (EC 5.99.1.-)	NS	-2.34863	NS
RNA methyltransferase, TrmA family	NS	-2.34919	NS

Glutaminase (EC 3.5.1.2)	NS	-2.34932	NS
Transposase	NS	-2.35112	-3.39127
3-oxoacyl-[acyl-carrier protein] reductase (EC 1.1.1.100)	NS	-2.36025	NS
Na(+)-translocating NADH-quinone reductase subunit E (EC 1.6.5.-)	NS	-2.38583	NS
YrbA protein	NS	-2.39405	NS
Putative chemotaxis protein CheY	NS	-2.39516	NS
6-phosphofructokinase (EC 2.7.1.11)	NS	-2.39977	NS
Acyl-CoA thioesterase YciA, involved in membrane biogenesis	NS	-2.44041	NS
S-ribosylhomocysteine lyase (EC 4.4.1.21) / Autoinducer-2 production protein Lu	NS	-2.44792	-3.61063
hypothetical protein	NS	-2.45835	NS
Putative cytoplasmic protein	NS	-2.4695	-2.15357
Hypothetical protein VC0266 (sugar utilization related?)	NS	-2.48445	NS
Thiamine biosynthesis protein thiI	NS	-2.51552	-2.92271
hypothetical protein	NS	-2.53899	NS
Shikimate kinase I (EC 2.7.1.71)	NS	-2.55097	NS
Acyl carrier protein (ACP1)	NS	-2.56717	NS
Rod shape-determining protein MreB	NS	-2.58048	NS
hypothetical protein	NS	-2.58115	NS
Cytochrome c-type protein NrfB precursor	NS	-2.5977	NS
Alcohol dehydrogenase (EC 1.1.1.1)	NS	-2.60336	NS
Autoinducer 2 sensor kinase/phosphatase LuxQ (EC 2.7.3.-) (EC 3.1.3.-)	NS	-2.61961	NS
OsmC/Ohr family protein	NS	-2.63154	-3.65153
Phospho-N-acetylmuramoyl-pentapeptide-transferase (EC 2.7.8.13)	NS	-2.63324	-2.36987
Nitrogen regulation protein NR(II) (EC 2.7.3.-)	NS	-2.65989	NS
Putative cytochrome d ubiquinol oxidase subunit III (EC 1.10.3.-) (Cytochrome b	NS	-2.66381	-2.37809
hypothetical protein	NS	-2.67852	-3.26498
ABC transporter, periplasmic spermidine putrescine-binding protein PotD (TC 3.A	NS	-2.71776	NS
S-adenosylmethionine:tRNA ribosyltransferase-isomerase (EC 5.-.-.-)	NS	-2.74479	NS
Methyl-accepting chemotaxis protein	NS	-2.77203	NS
Polysaccharide export lipoprotein Wza	NS	-2.82433	NS
Transcriptional regulator, TetR family	NS	-2.82444	NS
2-keto-4-pentenoate hydratase (EC 4.2.1.-)	NS	-2.84006	-2.65423
Putative sugar isomerase involved in processing of exogenous sialic acid	NS	-2.84198	NS
Acyl carrier protein (ACP2)	NS	-3.27431	NS
hypothetical protein	NS	-3.31165	NS
Cell division protein FtsK	NS	-3.35495	NS
Ribose ABC transport system, ATP-binding protein RbsA (TC 3.A.1.2.1)	NS	-3.39203	NS

hypothetical protein	NS	-3.41432	NS
Cytochrome c553	NS	-3.63877	NS
Histone acetyltransferase HPA2	NS	-3.82384	-4.97171
hypothetical protein	NS	-4.28988	NS
hypothetical protein	NS	NS	40.57813
Cytochrome c-type heme lyase subunit nrfF, nitrite reductase complex assembly	NS	NS	23.34484
Acetyltransferase	NS	NS	21.89566
hypothetical protein	NS	NS	19.94106
Acetyl-coenzyme A synthetase (EC 6.2.1.1)	NS	NS	19.67235
Oxidoreductase, short-chain dehydrogenase/reductase family (EC 1.1.1.-)	NS	NS	16.76826
hypothetical protein	NS	NS	16.64664
Putative transcription antitermination protein NusG	NS	NS	15.58195
Methyl-accepting chemotaxis protein	NS	NS	15.49277
hypothetical protein	NS	NS	15.36196
Cobalt-zinc-cadmium resistance protein CzcA; Cation efflux system protein CusA	NS	NS	15.10143
hypothetical protein	NS	NS	14.73546
ABC-type amino acid transport/signal transduction systems, periplasmic componen	NS	NS	13.8118
hypothetical protein	NS	NS	13.64972
TRAP transporter solute receptor, TAXI family precursor	NS	NS	13.45874
hypothetical protein	NS	NS	12.89311
Glycogen debranching enzyme	NS	NS	12.81456
sodium-solute symporter, putative	NS	NS	12.23475
TonB system biopolymer transport component; Chromosome segregation ATPase	NS	NS	12.19394
hypothetical protein	NS	NS	12.09114
Flagellar P-ring protein FlgI	NS	NS	11.87757
Permease of the drug/metabolite transporter (DMT) superfamily	NS	NS	11.53117
hypothetical protein	NS	NS	11.51985
Methyl-accepting chemotaxis protein I (serine chemoreceptor protein)	NS	NS	11.23461
Glucosamine kinase GpsK (EC 2.7.1.8)	NS	NS	11.06769
Homogentisate 1,2-dioxygenase (EC 1.13.11.5)	NS	NS	10.37688
Similarity with glutathionylspermidine synthase (EC 6.3.1.8), group 1	NS	NS	10.15181
Anti-anti-sigma regulatory factor	NS	NS	10.0722
(GlcNAc)2 ABC transporter, ATP-binding component 1	NS	NS	9.990188
hypothetical protein	NS	NS	9.418215
hypothetical protein	NS	NS	9.395066
3-hydroxyisobutyrate dehydrogenase (EC 1.1.1.31)	NS	NS	9.241655
hypothetical protein	NS	NS	8.842976
hypothetical protein	NS	NS	8.807178

hypothetical protein	NS	NS	8.331194
Transcriptional regulatory protein CitB, DpiA	NS	NS	8.325768
Myo-inositol 2-dehydrogenase (EC 1.1.1.18)	NS	NS	8.253069
Oligopeptide transport ATP-binding protein OppF (TC 3.A.1.5.1)	NS	NS	7.924672
Aldose 1-epimerase (EC 5.1.3.3)	NS	NS	7.62251
Methyl-accepting chemotaxis protein I (serine chemoreceptor protein)	NS	NS	7.321003
hypothetical protein	NS	NS	7.23703
hypothetical protein	NS	NS	7.057282
RarD protein	NS	NS	7.041554
hypothetical protein	NS	NS	7.02957
hypothetical protein	NS	NS	6.689141
ABC-type amino acid transport, signal transduction systems, periplasmic compone	NS	NS	6.633903
Ribose ABC transport system, periplasmic ribose-binding protein RbsB (TC 3.A.1.	NS	NS	6.60265
COG1496: Uncharacterized conserved protein	NS	NS	6.454139
Uncharacterized protein, probably surface-located	NS	NS	6.407074
ABC-type amino acid transport, signal transduction systems, periplasmic compone	NS	NS	6.187115
hypothetical protein	NS	NS	6.083755
Uncharacterized conserved protein	NS	NS	5.994946
hypothetical protein	NS	NS	5.957518
hypothetical protein	NS	NS	5.823073
Putative response regulator	NS	NS	5.760142
membrane protein	NS	NS	5.737436
FIG004684: SpoVR-like protein	NS	NS	5.596253
Hypothetical protein, ydbT homolog	NS	NS	5.532622
LSU ribosomal protein L31p	NS	NS	5.43183
FIG003276: zinc-binding protein	NS	NS	5.37389
Lipase precursor (EC 3.1.1.3)	NS	NS	5.252189
hypothetical protein	NS	NS	5.234456
Adenylosuccinate synthetase (EC 6.3.4.4)	NS	NS	5.209096
hypothetical protein	NS	NS	5.207097
Transcriptional regulator VpsT	NS	NS	5.113505
hypothetical protein	NS	NS	5.050263
Thiamin biosynthesis protein ThiC	NS	NS	5.013849
BatD	NS	NS	4.943864
Response regulator	NS	NS	4.943441
Galactose-1-phosphate uridylyltransferase (EC 2.7.7.10)	NS	NS	4.919249
Mu-like prophage protein gp16	NS	NS	4.842106
Phosphomethylpyrimidine kinase (EC 2.7.4.7)	NS	NS	4.759375
Aryl carrier domain	NS	NS	4.748046
Outer membrane receptor protein	NS	NS	4.637283
hypothetical protein	NS	NS	4.608804

hypothetical protein	NS	NS	4.519175
putative transcriptional regulator, XRE family	NS	NS	4.485213
Cell division protein FtsL	NS	NS	4.483848
hypothetical protein	NS	NS	4.457958
Ubiquitin-protein ligase	NS	NS	4.400348
hypothetical protein	NS	NS	4.378367
Isocitrate lyase (EC 4.1.3.1)	NS	NS	4.360288
Periplasmic thiol:disulfide interchange protein DsbA	NS	NS	4.354997
ABC-type sugar transport system, periplasmic component	NS	NS	4.336171
Thymidine kinase (EC 2.7.1.21)	NS	NS	4.301212
Glucose-1-phosphate adenylyltransferase (EC 2.7.7.27)	NS	NS	4.298542
UDP-glucose 4-epimerase (EC 5.1.3.2)	NS	NS	4.262126
Periplasmic protein torT precursor	NS	NS	4.223666
Sensor histidine kinase	NS	NS	4.212334
hypothetical protein	NS	NS	4.208261
hypothetical protein	NS	NS	4.206581
Potassium voltage-gated channel subfamily KQT; possible potassium channel, VIC	NS	NS	4.187073
TonB-dependent receptor	NS	NS	4.168027
CofC, F420 2-Phospho-l-lactate Guanylyltransferase	NS	NS	4.161749
ABC transporter substrate-binding protein	NS	NS	4.150379
Betaine aldehyde dehydrogenase (EC 1.2.1.8)	NS	NS	4.142733
Ribosome recycling factor	NS	NS	4.115886
Putative transcriptional regulator	NS	NS	4.103647
Predicted ABC-type transport system, permease component	NS	NS	4.097736
Secreted trypsin-like serine protease	NS	NS	4.087142
Response regulator	NS	NS	4.056992
Ornithine racemase (EC 5.1.1.12)	NS	NS	4.049523
Uncharacterized domain COG3236 / GTP cyclohydrolase II (EC 3.5.4.25)	NS	NS	4.022832
Paraquat-inducible protein B	NS	NS	4.014012
DNA uptake protein	NS	NS	3.963569
Transcriptional regulator, LysR family	NS	NS	3.929414
Polyferredoxin NapH (periplasmic nitrate reductase)	NS	NS	3.915314
Putative analog of CcoH, COG3198	NS	NS	3.908808
hypothetical protein	NS	NS	3.891768
FOG: EAL domain protein	NS	NS	3.888689
hypothetical protein	NS	NS	3.86717
Na+-driven multidrug efflux pump	NS	NS	3.820611
hypothetical protein	NS	NS	3.751614
FOG: EAL domain protein	NS	NS	3.731755

Flagellar protein FlgT	NS	NS	3.715738
hypothetical protein	NS	NS	3.687056
Methyl-accepting chemotaxis protein I (serine chemoreceptor protein)	NS	NS	3.658496
Outer membrane receptor protein	NS	NS	3.658016
Methylated-DNA--protein-cysteine methyltransferase (EC 2.1.1.63)	NS	NS	3.657455
Putative transporter	NS	NS	3.654822
hypothetical protein	NS	NS	3.624347
Ribonuclease BN (EC 3.1.-.-)	NS	NS	3.620564
Malate synthase-related protein	NS	NS	3.616631
Signal transduction histidine kinase	NS	NS	3.608113
Para-aminobenzoate synthase, aminase component (EC 2.6.1.85)	NS	NS	3.560487
Predicted transcriptional regulator	NS	NS	3.555195
PTS system, N-acetylmuramic acid-specific IIB component (EC 2.7.1.69) / PTS sys	NS	NS	3.529165
Translation initiation factor 1	NS	NS	3.511509
Ribose ABC transport system, permease protein RbsC (TC 3.A.1.2.1)	NS	NS	3.449628
Plasmid-related protein	NS	NS	3.444193
ABC transporter ATP-binding protein YvcR	NS	NS	3.437671
hypothetical protein	NS	NS	3.430596
hypothetical protein	NS	NS	3.403671
Helicase	NS	NS	3.38052
Na+-driven multidrug efflux pump	NS	NS	3.359135
hypothetical protein	NS	NS	3.32867
Acetyltransferase (EC 2.3.1.-)	NS	NS	3.271632
hypothetical protein	NS	NS	3.269245
hypothetical protein	NS	NS	3.25871
hypothetical protein	NS	NS	3.241409
hypothetical protein	NS	NS	3.237882
54K polar flagellar sheath protein A	NS	NS	3.225971
Permease of the major facilitator superfamily	NS	NS	3.21407
hypothetical protein	NS	NS	3.190803
Protein sprT	NS	NS	3.190016
hypothetical protein	NS	NS	3.177227
hypothetical protein	NS	NS	3.16408
hypothetical protein	NS	NS	3.150195
Hemolysins and related proteins containing CBS domains	NS	NS	3.13589
Predicted hydrolase	NS	NS	3.129382
hypothetical protein	NS	NS	3.09669
General secretion pathway protein A / General secretion pathway protein B	NS	NS	3.09649
Transcriptional regulator, LysR family	NS	NS	3.091616
hypothetical protein	NS	NS	2.991112

Thiamine kinase (EC 2.7.1.89) @ Adenosylcobinamide kinase (EC 2.7.1.156)	NS	NS	2.987948
Glycerate kinase (EC 2.7.1.31)	NS	NS	2.978392
Cell division protein FtsX	NS	NS	2.974154
General secretion pathway protein H	NS	NS	2.933158
tRNA pseudouridine synthase C (EC 4.2.1.70) ## tRNA Psi65	NS	NS	2.920592
Fructose-specific phosphocarrier protein HPr (EC 2.7.1.69) / PTS system, fructo	NS	NS	2.84102
MFS family multidrug transport protein, bicyclomycin resistance protein	NS	NS	2.839313
Transposase	NS	NS	2.836619
predicted 4-deoxy-L-threo-5-hexosulose-uronate ketol-isomerase (EC 5.3.1.17)	NS	NS	2.829498
Beta-galactosidase/beta-glucuronidase	NS	NS	2.823232
Ferredoxin-type protein NapG (periplasmic nitrate reductase)	NS	NS	2.821603
UDP-2,3-diacylglucosamine hydrolase (EC 3.6.1.-)	NS	NS	2.820932
Sensor kinase CitA, DpiB (EC 2.7.3.-)	NS	NS	2.818013
YjeF protein, function unknown	NS	NS	2.813876
hypothetical protein	NS	NS	2.811969
transposase and inactivated derivative	NS	NS	2.804039
Ribosomal large subunit pseudouridine synthase D (EC 4.2.1.70)	NS	NS	2.803811
hypothetical protein	NS	NS	2.79125
Transcriptional regulator, AraC family	NS	NS	2.779296
two-component system sensor protein	NS	NS	2.759883
Inactive homolog of metal-dependent proteases, putative molecular chaperone	NS	NS	2.753046
Cobalt-zinc-cadmium resistance protein	NS	NS	2.74972
HDIG domain protein	NS	NS	2.745595
Molybdenum transport ATP-binding protein ModC (TC 3.A.1.8.1)	NS	NS	2.705007
1-hydroxy-2-methyl-2-(E)-butenyl 4-diphosphate synthase (EC 1.17.7.1)	NS	NS	2.695832
Lipid A biosynthesis lauroyl acyltransferase (EC 2.3.1.-)	NS	NS	2.689616
Proposed peptidoglycan lipid II flippase MurJ	NS	NS	2.631409
hypothetical protein	NS	NS	2.612714
Signal transduction histidine kinase	NS	NS	2.602679
hypothetical protein	NS	NS	2.60158
DNA mismatch repair endonuclease MutH	NS	NS	2.590339
Rare lipoprotein A precursor	NS	NS	2.552086
Transposase	NS	NS	2.542232
Uncharacterized protein conserved in bacteria	NS	NS	2.534266
Methionine ABC transporter ATP-binding protein	NS	NS	2.532356
hypothetical protein	NS	NS	2.514924
Chitin catabolic cascade sensor histidine kinase ChiS	NS	NS	2.507782

membrane protein	NS	NS	2.501841
hypothetical protein	NS	NS	2.464586
RNA polymerase sigma factor SigZ	NS	NS	2.459373
1-phosphofructokinase (EC 2.7.1.56)	NS	NS	2.455149
Membrane protein YcjF	NS	NS	2.446014
PTS system, beta-glucoside-specific IIB component (EC 2.7.1.69) / PTS system, b	NS	NS	2.409239
Glutathione S-transferase, omega (EC 2.5.1.18)	NS	NS	2.381643
Cell division transporter, ATP-binding protein FtsE (TC 3.A.5.1.1)	NS	NS	2.367229
ATPase involved in DNA repair	NS	NS	2.356725
DNA polymerase IV (EC 2.7.7.7)	NS	NS	2.34745
putative outer membrane lipoprotein	NS	NS	2.347323
hypothetical protein	NS	NS	2.345797
hypothetical protein	NS	NS	2.333952
hypothetical protein	NS	NS	2.333308
FIG002708: Protein SirB1	NS	NS	2.302374
Peptide methionine sulfoxide reductase MsrA (EC 1.8.4.11) / Peptide methionine	NS	NS	2.291341
Magnesium transporter	NS	NS	2.289875
hypothetical protein	NS	NS	2.285325
LptA, protein essential for LPS transport across the periplasm	NS	NS	2.281172
Glutaredoxin	NS	NS	2.262832
3-oxoacyl-[ACP] synthase	NS	NS	2.259259
Chromosome partition protein MukF	NS	NS	2.257924
hypothetical protein	NS	NS	2.24234
Transcriptional regulator, LuxR family	NS	NS	2.241995
putative cytoplasmic protein	NS	NS	2.213363
pR99_traI	NS	NS	2.205189
FOG: EAL domain protein	NS	NS	2.189215
Glutamate racemase (EC 5.1.1.3)	NS	NS	2.179024
hypothetical protein	NS	NS	2.173043
Undecaprenyl pyrophosphate synthetase (EC 2.5.1.31)	NS	NS	2.158285
Sensor histidine kinase	NS	NS	2.156147
DNA polymerase III chi subunit (EC 2.7.7.7)	NS	NS	2.155047
hypothetical protein	NS	NS	2.154121
Transposase	NS	NS	2.141375
MSHA biogenesis protein MshK	NS	NS	2.140836
Oligopeptide transport ATP-binding protein OppD (TC 3.A.1.5.1)	NS	NS	2.136146
pR99_vep66	NS	NS	2.131127
Outer membrane stress sensor protease DegQ, serine protease	NS	NS	2.106282
GGDEF family protein	NS	NS	2.093953
Phosphoheptose isomerase (EC 5.3.1.-)	NS	NS	2.093065

Transcriptional regulator, MerR family	NS	NS	2.07802
FIG000906: Predicted Permease	NS	NS	2.072972
rRNA small subunit methyltransferase I	NS	NS	2.061767
Bacillosamine/Legionaminic acid biosynthesis aminotransferase PglE; 4-keto-6-de	NS	NS	2.000212
membrane protein	NS	NS	-2.08785
Cell division protein BolA	NS	NS	-2.15759
Cell wall-associated hydrolase	NS	NS	-2.16122
hypothetical protein	NS	NS	-2.20804
Positive regulator of CheA protein activity (CheW)	NS	NS	-2.39104
FOG: TPR repeat protein	NS	NS	-2.66211
Unsaturated fatty acid biosythesis repressor FabR, TetR family	NS	NS	-2.68226
hypothetical protein	NS	NS	-2.76691
RNA polymerase sigma-70 factor, ECF subfamily	NS	NS	-3.09961
GMP synthase [glutamine-hydrolyzing] (EC 6.3.5.2)	NS	NS	-3.15306
Dihydroorotate dehydrogenase (EC 1.3.3.1)	NS	NS	-3.3379
